# Antiparasitic Activities of Acyl Hydrazones from Cinnamaldehydes and Structurally Related Fragrances

**DOI:** 10.3390/antibiotics13121114

**Published:** 2024-11-22

**Authors:** Ibrahim S. Al Nasr, Waleed S. Koko, Tariq A. Khan, Rainer Schobert, Bernhard Biersack

**Affiliations:** 1 Department of Biology, College of Science, Qassim University, Qassim 51452, Saudi Arabia; insar@qu.edu.sa (I.S.A.N.); wasyko2002@yahoo.com (W.S.K.); 2 Department of Basic Health Sciences, College of Applied Medical Sciences, Qassim University, Qassim 51452, Saudi Arabia; sirtariqayub@gmail.com; 3 Organic Chemistry Laboratory, University Bayreuth, Universitätsstrasse 30, 95440 Bayreuth, Germany; rainer.schobert@uni-bayreuth.de

**Keywords:** cinnamaldehyde, fragrances, acyl hydrazones, antiparasitic drugs, toxoplasmosis, *Toxoplasma gondii*, leishmaniasis, *Leishmania major*

## Abstract

**Background:** New drugs for the treatment of protozoal parasite infections such as toxoplasmosis and leishmaniasis are required. Cinnamaldehyde and its derivatives appear to be promising antiparasitic drug candidates. **Methods:** Acyl hydrazones of cinnamaldehyde, 4-dimethylaminocinnamaldehyde, and of the synthetic fragrances silvial^TM^ and florhydral^TM^ were prepared and tested for activity against *Toxoplasma gondii* (*T. gondii*) and *Leishmania major* (*L. major*) parasites. **Results:** Three cinnamaldehyde acyl hydrazones (3-hydroxy-2-naphthoyl **2a** and the salicyloyls **2c** and **2d**) showed good activity against *T. gondii*, and two compounds derived from cinnamaldehyde and florhydral^TM^ (3-hydroxy-2-naphthoyls **2a** and **4a**) exhibited moderate activity against *L. major* promastigotes. **Conclusions:** In particular, the identified antitoxoplasmal activities are promising and might lead to the development of new potent and cost-effective drug candidates for the therapy of toxoplasmosis.

## 1. Introduction

The search for efficient and well-tolerated drugs for the therapy of perilous infectious diseases is ongoing. The apicomplexan parasite agent of toxoplasmosis, *Toxoplasma gondii*, occurs globally and poses a considerable health risk to vulnerable immunocompromised persons, such as people with HIV, organ transplant patients, and neonates [1,2,3]. Sulfonamides (e.g., sulfadiazine), DHFR inhibitors (pyrimethamine and trimethoprim), and the naphthoquinone atovaquone are the currently available antiparasitic drugs for toxoplasmosis patients [4]. However, adverse effects such as nephrotoxicity and the identification of drug-resistant clinical isolates necessitate the development of new drugs [5,6]. Likewise, leishmaniasis is an infectious tropical disease with limited or even toxic treatment options. The kinetoplastid *Leishmania* parasites can cause severe forms of infections such as visceral leishmaniasis, cutaneous leishmaniasis (the most abundant manifestation with up to 1 million cases), and mucocutaneous leishmaniasis [7]. *L. major* and *L. tropica* species are the causative agents of Old World cutaneous leishmaniasis which differ in their sensitivity to antimony drugs [8]. In order to replace toxic antimonials and expensive polyene drugs such as amphotericin B in leishmaniasis therapy, the development of new antileishmanials is necessary [9]. For instance, the volatile oil of *Cinnamomum cassia* has recently been shown to possess pronounced antileishmanial activity [10].

**Figure 1:**
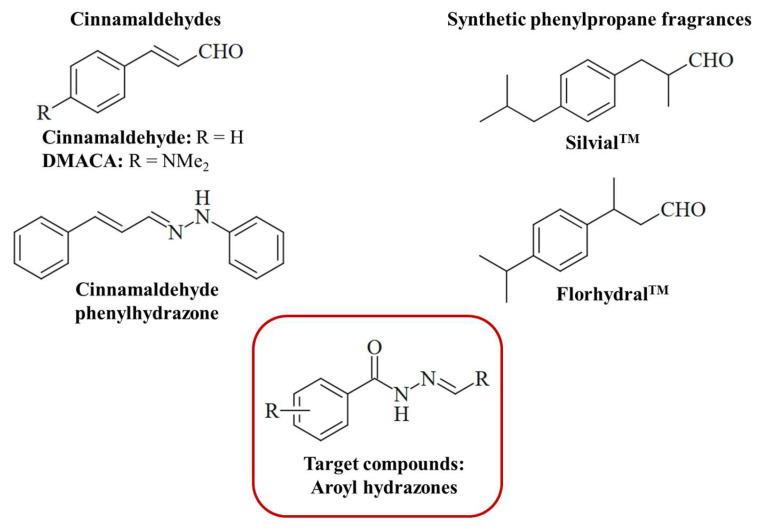
Structures of the cinnamaldehydes and synthetic phenylpropane fragrances (silvial^TM^ and florhydral^TM^), as well as the general structure of the aroyl hydrazone target compounds prepared in this study.

*Cinnamomum* plants are aromatic trees and shrubs of the tropical and sub-tropical regions of Asia and the Western Pacific and are used as spices, flavors, fragrances, and traditional drugs [11]. *C. verum* (*Ceylon cinnamon*) and *C. cassia* (*Chinese cinnamon*) are the major sources of the valuable cinnamon spice, which is of great importance in the food industry, while traditional healers use cinnamon bark for the treatment of diabetes, digestive disorders, and lung infections [12]. The essential oil (EO) of cinnamon bark is characterized by a high content of the biologically active phenylpropanoid cinnamaldehyde, also known as (2*E*)-3-phenylprop-2-enal (Figure 1) [13]. The biosynthesis of cinnamaldehyde starts from L-phenylalanine, which undergoes phenylalanine ammonia lyase (PAL)-catalyzed deamination to cinnamic acid followed by ATP-dependent 4-coumarate-CoA ligase (4CL)-catalyzed formation of cinnamoyl-CoA and cinnamoyl-CoA reductase (CCR)-mediated reduction to the aldehyde with NADPH [14]. Cinnamaldehyde is a sweet yellowish oil and exudes the characteristic cinnamon smell. This natural α,β-unsaturated aldehyde was isolated for the first time by Dumas and Péligot in 1834 and synthesized by Chiozza in 1854 [15,16]. Cinnamaldehyde does not violate the Lipinski rules, and, thus, it is a promising drug candidate for the treatment of various human ailments. For instance, cinnamaldehyde exhibited pronounced activities against fungal, protozoal, and worm infections [17,18,19,20,21,22]. The strong antioxidant properties of cinnamaldehyde protect cells from reactive oxygen species (ROS)-mediated damage, and cinnamaldehyde even showed convincing effects as an eco-friendly steel corrosion inhibitor [23,24]. The synthetic cinnamaldehyde derivative 4-dimethylaminocinnamaldehyde (DMACA) is of relevance as a dye for the detection of indoles, proanthocyanidins, and flavonoids in various biological materials and products (Figure 1) [25,26,27]. An acyl hydrazone derivative of DMACA was developed as a Pb(II) and biothiol detector [28]. Analogous acyl hydrazones made from cinnamaldehyde showed antimalarial and antibiotic activities, which underlines the anti-infective potential of semi-synthetic cinnamaldehyde derivatives [29,30]. Cinnamaldehyde phenyl hydrazone (CPH) exhibited moderate to weak antifungal and anti-inflammatory activities (Figure 1) [31].

Frequent and long-term consumption of cinnamaldehyde and cinnamon-containing products can cause adverse effects such as intraoral inflammations and allergic reactions [32]. Thus, less toxic alternatives, which can conserve the beneficial biological activities of cinnamaldehyde, are also of interest. Modern synthetic and structurally related fragrances, such as the floral odorants silvial^TM^, viz., 2-methyl-3-[4-(2-methylpropyl)phenyl]propanal, and florhydral^TM^, viz., 3-(3-isopropylphenyl)butanal, lack the reactive/alkylating α,β-unsaturated carbonyl system of cinnamaldehyde (Figure 1). Notably, the metabolism of silvial^TM^, 2-methyl-3-[4-(2-methylpropyl)phenyl]propanal, into 4-*tert*-butylbenzoic acid was associated with spermatotoxic effects in rats by formation of conjugates with CoA and changes in the cellular metabolome [33]. An enantiopure synthesis of (+)- and (−)-silvial^TM^ was achieved in four steps from 4-*tert*-butylbenzaldehyde including a catalytic asymmetric hydrogenation reaction with an axially chiral ruthenium-phosphine catalyst [34]. Unlike other fragrances, florhydral^TM^, 3-(3-isopropylphenyl)butanal, formed matrix-type polymeric capsules with the amphiphilic graft copolymer Soluplus in aqueous solutions upon a self-assembly process [35]. Enantioselective syntheses of (+)- and (−)-florhydral^TM^ were accomplished using enzymes (lipases) or a chiral iridium-phosphinooxazoline hydrogenation catalyst [36,37]. The synthetic approach to fragrances paves the way for the preparation of new derivatives with multiple biological activities including anti-infective properties.

**Scheme 1:**
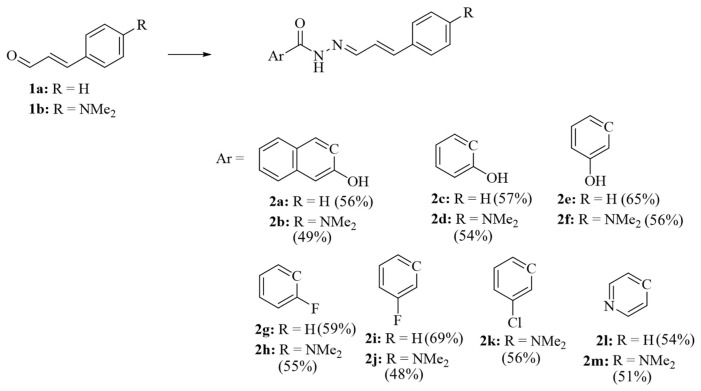
Reagents and conditions: aroyl hydrazide (1.0 equiv.), EtOH, reflux, 2 h, yields in brackets.

**Scheme 2:**
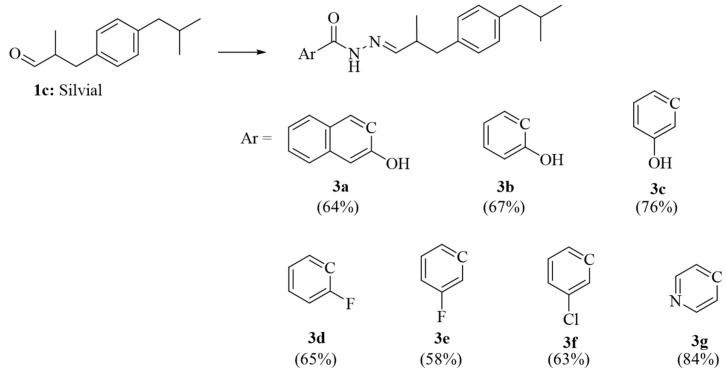
Reagents and conditions: aroyl hydrazide (1.0 equiv.), EtOH, reflux, 2 h, yields in brackets.

**Scheme 3:**
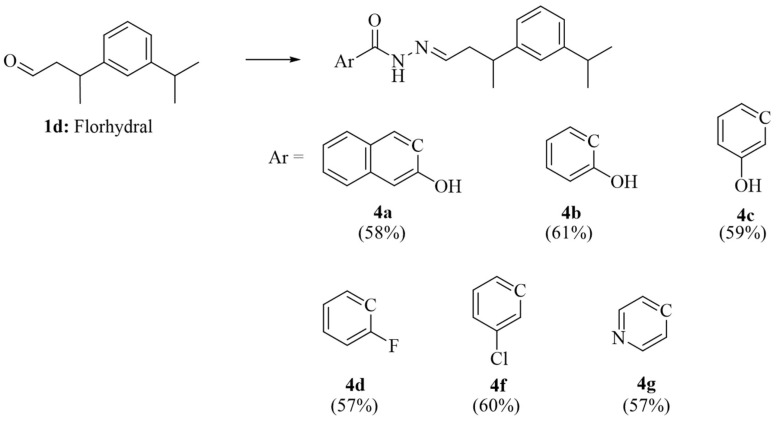
Reagents and conditions: aroyl hydrazide (1.0 equiv.), EtOH, reflux, 2 h, yields in brackets.

Synthetic antiparasitic lead compounds were successfully prepared upon the identification of the antiplasmodial properties of marine natural products such as aplidinones [38]. Thus, cinnamaldehyde and structurally related aldehydes might also serve as reasonable templates for the design of potent antiparasitic derivatives. The aldehyde functions of cinnamaldehydes and of the related fragrances silvial^TM^ and florhydral^TM^ are suitable for chemical modifications. Acyl hydrazones of cinnamaldehydes seem to be promising candidates for antiparasitic testing [29]. Our groups recently disclosed acyl hydrazones with nitrofuran and nitrothiophene moieties that exhibited high activities against *T. gondii* and *L. major* parasites [39]. In continuation of these efforts, we now present the promising activities of acyl hydrazones made from cinnamaldehyde, DMACA, silvial^TM^, and florhydral^TM^ against *T. gondii* and *L. major* (Figure 1). Various hydroxy-substituted and halogen-substituted aroyl hydrazides were applied as starting compounds, as well as the anti-tubercular drug isoniazid (Scheme 1, Scheme 2 and Scheme 3). 

## 2. Results

### 2.1. Chemistry

Cinnamaldehyde (**1a**) and DMACA (**1b**) were converted to the acyl hydrazones **2a**–**m** by reaction with 1.0 equivalent of the indicated aroyl hydrazide in hot ethanol for 2 h (Scheme 1). The hydrazone products were obtained as solids in moderate yields. Compounds **2a**–**c**, **2g**, **2l**, and **2m** are known, while compounds **2d**–**f** and **2h**–**k** are new [28,29,30,40,41,42,43]. 

Analogous acyl hydrazones were prepared from the commercial fragrances silvial^TM^ (**3a**–**g**) and florhydral^TM^ (**4a**–**g**) via the same method (Scheme 2 and Scheme 3). Except for **3e**, all hydrazones of silvial^TM^ were solids. In contrast, except for solids **4a** and **4c**, compounds **4** derived from florhydral^TM^ were obtained as colorless oils or gums. The yields of silvial^TM^ derivatives **3** were generally higher than the yields of florhydral^TM^ derivatives **4**. 

### 2.2. Antiparasitic Activity

The activities of the tested compounds against *T. gondii* parasites were initially evaluated and compared with their toxicity to Vero kidney cells (Table 1). The starting compounds cinnamaldehyde, silvial^TM^, and florhydral^TM^, as well as CPH and the approved toxoplasmosis drug atovaquone, were used as control compounds. The cinnamaldehyde-derived acyl hydrazones **2a** and **2c**, derived from 3-hydroxy-2-naphthoic hydrazide and 2-hydroxybenzoyl hydrazide, respectively, were the most active compounds against *T. gondii*, (IC_50_ = 0.63 µM and 1.1 µM, respectively). These compounds were also the only ones that were distinctly less toxic to Vero cells and exhibited considerable selectivity for *T. gondii* and SI values of 11.5 and 17.3, respectively. Of note, cinnamaldehyde was inactive against *T. gondii*, while its phenyl hydrazone CPH showed only moderate activity (IC_50_ = 12.9 µM). Thus, **2a** and **2c** were also much more antitoxoplasmal than these reference compounds, and more selective than CPH (SI = 1.8), but they were less active than the positive control drug atovaquone [44]. The DMACA-derived compound **2d** also showed reasonable activity against *T. gondii* being much more active than its close analog **2b**, but staying below the activity and selectivity level of **2a** and **2c**. In addition, **2c** and **2d** were less toxic to Vero cells than atovaquone. Compounds **2e**–**m** were only weakly active or inactive. In particular, the 3-hydroxybenzoyls **2e** and **2f**, which are close analogs and isomers of **2c** and **2d**, were inactive against *T. gondii*. Halogen (fluoro- or chloro-) substitution of the aroyl ring of compounds **2g**–**k**, as well as isonicotinoyl moieties (**2l** and **2m**) led to reduced activity. 

None of the acyl hydrazones derived from silvial^TM^ (**3a**–**g**) and florhydral^TM^ (**4a**–**d**, **4f**, **4g**) reached activities below 10 µM. 2-Hydroxybenzoyl hydrazone **3b** and 3-hydroxy-2-naphthoic hydrazone **4a** were the most active derivatives of these fragrances with moderate effects (IC_50_ = 13.7 µM and 14.4 µM, respectively), followed by 2-hydroxybenzoyl hydrazone **4b** and 3-chlorobenzoyl hydrazone **3f** (IC_50_ = 21.0 µM and 22.0 µM, respectively). Compound **3f** was distinctly more active than its 3-chlorobenzoyl analogs **2k** (from DMACA) and **4f** (from florhydral^TM^). Both of the fragrances silvial^TM^ and florhydral^TM^ were only weakly active (IC_50_ = 90.1 µM for silvial^TM^) or inactive (IC_50_ > 105.1 µM for florhydral^TM^) against *T. gondii*. Albeit only weakly active, silvial^TM^ was more active than cinnamaldehyde, which indicates that synthetic fragrances can surpass the activity of structurally related natural products. 

The activities of the test compounds against *L. major* promastigotes and amastigotes were also investigated and compared with their toxicity to macrophages (Table 2). Only the 3-hydroxy-2-naphthoic compounds **2a** and **4a** showed moderate activity against promastigotes. Of note, florhydral^TM^-derived **4a** (IC_50_ = 9.6 µM) was slightly more active than cinnamaldehyde-derived **2a** (IC_50_ = 16.8 µM), followed by the 3-chlorobenzoyl hydrazones **4f** (IC_50_ = 27.9 µM) and **3f** (IC_50_ = 29.1 µM). All compounds were inactive against amastigotes and, most notably, against macrophages. Thus, **4a** has a promastigote-correlated SI value of min. 5.6 indicating at least moderate selectivity of this compound for *L. major* parasites. However, **4a** was distinctly less active than the positive control drug amphotericin B. CPH was only weakly active against *L. major* promastigotes (IC_50_ = 83.7 µM), while cinnamaldehyde, silvial^TM^, and florhydral^TM^ were inactive in these experiments.

## 3. Discussion

Plant-derived fragrances have distinct pharmaceutical potential and their odors are well-documented stress reducers [45,46,47]. Approximately 190 years after the first isolation of cinnamaldehyde, promising antiparasitic acyl hydrazones of cinnamaldehyde and structurally related fragrances were identified from tests against *T. gondii* and *L. major* parasites in this study. Sound activities against *T. gondii* were observed for two cinnamaldehyde hydrazones with 3-hydroxy-2-naphthoic hydrazide (**2a**) and 2-hydroxybenzoyl hydrazide (**2c**), which also exhibited considerable selectivities (SI = 11.5 and 17.3), since SI values above 10 are acceptable for new antiparasitic hit compounds [48]. In contrast, the non-modified starting fragrance compounds were inactive in our tests which underlines the necessity of their modification to achieve drug candidates with considerable antiparasitic effects. Chemical optimization and fine-tuning might improve the shown antitoxoplasmal activities. These efforts might include the application of further ring-substituted cinnamaldehyde starting compounds, the preparation of metal complexes (acyl hydrazones are excellent metal chelators), and the replacement of the hydrazone bridge by hydrolytically more stable groups such as ureas and amides. For instance, we previously showed that Co(II) and Cu(II) complexes of acyl hydrazones with 5-nitrofurans conserved the high activity of the hydrazone ligand against *T. gondii* [39]. Of note, the most active compounds are cinnamaldehyde-derived 3-hydroxy-2-naphthoic and 2-hydroxybenzoyl hydrazones. Replacement of the OH-group by halogens and changes of its position at the aromatic ring strongly reduced activity. Only the silvial^TM^- and florhydral^TM^-derived 3-chlorobenzoyl hydrazones **3f** and **4f** showed activities at concentrations below 30 µM. The performance of the fluoro-substituted derivatives was only low. In contrast, the 3-hydroxy-2-naphthoic moiety appeared to be especially favored and also exerted antileishmanial activity at doses below 10 µM in conjugation with florhydral^TM^ (**4a**), which lacks the olefin moiety of the cinnamaldehyde derivatives. This is remarkable since *L. major* cells were shown to be quite insensitive to hydrazones of this series, except for **4a** and its cinnamaldehyde-based analog **2a**. 

Yet, the high antitoxoplasmal activities of **2a** and **2c** accompanied by relatively low toxicities to host renal cells are the most promising outcomes of this study. Although less active than the approved drug atovaquone, **2a** and **2c** might be beneficial under certain circumstances. Apicomplexan parasites such as *T. gondii* and *Plasmodium falciparum* developed atovaquone resistance, which hampers treatment outcome [6,49]. In addition, failure of *T. gondii* infection prophylaxis was reported for atovaquone therapy in some transplant patients [50]. Thus, new active drugs are required and the identified antitoxoplasmal cinnamaldehyde hydrazones might lead to the development of potent and cost-effective drug candidates for toxoplasmosis therapy and prophylaxis. 

Several other hydrazones made from 3-hydroxy-2-naphthoyl hydrazide were reportedly active against parasites and viruses [39,51]. The endocytosis inhibitor dynasore, which targets the GTPase dynamin, is another prominent catechol-based example [52]. Thus, the analogous cinnamaldehyde and florhydral^TM^ hydrazones **2a** and **4a** presented in this study add well to the already existing data. Whether dynamin is a target of these compounds, too, remains to be shown. Nevertheless, dynamin is a vital factor in *T. gondii* parasite cells and might be a reasonable target for antiparasitic drugs [53,54,55]. Oxidative processes and associated mechanisms might also play a role either in toxicity to parasite cells or in renal cell protection. Notably, a cell-protective correlation between cinnamaldehyde-mediated upregulation of the oxidative sensor TRPA1 and suppression of dynamin-related protein 1 (Drp1) was recently found in renal cells [56]. 

## 4. Materials and Methods

### 4.1. Chemistry

Starting compounds were purchased from Merck (Darmstadt, Germany), Alfa Aesar (Karlsruhe, Germany), and TCI (Zwijndrecht, Belgium). The fragrances silvial^TM^ and florhydral^TM^ were obtained from Givaudan (Vernier, Switzerland). Isoniazid was prepared from ethyl isonicotinate and hydrazine hydrate according to the procedure in the literature [57]. Cinnamaldehyde phenylhydrazone (CPH) was prepared according to a reported protocol [31]. The known compounds **2a**–**c**, **2g**, **2l**, and **2m** were synthesized according to the literature [28,29,30,40,41,42,43]. Their ^1^H NMR data can be found in the Supplementary Materials.

#### 4.1.1. (*E*)-*N*′-(4-Dimethylaminocinnamylidene)-2-hydroxybenzoylhydrazide (**2d**)—Typical Procedure for the Synthesis of Compound **2**

2-Hydroxybenzoyl hydrazide (152 mg, 1.0 mmol) and 4-dimethylaminocinnamaldehyde (175 mg, 1.0 mmol) were dissolved in EtOH (10 mL). The reaction mixture was stirred under reflux (78 °C) for 2 h. The formed precipitate was collected, washed with EtOH (10 mL), and dried in a vacuum. Yield: 166 mg (0.54 mmol, 54%); yellow solid of m.p., 208 °C; *υ*_max_(ATR)/cm^−1^ 3258, 3069, 3031, 2894, 2857, 2814, 2726, 2584, 1647, 1601, 1579, 1544, 1521, 1502, 1491, 1454, 1443, 1361, 1335, 1305, 1236, 1215, 1180, 1167, 1149, 1096, 1066, 1004, 986, 948, 910, 854, 824, 804, 781, 745, 729, and 659; ^1^H NMR (300 MHz, DMSO-*d*_6_) *δ* 2.95 (6 H, s), 6.71 (2 H, d, J = 8.9 Hz), 6.8–7.0 (4 H, m), 7.4–7.5 (3 H, m), 7.8–7.9 (1 H, m), 8.19 (1 H, d, J = 9.1 Hz), 11.62 (1 H, s), and 12.0–12.1 (1 H, br s); ^13^C NMR (75.5 MHz, DMSO-*d*_6_) *δ* 112.0, 115.6, 117.3, 118.8, 120.2, 123.5, 128.2, 128.5, 133.7, 140.7, 150.8, 151.8, and 159.4, 164.5; HR-MS (ESI, *m/z*) for C_18_H_20_O_2_N_3_ [M + H^+^] calcd. 310.15500, found 310.15478.

#### 4.1.2. (*E*)-*N*′-(Cinnamylidene)-3-hydroxybenzoylhydrazide (**2e**)

Analogously to the synthesis of **2d**, compound **2e** was obtained from 3-hydroxybenzoyl hydrazide (152 mg, 1.0 mmol) and cinnamaldehyde (132 mg, 1.0 mmol). Yield: 173 mg (0.65 mmol, 65%); colorless solid of m.p., 249–250 °C; *υ*_max_(ATR)/cm^−1^ 3241, 1634, 1623, 1587, 1536, 1451, 1363, 1299, 1242, 1128, 1054, 996, 983, 960, 872, 855, 756, 693, and 682; ^1^H NMR (300 MHz, DMSO-*d*_6_) *δ* 2.95 (6 H, s), 6.71 (2 H, d, J = 8.9 Hz), 6.8–7.0 (3 H, m), 7.2–7.3 (3 H, m), 7.44 (2 H, d, J = 8.9 Hz), 8.16 (1 H, d, J = 8.9 Hz), 9.74 (1 H, s), and 11.50 (1 H, s); ^13^C NMR (75.5 MHz, DMSO-*d*_6_) *δ* 114.5, 118.1, 118.7, 125.7, 127.1, 128.8, 129.5, 134.8, 135.9, 138.9, 149.6, 157.4, and 163.0; HR-MS (ESI, *m/z*) for C_16_H_15_O_2_N_2_ [M + H^+^] calcd. 267.11280, found 267.11321.

#### 4.1.3. (*E*)-*N*′-(4-Dimethylaminocinnamylidene)-3-hydroxybenzoylhydrazide (**2f**)

Analogously to the synthesis of **2d**, compound **2f** was obtained from 3-hydroxybenzoyl hydrazide (152 mg, 1.0 mmol) and 4-dimethylaminocinnamaldehyde (175 mg, 1.0 mmol). Yield: 172 mg (0.56 mmol, 56%); yellow solid of m.p., 257–258 °C; *υ*_max_(ATR)/cm^−1^ 3265, 3229, 3066, 3034, 2895, 2811, 1606, 1578, 1538, 1446, 1362, 1317, 1298, 1239, 1190, 1170, 1132, 1051, 998, 980, 960, 869, 858, 831, 806, and 681; ^1^H NMR (300 MHz, DMSO-*d*_6_) *δ* 2.95 (6 H, s), 6.71 (2 H, d, J = 8.9 Hz), 6.8–7.0 (3 H, m), 7.2–7.3 (3 H, m), 7.44 (2 H, d, J = 8.9 Hz), 8.16 (1 H, d, J = 8.9 Hz), 9.74 (1 H, s), and 11.50 (1 H, s); ^13^C NMR (75.5 MHz, DMSO-*d*_6_) *δ* 112.0, 114.4, 118.0, 118.5, 120.6, 123.7, 128.4, 129.5, 135.1, 139.8, 150.6, 150.7, 157.4, and 162.7; HR-MS (ESI, *m/z*) for C_18_H_20_O_2_N_3_ [M + H^+^] calcd. 310.15500, found 310.15494.

#### 4.1.4. (*E*)-*N*′-(4-Dimethylaminocinnamylidene)-2-fluorobenzoylhydrazide (**2h**)

Analogously to the synthesis of **2d**, compound **2h** was obtained from 2-fluorobenzoic hydrazide (154 mg, 1.0 mmol) and 4-dimethylaminocinnamaldehyde (175 mg, 1.0 mmol). Yield: 170 mg (0.55 mmol, 55%); yellow solid of m.p., 205–206 °C; *υ*_max_(ATR)/cm^−1^ 3196, 3062, 2849, 2802, 1645, 1600, 1575, 1545, 1520, 1480, 1451, 1356, 1322, 1300, 1252, 1216, 1190, 1162, 1140, 1097, 1058, 984, 970, 945, 932, 905, 872, 864, 809, 778, 754, 722, 683, and 656; ^1^H NMR (300 MHz, DMSO-*d*_6_) *δ* 2.95 (6 H, s), 6.6–6.9 (5 H, m), 7.3–7.4 (3 H, m), 7.44 (2 H, d, J = 8.9 Hz), 7.6–7.7 (2 H, m), 8.03 (1 H, d, J = 9.1 Hz), and 11.56 (1 H, s); ^13^C NMR (75.5 MHz, DMSO-*d*_6_) *δ* 111.9, 116.0–116.2 (m), 120.2, 123.4, 124.6, 128.4, 129.3, 130.1, 132.6, 140.4, 148.0, 150.6–150.8 (m), 157.4–160.7 (m), 159.9, and 166.5; HR-MS (ESI, *m/z*) for C_18_H_19_ON_3_F [M + H^+^] calcd. 312.15067, found 312.15019.

#### 4.1.5. (*E*)-*N*′-(Cinnamylidene)-3-fluorobenzoylhydrazide (**2i**)

Analogously to the synthesis of **2d**, compound **2i** was obtained from 3-fluorobenzoyl hydrazide (154 mg, 1.0 mmol) and cinnamaldehyde (132 mg, 1.0 mmol). Yield: 186 mg (0.69 mmol, 69%); colorless solid of m.p., 205–206 °C; *υ*_max_(ATR)/cm^−1^ 3233, 3061, 1650, 1627, 1588, 1580, 1539, 1483, 1450, 1440, 1360, 1333, 1317, 1287, 1273, 1220, 1210, 1182, 1165, 1116, 1074, 1058, 999, 977, 949, 897, 881, 846, 816, 791, 747, 690, 678, and 655; ^1^H NMR (300 MHz, DMSO-*d*_6_) *δ* 6.9–7.0 (1 H, m), 7.0–7.1 (2 H, m), 7.2–7.4 (6 H, m), 7.6–7.7 (2 H, m), 8.2–8.3 (1 H, m), 9.77 (1 H, s), and 11.67 (1 H, s); ^13^C NMR (75.5 MHz, DMSO-*d*_6_) *δ* 114.3–114.6 (m), 118.5–118.8 (m), 123.8, 125.5, 127.2, 128.9, 130.7, 135.7–135.9 (m), 139.4, 150.3, 160.3–163.5 (m), and 161.6; HR-MS (ESI, *m/z*) for C_16_H_14_ON_2_F [M + H^+^] calcd. 269.10847, found 269.10871.

#### 4.1.6. (*E*)-*N*′-(4-Dimethylaminocinnamylidene)-3-fluorobenzoylhydrazide (**2j**)

Analogously to the synthesis of **2d**, compound **2j** was obtained from 3-fluorobenzoyl hydrazide (154 mg, 1.0 mmol) and 4-dimethylaminocinnamaldehyde (175 mg, 1.0 mmol). Yield: 149 mg (0.48 mmol, 48%); yellow solid of m.p., 208 °C; *υ*_max_(ATR)/cm^−1^ 3234, 3059, 3027, 2891, 2853, 2808, 1649, 1603, 1576, 1538, 1523, 1483, 1439, 1360, 1322, 1288, 1269, 1219, 1188, 1163, 1111, 1051, 982, 946, 892, 858, 804, 795, 747, and 678; ^1^H NMR (300 MHz, DMSO-*d*_6_) *δ* 2.95 (6 H, s), 6.71 (2 H, d, J = 8.9 Hz), 6.8–7.0 (3 H, m), 7.45 (2 H, d, J = 8.9 Hz), 7.5–7.6 (1 H, m), 7.7–7.8 (2 H, m), 8.17 (1 H, d, J = 9.1 Hz), and 11.64 (1 H, s); ^13^C NMR (75.5 MHz, DMSO-*d*_6_) *δ* 112.0, 114.2–114.5 (m), 118.3–118.6 (m), 120.3, 123.6–123.8 (m), 128.5, 130.6, 135.9, 140.2, 150.7, 151.2, 160.3–163.5 (m), and 161.3; HR-MS (ESI, *m/z*) for C_18_H_19_ON_3_F [M + H^+^] calcd. 312.15067, found 312.15020.

#### 4.1.7. (*E*)-*N*′-(4-Dimethylaminocinnamylidene)-3-chlorobenzoylhydrazide (**2k**)

Analogously to the synthesis of **2d**, compound **2k** was obtained from 3-chlorobenzoyl hydrazide (171mg, 1.0 mmol) and 4-dimethylaminocinnamaldehyde (175 mg, 1.0 mmol). Yield: 185 mg (0.56 mmol, 56%); yellow solid of m.p., 210 °C; *υ*_max_(ATR)/cm^−1^ 3208, 3047, 2888, 2850, 2801, 1648, 1638, 1619, 1603, 1573, 1542, 1521, 1475, 1443, 1425, 1355, 1325, 1313, 1286, 1270, 1227, 1192, 1165, 1144, 1088, 1058, 1002, 983, 943, 924, 879, 807, 747, 718, 678, and 653; ^1^H NMR (300 MHz, DMSO-*d*_6_) *δ* 2.96 (6 H, s), 6.71 (2 H, d, J = 8.9 Hz), 6.8–7.0 (3 H, m), 7.44 (2 H, d, J = 8.9 Hz), 7.5–7.7 (2 H, m), 7.8–7.9 (1 H, m), 7.93 (1 H, s), 8.16 (1 H, d, J = 9.1 Hz), and 11.67 (1 H, s); ^13^C NMR (75.5 MHz, DMSO-*d*_6_) *δ* 112.0, 120.3, 123.6, 126.4, 127.2, 128.5, 130.5, 131.4, 133.2, 135.6, 140.3, 150.8, 151.3, and 161.2; HR-MS (ESI, *m/z*) for C_18_H_19_ON_3_Cl [M + H^+^] calcd. 328.12112, found 328.12088.

#### 4.1.8. *N*′-2-Methyl-3-[4-(2-methylpropyl)phenyl]propanylidene-3-hydroxy-2-naphthoic hydrazide (**3a**)—Typical Procedure for the Synthesis of Compound **3**

3-Hydroxy-2-naphthoic hydrazide (202 mg, 1.0 mmol) and silvial^TM^ (204 mg, 1.0 mmol) were dissolved in EtOH (10 mL). The reaction mixture was stirred under reflux (78 °C) for 2 h. The formed precipitate was collected, washed with EtOH (10 mL), and dried in a vacuum. For the synthesis of compounds **3c**–**g**, the solvent was evaporated and the residue was recrystallized from EtOH (10 mL, for **3c**, **3d**, and **3f**) or washed with *n*-hexane (10 mL, for **3e** and **3g**). Yield: 262 mg (0.68 mmol, 68%); colorless solid of m.p., 164–165 °C; *υ*_max_(ATR)/cm^−1^ 3230, 3054, 2951, 2925, 2866, 1651, 1616, 1583, 1556, 1508, 1463, 1448, 1418, 1380, 1366, 1346, 1314, 1287, 1228, 1170, 1141, 1116, 1097, 1077, 1055, 1027, 1016, 954, 919, 903, 877, 853, 835, 812, 799, 780, 764, 754, and 743; ^1^H NMR (300 MHz, CDCl_3_) *δ* 0.87 (6 H, d, J = 6.6 Hz), 1.15 (3 H, d, J = 6.7 Hz), 1.8–1.9 (1 H, m), 2.41 (2 H, d, J = 7.2 Hz), 2.6–2.7 (1 H, m), 2.8–2.9 (2 H, m), 7.0–7.1 (4 H, m), 7.3–7.4 (2 H, m), 7.4–7.5 (2 H, m), 7.6–7.7 (2 H, m), 8.0–8.1 (1 H, br s), 9.2–9.3 (1 H, br s), and 11.0–11.2 (1 H, br s); ^13^C NMR (75.5 MHz, CDCl_3_) *δ* 17.3, 22.4, 30.2, 38.3, 40.3, 45.0, 112.7, 115.9, 124.1, 126.3, 128.3, 128.9, 129.2, 136.0, 137.2, 139.8, and 156.6; HR-MS (ESI, *m/z*) for C_25_H_29_O_2_N_2_ [M + H^+^] calcd. 389.22235, found 389.22189.

#### 4.1.9. *N*′-2-Methyl-3-[4-(2-methylpropyl)phenyl]propanylidene-2-hydroxybenzoylhydrazide (**3b**)

Analogously to the synthesis of **3a**, compound **3b** was obtained from 2-hydroxybenzoic hydrazide (152 mg, 1.0 mmol) and silvial^TM^ (204 mg, 1.0 mmol). Yield: 225 mg (0.67 mmol, 67%); colorless solid of m.p., 160–161 °C; *υ*_max_(ATR)/cm^−1^ 3257, 3023, 2954, 2922, 2867, 2843, 2704, 2580, 1656, 1627, 1602, 1581, 1550, 1491, 1454, 1362, 1309, 1234, 1213, 1158, 1115, 1099, 1051, 956, 923, 901, 885, 855, 833, 815, 788, and 753; ^1^H NMR (300 MHz, DMSO-*d*_6_) *δ* 0.87 (6 H, d, J = 6.6 Hz), 1.12 (3 H, d, J = 6.7 Hz), 1.8–1.9 (1 H, m), 2.41 (2 H, d, J = 7.2 Hz), 2.4–2.5 (1 H, m), 2.8–2.9 (2 H, m), 6.8–6.9 (1 H, m), 7.0–7.1 (5 H, m), 7.3–7.4 (3 H, m), 9.0–9.1 (1 H, br s), and 11.8–11.9 (1 H, br s); ^13^C NMR (75.5 MHz, DMSO-*d*_6_) *δ* 17.3, 22.4, 30.2, 38.3, 40.3, 45.0, 113.0, 118.3, 118.8, 125.1, 128.3, 128.9, 129.2, 130.1, 134.8, 136.0, 139.8, 156.9, and 162.0; HR-MS (ESI, *m/z*) for C_21_H_27_O_2_N_2_ [M + H^+^] calcd. 339.20670, found 339.20609.

#### 4.1.10. *N*′-2-Methyl-3-[4-(2-methylpropyl)phenyl]propanylidene-3-hydroxybenzoylhydrazide (**3c**)

Analogously to the synthesis of **3a**, compound **3c** was obtained from 3-hydroxybenzoic hydrazide (152 mg, 1.0 mmol) and silvial^TM^ (204 mg, 1.0 mmol). Yield: 258 mg (0.76 mmol, 76%); colorless solid of m.p., 182–183 °C; *υ*_max_(ATR)/cm^−1^ 3221, 3050, 2965, 2922, 2866, 1649, 1630, 1599, 1541, 1513, 1446, 1383, 1356, 1298, 1246, 1232, 1167, 1131, 1069, 1048, 1021, 998, 935, 889, 872, 830, 799, 758, 687, and 661; ^1^H NMR (300 MHz, DMSO-*d*_6_) *δ* 0.84 (6 H, d, J = 6.6 Hz), 1.01 (3 H, d, J = 6.9 Hz), 1.7–1.8 (1 H, m), 2.40 (2 H, d, J = 7.1 Hz), 2.5–2.8 (3 H, m), 6.9–7.0 (1 H, m), 7.0–7.2 (4 H, m), 7.2–7.3 (3 H, m), 7.7–7.8 (1 H, m), 9.70 (1 H, s), and 11.32 (1 H, s); ^13^C NMR (75.5 MHz, DMSO-*d*_6_) *δ* 17.2, 22.2, 29.6, 38.0, 44.3, 114.4, 117.9, 118.4, 128.8, 128.9, 129.4, 135.0, 136.7, 138.7, 155.4, 157.3, and 162.8; HR-MS (ESI, *m/z*) for C_21_H_27_O_2_N_2_ [M + H^+^] calcd. 339.20670, found 339.20632.

#### 4.1.11. *N*′-2-Methyl-3-[4-(2-methylpropyl)phenyl]propanylidene-2-fluorobenzoylhydrazide (**3d**)

Analogously to the synthesis of **3a**, compound **3d** was obtained from 2-fluorobenzoic hydrazide (154 mg, 1.0 mmol) and silvial^TM^ (204 mg, 1.0 mmol). Yield: 220 mg (0.65 mmol, 65%); colorless solid of m.p., 82–83 °C; *υ*_max_(ATR)/cm^−1^ 3245, 3069, 2957, 2924, 2869, 1653, 1623, 1614, 1543, 1514, 1482, 1454, 1418, 1366, 1299, 1252, 1217, 1168, 1137, 1100, 1057, 1037, 1026, 968, 892, 860, 833, 816, 782, 755, 708, and 655; ^1^H NMR (300 MHz, CDCl_3_) *δ* 0.87 (6 H, d, J = 6.6 Hz), 1.11 (3 H, d, J = 6.6 Hz), 1.7–1.9 (1 H, m), 2.41 (2 H, d, J = 7.2 Hz), 2.6–2.7 (1 H, m), 2.8–3.0 (2 H, m), 7.0–7.1 (5 H, m), 7.2–7.3 (1 H, m), 7.4–7.5 (2 H, m), 8.1–8.2 (2 H, m), and 9.3–9.4 (1 H, m); ^13^C NMR (75.5 MHz, CDCl_3_) *δ* 17.2, 22.2, 30.2, 38.3, 40.2, 45.0, 115.8–116.1 (m), 119.8–120.0 (m), 125.1, 128.5, 128.9, 129.0, 129.1, 129.5, 132.5, 133.7, 136.1, 139.6, 154.7, 156.6, 158.6–161.8 (m), and 159.7; HR-MS (ESI, *m/z*) for HR-MS (ESI, *m/z*) for C_21_H_26_ON_2_F [M + H^+^] calcd. 341.20237, found 341.20180.

#### 4.1.12. *N*′-2-Methyl-3-[4-(2-methylpropyl)phenyl]propanylidene-3-fluorobenzoylhydrazide (**3e**)

Analogously to the synthesis of **3a**, compound **3e** was obtained from 3-fluorobenzoic hydrazide (50 mg, 0.33 mmol) and silvial^TM^ (66 mg, 0.33 mmol). Yield: 63 mg (0.19 mmol, 58%); colorless oil; *υ*_max_(ATR)/cm^−1^ 3212, 3071, 2958, 2925, 2870, 2847, 1650, 1586, 1559, 1515, 1486, 1441, 1362, 1315, 1294, 1271, 1223, 1170, 1119, 1074, 1041, 1003, 973, 851, 935, 891, 844, 835, 816, 798, 747, and 679; ^1^H NMR (300 MHz, CDCl_3_) *δ* 0.85 (6 H, d, J = 6.6 Hz), 1.01 (3 H, d, J = 6.2 Hz), 1.7–1.9 (1 H, m), 2.37 (2 H, d, J = 7.1 Hz), 2.5–2.6 (1 H, m), 2.7–2.9 (2 H, m), 7.0–7.1 (4 H, m), 7.1–7.2 (1 H, m), 7.2–7.3 (1 H, m), 7.5–7.7 (3 H, m), and 10.1–10.2 (1 H, br s); ^13^C NMR (75.5 MHz, CDCl_3_) *δ* 17.1, 22.3, 30.1, 38.3, 40.2, 45.0, 114.6–114.9 (m), 118.6–118.9 (m), 123.0, 128.3, 128.8, 129.1, 130.1–130.4 (m), 136.1, 139.5, 157.2, 160.9–164.2 (m), and 163.1; HR-MS (ESI, *m/z*) for C_21_H_26_ON_2_F [M + H^+^] calcd. 341.20237, found 341.20169.

#### 4.1.13. *N*′-2-Methyl-3-[4-(2-methylpropyl)phenyl]propanylidene-3-chlorobenzoylhydrazide (**3f**)

Analogously to the synthesis of **3a**, compound **3f** was obtained from 3-chlorobenzoic hydrazide (171 mg, 1.0 mmol) and silvial^TM^ (204 mg, 1.0 mmol). Yield: 223 mg (0.63 mmol, 63%); colorless solid of m.p., 88–89 °C; *υ*_max_(ATR)/cm^−1^ 3221, 3066, 2957, 2924, 2868, 2847, 1651, 1558, 1514, 1471, 1421, 1359, 1312, 1286, 1265, 1202, 1167, 1121, 1093, 1068, 1045, 998, 940, 922, 900, 866, 833, 801, 747, 737, and 677; ^1^H NMR (300 MHz, CDCl_3_) *δ* 0.86 (6 H, d, J = 6.6 Hz), 1.10 (3 H, d, J = 6.4 Hz), 1.7–1.9 (1 H, m), 2.41 (2 H, d, J = 7.2 Hz), 2.6–2.7 (1 H, m), 2.8–2.9 (2 H, m), 7.0–7.1 (4 H, m), 7.3–7.5 (3 H, m), 7.6–7.7 (1 H, m), 7.7–7.8 (1 H, m), and 8.8–8.9 (1 H, m); ^13^C NMR (75.5 MHz, CDCl_3_) *δ* 17.3, 22.4, 30.2, 38.2, 40.3, 45.0, 124.8, 125.3, 127.5, 128.3, 128.9, 129.2, 130.1, 132.0, 136.1, 139.7, 156.7, and 162.5; HR-MS (ESI, *m/z*) for C_21_H_26_ON_2_Cl [M + H^+^] calcd. 357.17282, found 357.17227.

#### 4.1.14. *N*′-2-Methyl-3-[4-(2-methylpropyl)phenyl]propanylidene-isonicotinoylhydrazide (**3g**)

Analogously to the synthesis of **3a**, compound **3g** was obtained from isoniazid (138 mg, 1.0 mmol) and silvial^TM^ (204 mg, 1.0 mmol). Yield: 271 mg (0.84 mmol, 84%); colorless solid of m.p., 122–123 °C; *υ*_max_(ATR)/cm^−1^ 3222, 3051, 2956, 2924, 2869, 1653, 1634, 1598, 1550, 1514, 1493, 1453, 1409, 1363, 1331, 1314, 1294, 1216, 1167, 1137, 1094, 1067, 1040, 992, 940, 915, 877, 860, 844, 811, 753, 707, and 680; ^1^H NMR (300 MHz, CDCl_3_) *δ* 0.8–0.9 (6 H, m), 1.05 (3 H, d, J = 6.3 Hz), 1.7–1.9 (1 H, m), 2.38 (2 H, d, J = 7.2 Hz), 2.6–2.9 (3 H, m), 7.0–7.1 (4 H, m), 7.5–7.6 (2 H, m), 8.6–8.7 (2 H, m), and 10.01 (1 H, s); ^13^C NMR (75.5 MHz, CDCl_3_) *δ* 17.2, 22.3, 30.2, 38.4, 40.2, 45.0, 121.2, 123.6, 128.8, 129.1, 135.9, 139.7, 140.5, 149.5, 150.4, 152.9, 158.1, and 162.3; HR-MS (ESI, *m/z*) for C_20_H_26_ON_3_ [M + H^+^] calcd. 324.20704, found 324.20672.

#### 4.1.15. *N*′-3-(3-Isopropylphenyl)butanylidene-3-hydroxy-2-naphthoic hydrazide (**4a**)—Typical Procedure for the Synthesis of Compound **4**

3-Hydroxy-2-naphthoic hydrazide (202 mg, 1.0 mmol) and florhydral^TM^ (190 mg, 1.0 mmol) were dissolved in EtOH (10 mL). The reaction mixture was stirred under reflux (78 °C) for 2 h. After the reaction, the solvent was evaporated and the residue was crystallized from CH_2_Cl_2_/*n*-hexane (10 mL, 1:5, *v*/*v*). For the synthesis of compounds **4c**, **4d**, and **4f**, the residue was recrystallized from EtOH (10 mL). Yield: 217 mg (0.58 mmol, 58%); yellow solid of m.p., 66–67 °C; *υ*_max_(ATR)/cm^−1^ 3224, 3055, 2959, 2927, 2869, 1651, 1621, 1586, 1549, 1515, 1488, 1449, 1361, 1300, 1224, 1171, 1148, 1100, 1050, 953, 919, 870, 793, 741, and 706; ^1^H NMR (300 MHz, CDCl_3_) *δ* 1.23 (6 H, d, J = 6.9 Hz), 1.32 (3 H, d, J = 6.9 Hz), 2.7–2.8 (2 H, m), 2.8–2.9 (1 H, m), 3.0–3.1 (1 H, m), 7.0–7.1 (3 H, m), 7.2–7.3 (3 H, m), 7.4–7.5 (2 H, m), 7.6–7.7 (2 H, m), 7.9–8.0 (1 H, br s), 9.3–9.4 (1 H, br s), and 11.0–11.1 (1 H, br s); ^13^C NMR (75.5 MHz, CDCl_3_) *δ* 22.3, 24.0, 34.1, 38.3, 40.3, 112.7, 115.8, 124.2, 124.6, 125.3, 126.3, 126.7, 128.6, 128.9, 137.2, 145.3, 149.4, 152.7, and 156.5; HR-MS (ESI, *m/z*) for C_24_H_27_O_2_N_2_ [M + H^+^] calcd. 375.20670, found 375.20593.

#### 4.1.16. *N*′-3-(3-Isopropylphenyl)butanylidene-2-hydroxybenzoylhydrazide (**4b**)

Analogously to the synthesis of **4a**, compound **4b** was obtained from 2-hydroxybenzoic hydrazide (152 mg, 1.0 mmol) and florhydral^TM^ (190 mg, 1.0 mmol). Yield: 198 mg (0.61 mmol, 61%); colorless oil; *υ*_max_(ATR)/cm^−1^ 3232, 3073, 2960, 2927, 2870, 1640, 1593, 1558, 1489, 1456, 1356, 1307, 1255, 1225, 1172, 1148, 1106, 1049, 1036, 947, 904, 860, 818, 794, 753, 732, 706, and 667; ^1^H NMR (300 MHz, CDCl_3_) *δ* 1.22 (6 H, d, J = 6.9 Hz), 1.31 (3 H, d, J = 7.0 Hz), 2.6–2.7 (2 H, m), 2.7–2.8 (1 H, m), 2.9–3.0 (1 H, m), 6.8–6.9 (1 H, m), 7.0–7.2 (4 H, m), 7.3–7.5 (2 H, m), 8.9–9.0 (1 H, br s), and 11.8–11.9 (1 H, br s); ^13^C NMR (75.5 MHz, CDCl_3_) *δ* 22.3, 24.0, 34.1, 38.3, 40.3, 112.9, 118.8, 118.9, 124.2, 124.6, 125.2, 128.6, 134.8, 145.3, 149.4, 152.1, and 162.1; HR-MS (ESI, *m/z*) for C_20_H_25_O_2_N_2_ [M + H^+^] calcd. 325.19105, found 325.19044.

#### 4.1.17. *N*′-3-(3-Isopropylphenyl)butanylidene-3-hydroxybenzoylhydrazide (**4c**)

Analogously to the synthesis of **4a**, compound **4c** was obtained from 3-hydroxybenzoic hydrazide (85 mg, 152 mg, 0.56 mmol) and florhydral^TM^ (106 mg, 0.56 mmol). Yield: 192 mg (0.59 mmol, 59%); colorless solid of m.p., 196–197 °C; *υ*_max_(ATR)/cm^−1^ 3233, 3058, 2960, 2892, 1645, 1627, 1595, 1539, 1445, 1350, 1297, 1244, 1230, 1183, 1171, 1127, 1081, 1059, 1047, 1020, 998, 986, 954, 941, 922, 880, 821, 796, 751, 706, 674, and 685; ^1^H NMR (300 MHz, CDCl_3_/DMSO-*d*_6_) *δ* 0.9–1.0 (6 H, m), 1.08 (3 H, d, J = 7.0 Hz), 2.4–2.5 (2 H, m), 2.5–2.8 (4 H, m), 6.7–6.8 (1 H, m), 6.8–6.9 (3 H, m), 7.0–7.2 (4 H, m), 7.3–7.4 (1 H, m), 8.89 (1 H, s), and 10.66 (1 H, s); ^13^C NMR (75.5 MHz, CDCl_3_/DMSO-*d*_6_) *δ* 21.6, 23.6, 33.5, 37.7, 114.5, 117.3, 118.0, 118.3, 123.7, 124.7, 127.9, 128.7, 128.9, 134.5, 145.5, 148.5, 150.9, 156.9, and 163.9; HR-MS (ESI, *m/z*) for C_20_H_25_O_2_N_2_ [M + H^+^] calcd. 325.19105, found 325.19072.

#### 4.1.18. *N*′-3-(3-Isopropylphenyl)butanylidene-2-fluorobenzoylhydrazide (**4d**)

Analogously to the synthesis of **4a**, compound **4d** was obtained from 2-fluorobenzoic hydrazide (154 mg, 1.0 mmol) and florhydral^TM^ (190 mg, 1.0 mmol). Yield: 187 mg (0. 57 mmol, 57%); colorless oil; ^1^H NMR (300 MHz, CDCl_3_) *δ* 1.1–1.2 (6 H, m), 1.29 (3 H, d, J = 7.0 Hz), 2.6–2.7 (2 H, m), 2.8–2.9 (1 H, m), 2.9–3.1 (1 H, m), 7.0–7.1 (5 H, m), 7.1–7.2 (3 H, m), 7.4–7.5 (3 H, m), 8.0–8.1 (2 H, m), and 9.4–9.5 (1 H, m); ^13^C NMR (75.5 MHz, CDCl_3_) *δ* 21.9, 23.9, 34.0, 38.1, 40.4, 115.6–116.0 (m), 119.8, 123.7, 124.0, 124.3, 124.7, 125.0. 125.1, 128.7, 131.6, 132.1, 132.4, 145.4, 149.1, 151.7, 158.4–161.6 (m), 159.8, and 164.3; HR-MS (ESI, *m/z*) for C_20_H_24_ON_2_F [M + H^+^] calcd. 327.18672, found 327.18693.

#### 4.1.19. *N*′-3-(3-Isopropylphenyl)butanylidene-3-chlorobenzoylhydrazide (**4f**)

Analogously to the synthesis of **4a**, compound **4f** was obtained from 3-chlorobenzoic hydrazide (171 mg, 1.0 mmol) and florhydral^TM^ (190 mg, 1.0 mmol). Yield: 206 mg (0.60 mmol, 60%); colorless oil; *υ*_max_(ATR)/cm^−1^ 3220, 3067, 2960, 2870, 1648, 1602, 1558, 1488, 1459, 1427, 1362, 1289, 1265, 1132, 1079, 1050, 948, 914, 794, 738, 706, 680, and 657; ^1^H NMR (300 MHz, CDCl_3_) *δ* 1.22 (6 H, d, J = 6.9 Hz), 1.30 (3 H, d, J = 6.9 Hz), 2.7–2.8 (2 H, m), 2.8–2.9 (1 H, m), 2.9–3.0 (1 H, m), 7.0–7.1 (3 H, m), 7.2–7.3 (1 H, m), 7.3–7.4 (2 H, m), 7.4–7.5 (1 H, m), 7.63 (1 H, d, J = 7.7 Hz), 7.75 (1 H, s), and 8.8–8.9 (1 H, br s); ^13^C NMR (75.5 MHz, CDCl_3_) *δ* 22.2, 24.0, 34.1, 38.3, 40.3, 124.2, 124.5, 125.3, 127.5, 128.6, 130.0, 132.0, 134.9, 145.4, 149.3, 151.8, and 162.6; HR-MS (ESI, *m/z*) for C_20_H_24_ON_2_Cl [M + H^+^] calcd. 343.15717, found 343.15640.

#### 4.1.20. *N*′-3-(3-Isopropylphenyl)butanylidene-isonicotinoylhydrazide (**4g**)

Analogously to the synthesis of **4a**, compound **4g** was obtained from isoniazid (138 mg, 1.0 mmol) and florhydral^TM^ (190 mg, 1.0 mmol). Yield: 194 mg (0.57 mmol, 57%); colorless gum; *υ*_max_(ATR)/cm^−1^ 3215, 3048, 2960, 2871, 1653, 1629, 1602, 1549, 1488, 1459, 1409, 1362, 1294, 1217, 1154, 1131, 1086, 1067, 1049, 993, 949, 891, 843, 794, 754, 735, 706, and 677; ^1^H NMR (300 MHz, CDCl_3_) *δ* 1.21 (6 H, d, J = 6.9 Hz), 1.30 (3 H, d, J = 6.8 Hz), 2.5–2.6 (1 H, m), 2.6–2.7 (2 H, m), 2.8–2.9 (1 H, m), 3.0–3.1 (1 H, m), 7.0–7.1 (4 H, m), 7.1–7.2 (1 H, m), 7.4–7.5 (1 H, m), 7.6–7.6 (2 H, m), 8.6–8.8 (2 H, m), and 9.10 (1 H, s); ^13^C NMR (75.5 MHz, CDCl_3_) *δ* 22.2, 24.0, 34.1, 37.9, 40.4, 121.0, 123.5, 124.2, 124.5, 125.2, 128.6, 140.3, 145.2, 147.9, 149.3, 149.7, 150.7, 152.9, and 161.9; HR-MS (ESI, *m/z*) for C_19_H_24_ON_3_ [M + H^+^] calcd. 310.19139, found 310.19048.

### 4.2. Toxoplasma gondii Cells Drug Testing Assay 

Cells of the Vero cell line (ATCC^®^ CCL81™, Manassas, VA, USA) were used to cultivate *T. gondii* RH strain tachyzoites. Vero cells were maintained in complete RPMI 1640 medium (Thermo Fisher Scientific, Waltham, MA, USA). For cell cultivation, 96-well plates were prepared with 5 × 10^3^ cells per well in 200 μL of RPMI 1640 medium, then incubated at 37 °C and 5% CO_2_ for 24 h. The medium was then removed, and cells were washed with phosphate-buffered saline (PBS). Next, RPMI 1640 medium with 2% FBS and parasites at a 5:1 parasite-to-cell ratio were added. After a 5 h incubation at 37 °C and 5% CO_2_, cells were washed with PBS and subjected to further treatments. RPMI 1640 medium with 1% DMSO was used as the negative control, while the experimental compounds and the positive control drug (ATO) were dissolved in 1% DMSO with RPMI 1640 medium leading to final concentrations of 50, 25, 12.5, 6.25, 3.13, 1.65, and 0.75 μg/mL. After incubation at 37 °C and 5% CO_2_ for 72 h, cells were washed with PBS, fixed in 10% formalin, and stained with 1% toluidine blue. An inverted photomicroscope was used to assess cell infection and to determine the infection index (number of infected cells out of 200 counted cells). The inhibition percentage was calculated as follows:

Inhibition (%) = [(I Control − I Experimental)/I Control] × 100

where “I Control” represents the infection index of untreated cells, and “I Experimental” represents the infection index of cells treated with test compounds. The effects of the compounds on parasite growth are expressed as IC_50_ values derived from three independent experiments [44,58].

### 4.3. Leishmania major Drug Testing Assays

Promastigotes of *L. major* were obtained from a Saudi patient (February 2016) and cultured at 26 °C in Schneider’s Drosophila medium (Invitrogen, Carlsbad, CA, USA) supplemented with 10% heat-inactivated fetal bovine serum (FBS, Invitrogen, USA) and antibiotics, with weekly transfers in a tissue culture flask. Promastigotes were cryopreserved in liquid nitrogen at a concentration of 3 × 10^6^ parasites/mL. Virulent *L. major* parasites were maintained by serial passage in female BALB/c mice, achieved by injecting hind footpads with 1 × 10^6^ stationary-phase promastigotes. Amastigotes were isolated from the mice after 8 weeks, then converted to promastigotes by cultivation at 26 °C in Schneider’s medium containing 10% FBS and antibiotics. Amastigote-derived promastigotes, which underwent several in vitro passages, were used for the infection studies. BALB/c mice (both male and female) were provided by the Pharmaceutical College, King Saud University, Kingdom of Saudi Arabia, and housed in specific pathogen-free conditions following the ethical guidelines of the Qassim University Research Ethics Committee (permission number: 20-03-20).

*L. major* promastigotes in the logarithmic phase were cultured in phenol-red-free RPMI 1640 medium (Invitrogen, USA) with 10% FBS in 96-well plates at 10^6^ cells/mL (200 μL/well, counted with a hemocytometer). Test compounds were added at final concentrations of 50, 25, 12.5, 6.25, 3.13, 1.65, and 0.75 μg/mL. Negative controls contained 1% DMSO without test compounds, and positive controls included amphotericin B at decreasing concentrations (50 to 0.75 μg/mL). After incubation at 26 °C for 72 h, viable promastigotes were counted using an MTT colorimetric assay. The formed formazan was dissolved in a detergent and the absorbance was measured at 570 nm using an ELISA reader. IC_50_ values were derived from three independent experiments.

Activity against amastigotes in macrophages was evaluated using peritoneal macrophages, which were harvested from 6–8-week-old BALB/c mice of different sex, and plated at 5 × 10^4^ cells/well in 96-well plates containing phenol-red-free RPMI 1640 medium with 10% FBS. After 4 h at 37 °C and 5% CO_2_ to promote adhesion, cells were washed with PBS, and 200 μL of an *L. major* promastigote solution (10 promastigotes per macrophage) was added. Plates were incubated for 24 h at 37 °C in a 5% CO_2_ environment to allow infection and amastigote differentiation. Infected cells were then washed with PBS and overlaid with RPMI 1640 medium containing the test compounds at the same concentrations as applied for the experiments with promastigotes, followed by incubation at 37 °C and 5% CO_2_ for 72 h. Negative controls only had 1% DMSO, while positive controls contained amphotericin B. The percentage of infected macrophages was determined microscopically after fixation, Giemsa staining, and medium removal. IC_50_ values were calculated from three independent experiments [44,59]. 

### 4.4. Vero Cell and Macrophage Cytotoxicity 

To assess the test compounds’ cytotoxicity, MTT assays were performed. The Vero cells and macrophages were cultivated for 24 h at 37 °C in RPMI 1640 media with 10% FBS and 5% CO_2_ in 96-well plates (5 × 10^3^ cells/well/200 μL). Following a washing step with PBS, the cells were treated with test compounds at different concentrations (50, 25, 12.5, 6.25, 3.13, 1.65, and 0.75 μg /mL) in 10% FBS medium for 72 h. Negative control cells were only given media with 2% FBS and 1% DMSO. The cells were cultured for 4 h after the supernatant was disposed of, and 50 μL of RPMI 1640 media containing 14 μL MTT (5 mg/mL) was added. After removing the supernatant once again, 150 μL of DMSO was added. IC_50_ values were determined from three independent experiments [44,60].

## 5. Conclusions

The preparation of acyl hydrazones from cinnamaldehyde led to the identification of antitoxoplasmal compounds (e.g., **2a** and **2c**) with considerable selectivities which warrants more in-depth investigation of these compounds. The underlying modes of action remain to be elucidated but are probably similar to reported mechanisms of cinnamaldehyde and hydrazone analogs such as dynasore based on the importance of the 3-hydroxy-2-naphthoyl and salicyloyl moieties for activity. Antileishmanial activities of the hydrazones were less pronounced, however, the moderately active acyl hydrazone **4a** from florhydral^TM^ was remarkable, since it indicates that synthetic fragrances can become a valuable source of antiparasitic agents, too. Future studies will show whether the described compounds have the potential to advance to clinical stages of drug testing, in particular for toxoplasmosis treatment. 

## Figures and Tables

**Table 1:** Activities (expressed as IC_50_ values ± SD) of the test compounds against *T. gondii* parasites and Vero monkey kidney cells. Atovaquone (ATO) was used as positive control.

Compound	*T. gondii* (µM)	Vero (µM)	SI ^1^
Cinnamaldehyde	>151.3	>151.3	-
CPH	12.9 ± 2.1	23.6 ± 3.9	1.8
Silvial^TM^	90.1 ± 15.2	>97.9	-
Florhydral^TM^	>105.1	99.1 ± 18.4	-
**2a**	0.63 ± 0.08	7.3 ± 0.9	11.5
**2b**	23.4 ± 3.9	24.2 ± 2.7	1.0
**2c**	1.1 ± 0.2	19.5 ± 2.6	17.3
**2d**	3.9 ± 0.5	22.0 ± 3.0	5.7
**2e**	>49.6	46.2 ± 6.4	-
**2f**	>42.7	40.1 ± 6.8	-
**2g**	69.4 ± 10.0	>74.6	-
**2h**	55.2 ± 9.7	54.1 ± 9.1	0.98
**2i**	39.1 ± 6.4	44.0 ± 6.7	1.1
**2j**	>42.4	>42.4	-
**2k**	>40.3	>40.3	-
**2l**	52.1 ± 8.3	51.3 ± 7.8	0.99
**2m**	31.6 ± 6.2	41.5 ± 7.2	1.3
**3a**	46.9 ± 8.4	41.3 ± 6.9	0.88
**3b**	13.7 ± 2.2	24.6 ± 3.3	1.8
**3c**	55.3 ± 9.4	>59.1	-
**3d**	46.3 ± 5.5	56.1 ± 10.4	1.2
**3e**	45.3 ± 6.9	53.5 ± 8.5	1.2
**3f**	22.0 ± 3.3	50.2 ± 7.7	2.3
**3g**	60.6 ± 10.1	>61.8	-
**4a**	14.4 ± 2.1	39.6 ± 4.8	2.8
**4b**	21.0 ± 3.5	19.2 ± 2.2	0.92
**4c**	>61.7	29.1 ± 2.6	-
**4d**	>61.3	>61.3	-
**4f**	47.5 ± 7.3	57.9 ± 11.0	1.2
**4g**	>64.6	>64.6	-
ATO ^2^	0.07 ± 0.01	9.5 ± 0.8	135

^1^ SI = selectivity index (Vero/*T. gondii*). ^2^ Values also present in Reference [44].

**Table 2:** Activities (expressed as IC_50_ values ± SD) of test compounds against *L. major* parasites (promastigotes and amastigotes) and macrophages. Amphotericin B (AmB) was used as the positive control.

Compound	Promastigotes (µM)	Amastigotes (µM)	Macrophages (µM)	SI (Promastigotes) ^1^
Cinnamaldehyde	>151.3	>151.3	>151.3	-
CPH	83.7 ± 13.6	-	78.4 ± 12.2	0.94
Silvial^TM^	>97.9	>97.9	>97.9	-
Florhydral^TM^	>105.1	>105.1	>105.1	-
**2a**	16.8 ± 2.8	>41.7	>41.7	>2.5
**2b**	>36.7	>36.7	>36.7	-
**2c**	47.7 ± 6.5	>49.6	43.6 ± 8.6	0.91
**2d**	>42.7	>42.7	>42.7	-
**2e**	>49.6	>49.6	>49.6	-
**2f**	>42.7	>42.7	>42.7	-
**2g**	>74.6	>74.6	>74.6	-
**2h**	>64.2	>64.2	>64.2	-
**2i**	>49.2	>49.2	>49.2	-
**2j**	>42.4	>42.4	>42.4	-
**2k**	>40.3	>40.3	>40.3	-
**2l**	51.3 ± 9.3	>52.5	>52.5	>1.0
**2m**	>44.8	>44.8	>44.8	-
**3a**	>51.5	>51.5	>51.5	-
**3b**	>59.1	58.6 ± 8.6	>59.1	-
**3c**	>59.1	>59.1	>59.1	-
**3d**	>58.8	>58.8	>58.8	-
**3e**	>58.8	>58.8	>58.8	-
**3f**	29.1 ± 3.3	>56.0	>56.0	>1.9
**3g**	>61.8	>61.8	>61.8	-
**4a**	9.6 ± 2.4	>53.4	>53.4	>5.6
**4b**	39.5 ± 5.9	45.4 ± 7.9	50.3 ± 9.4	1.3
**4c**	>61.7	>61.7	>61.7	-
**4d**	>61.3	>61.3	>61.3	-
**4f**	27.9 ± 4.2	>58.3	>58.3	>2.1
**4g**	54.6 ± 9.0	>64.6	>64.6	>1.2
AmB ^2^	0.83 ± 0.2	0.47 ± 0.1	8.1 ± 1.8	9.8

^1^ SI = selectivity index (macrophages/*L. major* promastigotes). ^2^ Values also present in Reference [44].

## Data Availability

Original data can be obtained from the authors upon reasonable request.

## Supplementary Materials

The following supporting information can be downloaded at: https://www.mdpi.com/article/10.3390/antibiotics13121114/s1, ^1^H NMR data of known test compounds (**2a**–**c**, **2g**, **2l**, and **2m**), original NMR spectra of test compounds (Figures S1–S46), and original HRMS spectra of new compounds (Figures S47–S66).
