# Supplementary material for: Antiparasitic Activities of Acyl Hydrazones from Cinnamaldehydes and Structurally Related Fragrances
Source: Antibiotics (Basel). 2024 Nov 22;13(12):1114. doi: 10.3390/antibiotics13121114 (PMC11672724; doi:10.3390/antibiotics13121114)
Supplement: Supplementary file 1 [file antibiotics-13-01114-s001.zip › antibiotics-3317820-supplementary.pdf]

Supplementary Materials

Antiparasitic Activities of Acyl Hydrazones From Cinnamaldehydes and Structurally Related Fragrances

Ibrahim S. Al Nasr [1], Waleed S. Koko [1], Tariq A. Khan [2], Rainer Schobert [3] and Bernhard Biersack [3,*]

[1] Department of Biology, College of Science, Qassim University, Qassim 51452, Saudi Arabia
[2] Department of Clinical Nutrition, College of Applied Health Sciences, Qassim University, Ar Rass 51921, Saudi Arabia
[3] Organic Chemistry Laboratory, University Bayreuth, Universitätsstrasse 30, 95440 Bayreuth, Germany

*Correspondence: bernhard.biersack@yahoo.com

^{1}H NMR data of known cinnamides 2a, 2b, 2c, 2g, 2l, and 2m

(E)-N'-(Cinnamylidene)-3-hydroxy-2-naphtoylhydrazide (2a)
^{1}H NMR (300 MHz, DMSO-d_6) δ 7.1-7.2 (2 H, m), 7.3-7.4 (5 H, m), 7.4-7.5 (1 H, m), 7.5-7.6 (1 H, m), 7.76 (1 H, d, J = 8.2 Hz), 7.91 (1 H, d, J = 8.1 Hz), 8.2-8.3 (1 H, m), 8.43 (1 H, s), 11.31 (1 H, s), 11.89 (1 H, s).

(E)-N'-(4-Dimethylaminocinnamylidene)-3-hydroxy-2-naphthoylhydrazide (2b)
^{1}H NMR (300 MHz, $CDCl_3$) δ 2.96 (6 H, s), 6.64 (2 H, d, J = 8.9 Hz), 6.8-6.9 (2 H, m), 7.2-7.3 (4 H, m), 7.4-7.5 (1 H, m), 7.63 (1 H, d, J = 8.3 Hz), 7.79 (1 H, d, J = 8.1 Hz), 8.17 (1 H, d, J = 8.3 Hz), 8.49 (1 H, s), 11.8-11.9 (1 H, br s).

(E)-N'-(Cinnamylidene)-3-hydroxybenzoylhydrazide (2c)
^{1}H NMR (300 MHz, DMSO-d_6) δ 6.9-7.0 (2 H, m), 7.0-7.1 (1 H, m), 7.3-7.5 (4 H, m), 7.64 (2 H, d, J = 8.3 Hz), 7.8-7.9 (1 H, m), 8.2-8.3 (1 H, m), 11.76 (1 H, s), 11.88 (1 H, s).

(E)-N'-(Cinnamylidene)-2-fluorobenzoylhydrazide (2g)
^{1}H NMR (300 MHz, $CDCl_3$) δ 6.91 (1 H, d, J = 16.1 Hz), 7.1-7.2 (2 H, m), 7.2-7.5 (7 H, m), 7.96 (1 H, d, J = 9.1 Hz), 8.1-8.2 (1 H, m), 9.6-9.7 (1 H, m).

(E)-N'-(Cinnamylidene)-isonicotinoylhydrazide (2l)
^{1}H NMR (300 MHz, DMSO-d_6) δ 7.1-7.2 (2 H, m), 7.3-7.5 (3 H, m), 7.6-7.7 (2 H, m), 7.8-7.9 (2 H, m), 8.2-8.3 (1 H, m), 8.7-8.8 (2 H, m), 11.95 (1 H, s).

(E)-N'-(4-Dimethylaminocinnamylidene)-isonicotinoylhydrazide (2m)
^{1}H NMR (300 MHz, DMSO-d_6) δ 2.96 (6 H, s), 6.6-6.7 (2 H, m), 6.7-6.9 (2 H, m), 7.3-7.4 (2 H, m), 7.7-7.8 (2 H, m), 8.1-8.2 (1 H, m), 8.7-8.8 (2 H, m), 11.75 (1 H, s).

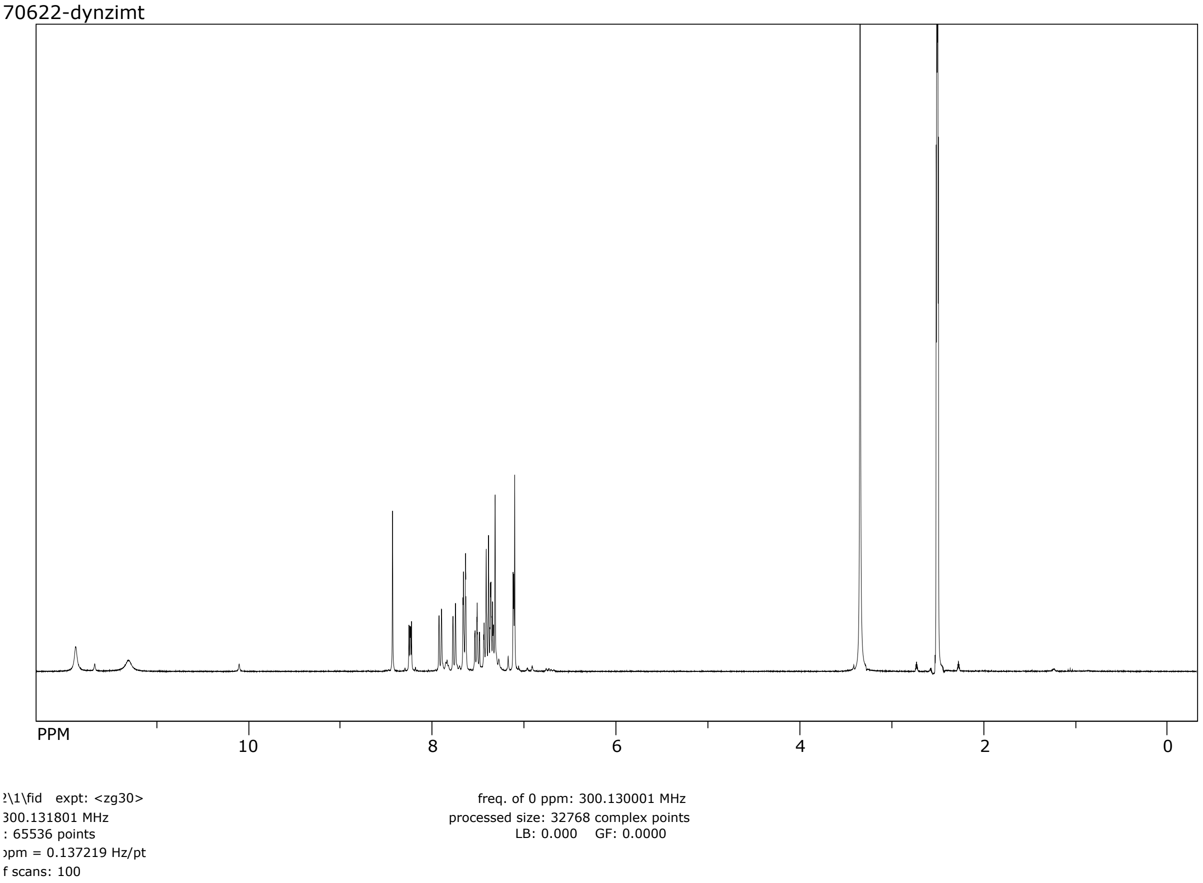

Figure S1. ^{1}H NMR spectrum of 2a.

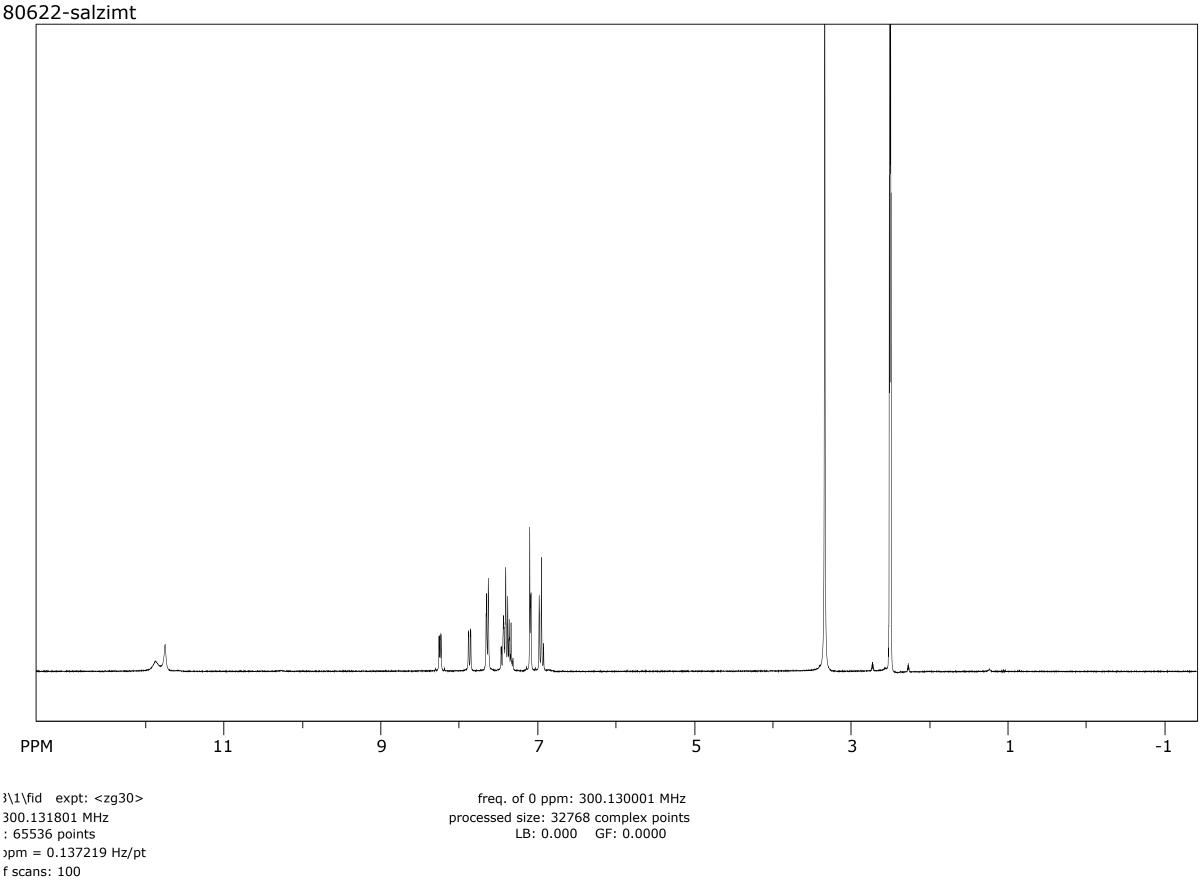

Figure S2. ^{1}H NMR spectrum of 2c.

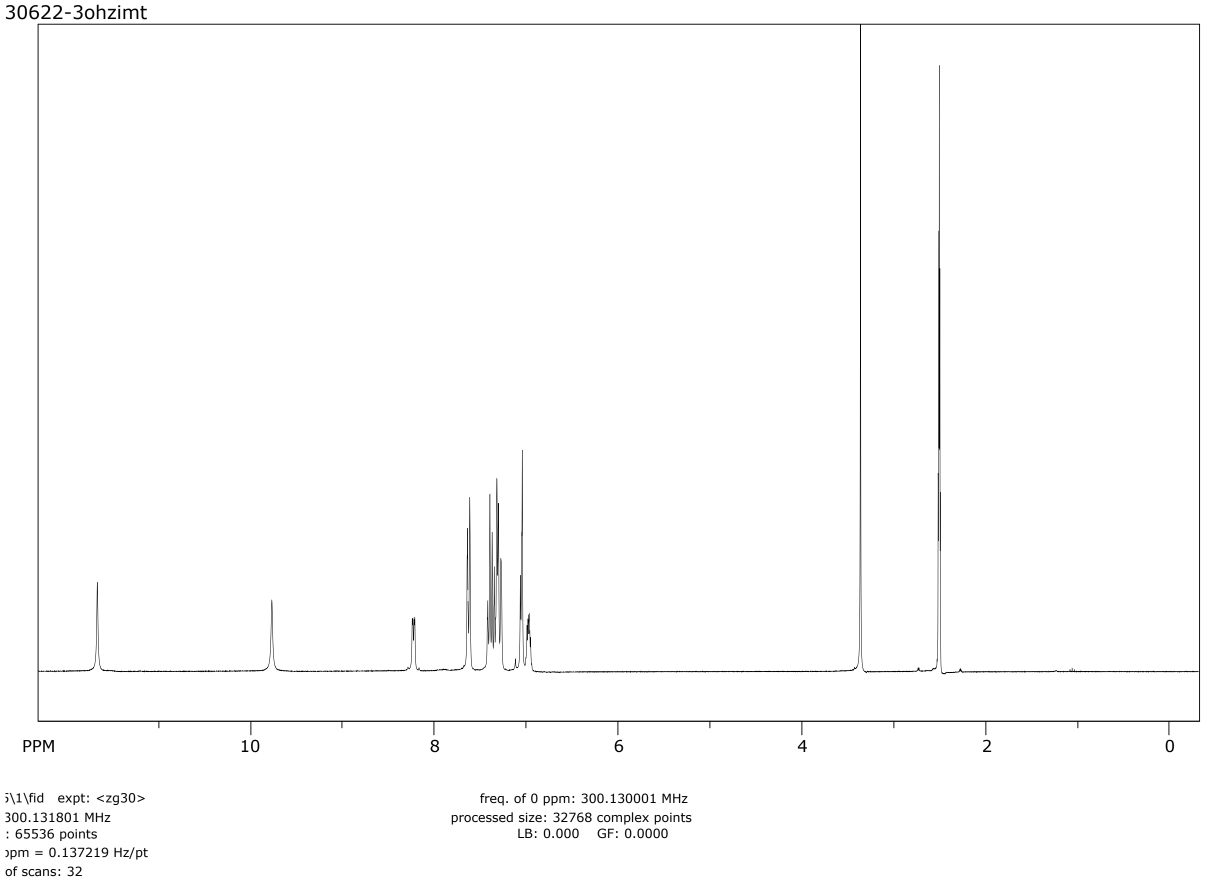

Figure S3. ^{1}H NMR spectrum of 2e.

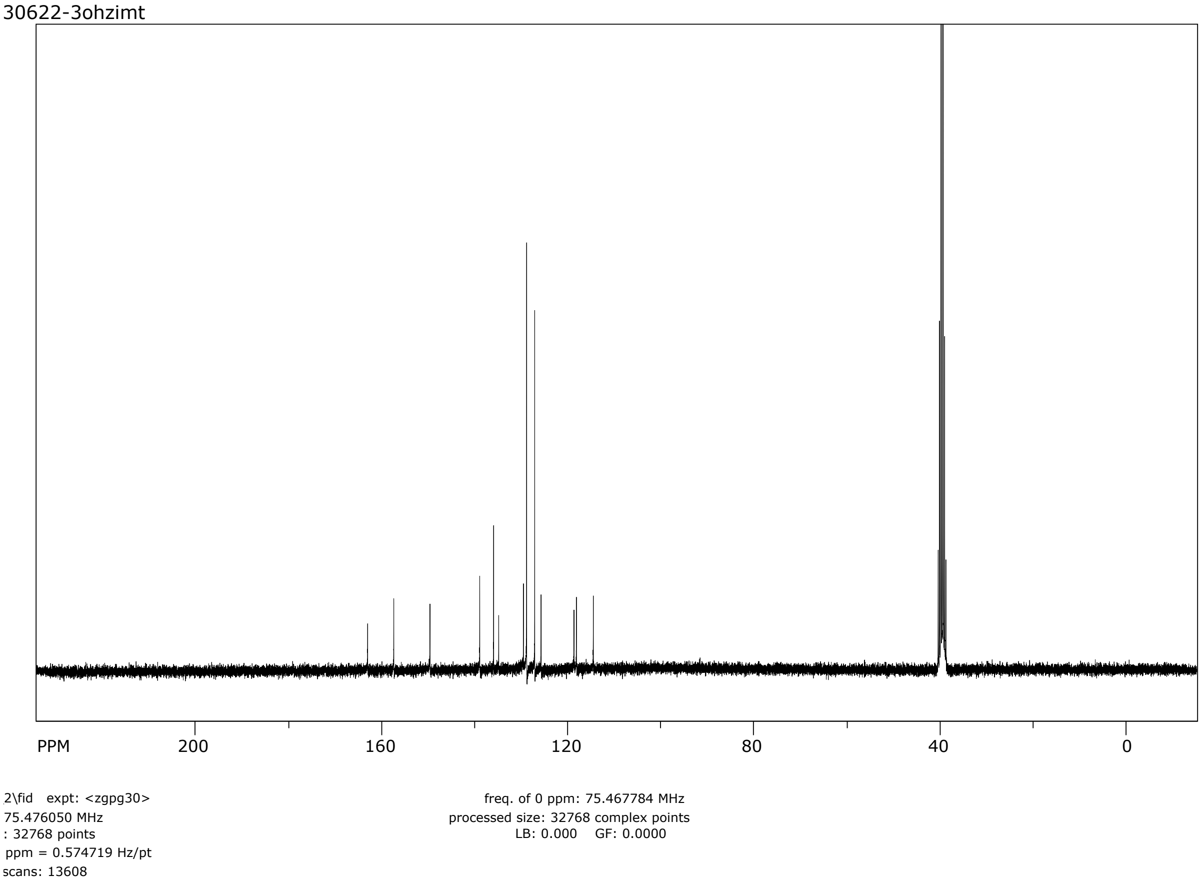

Figure S4. ^{13}C NMR spectrum of 2e.

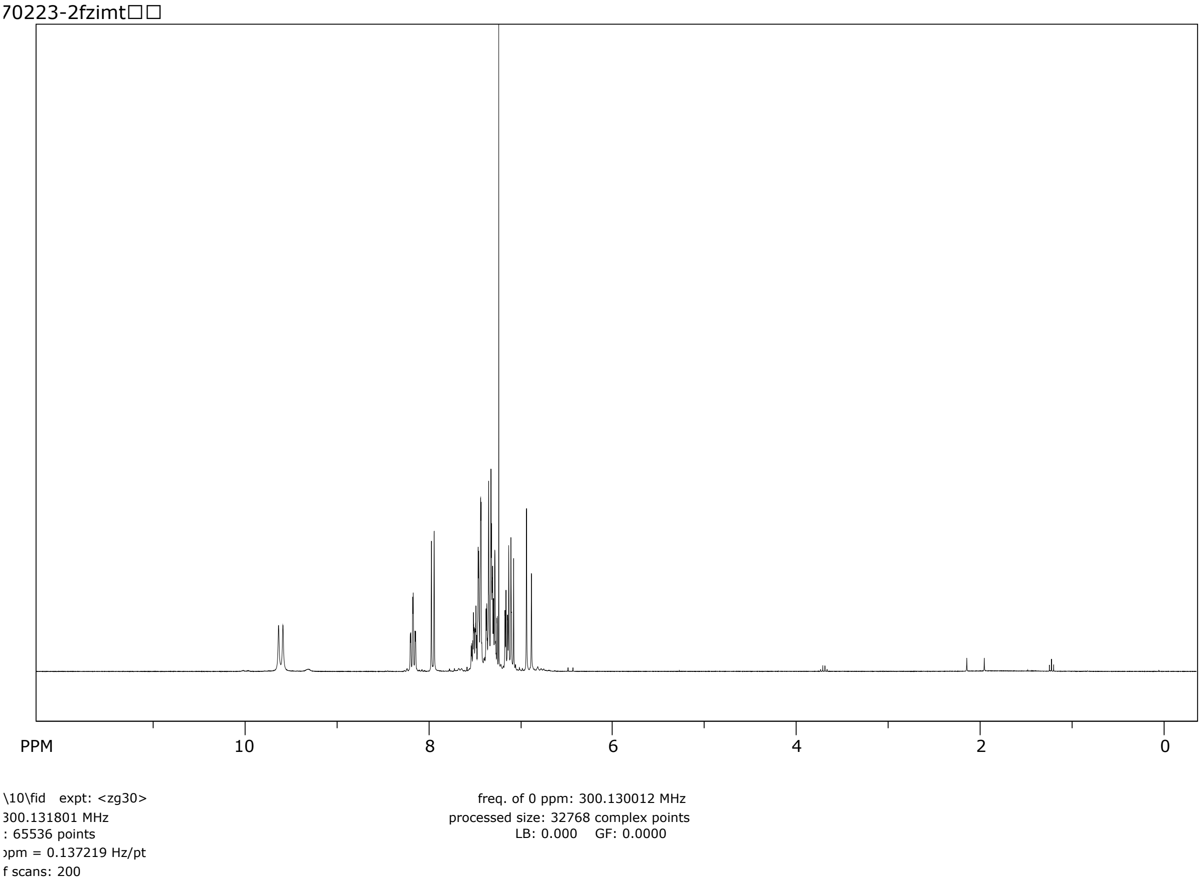

Figure S5. ^{1}H NMR spectrum of 2g.

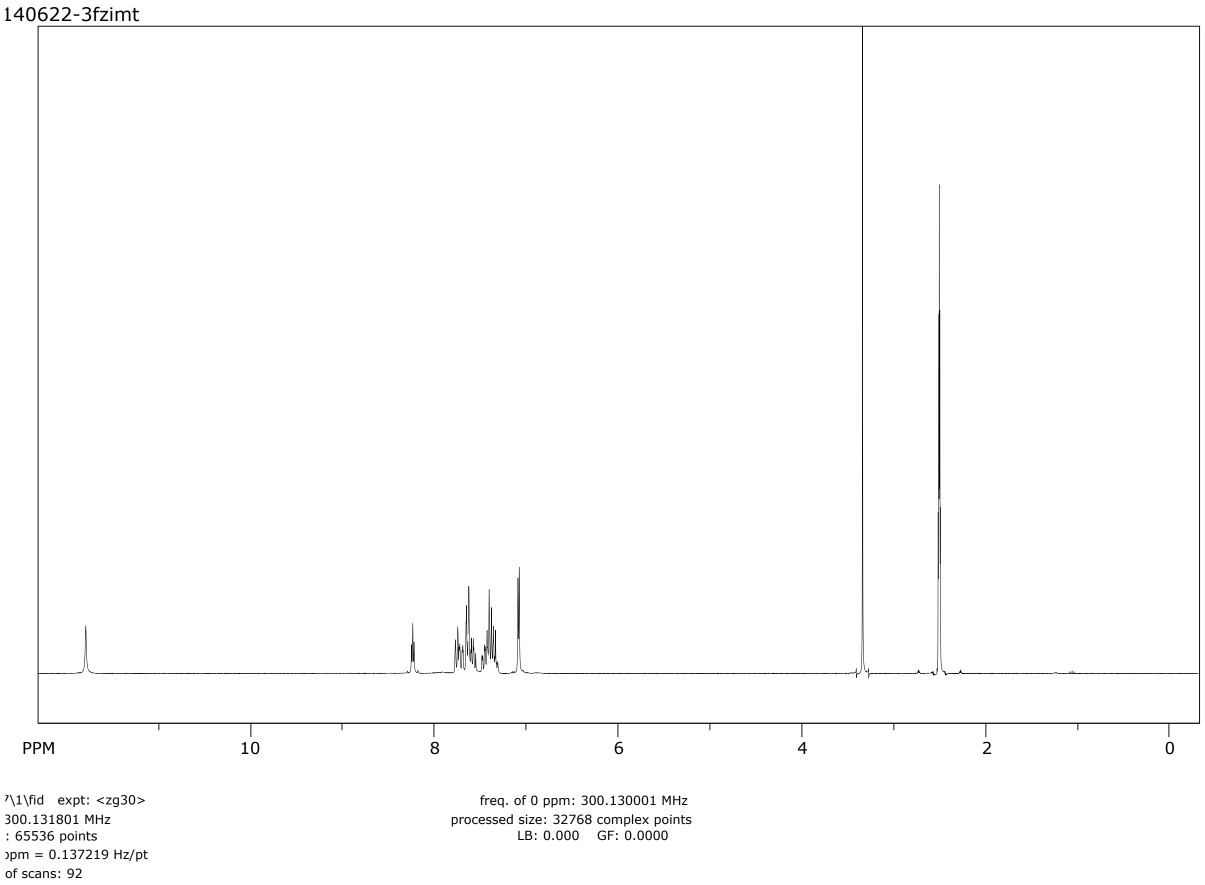

Figure S6. ^{1}H NMR spectrum of 2i.

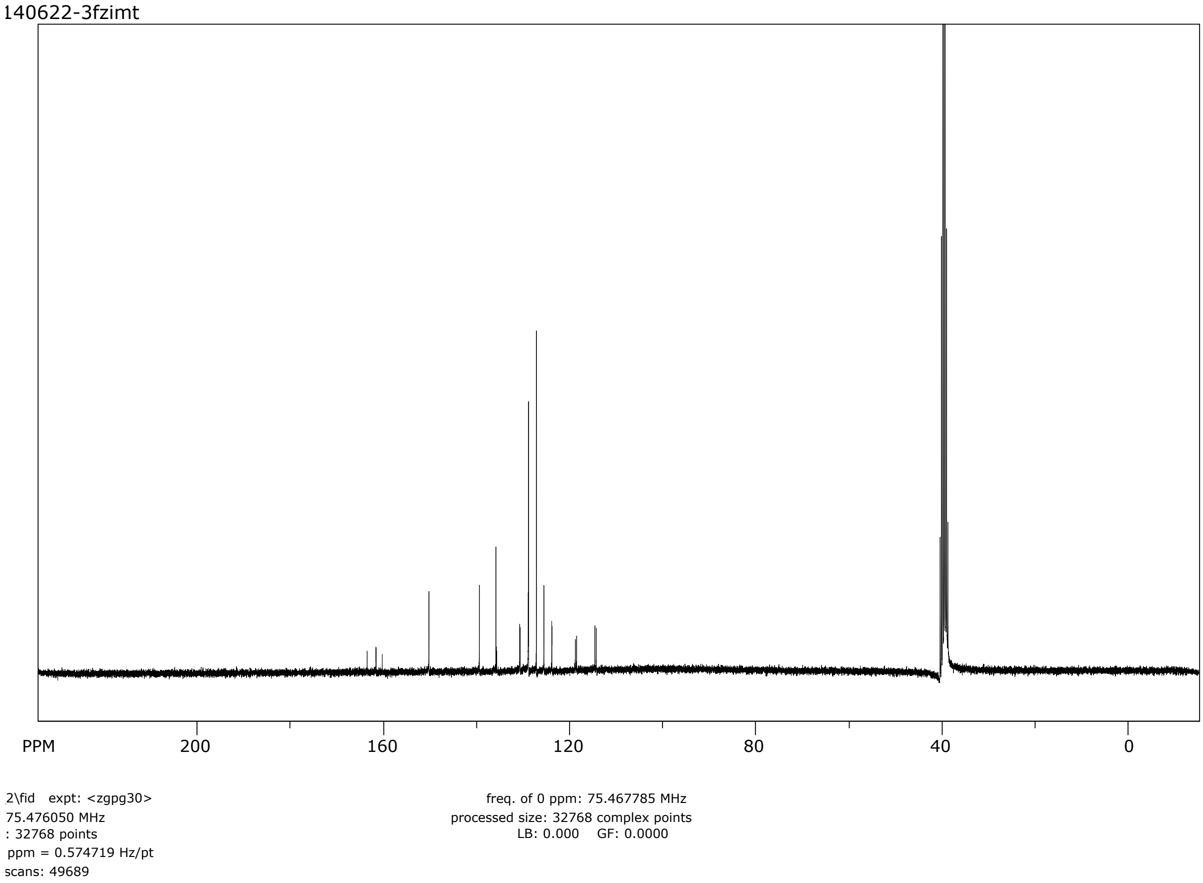

Figure S7. ^{13}C NMR spectrum of 2i.

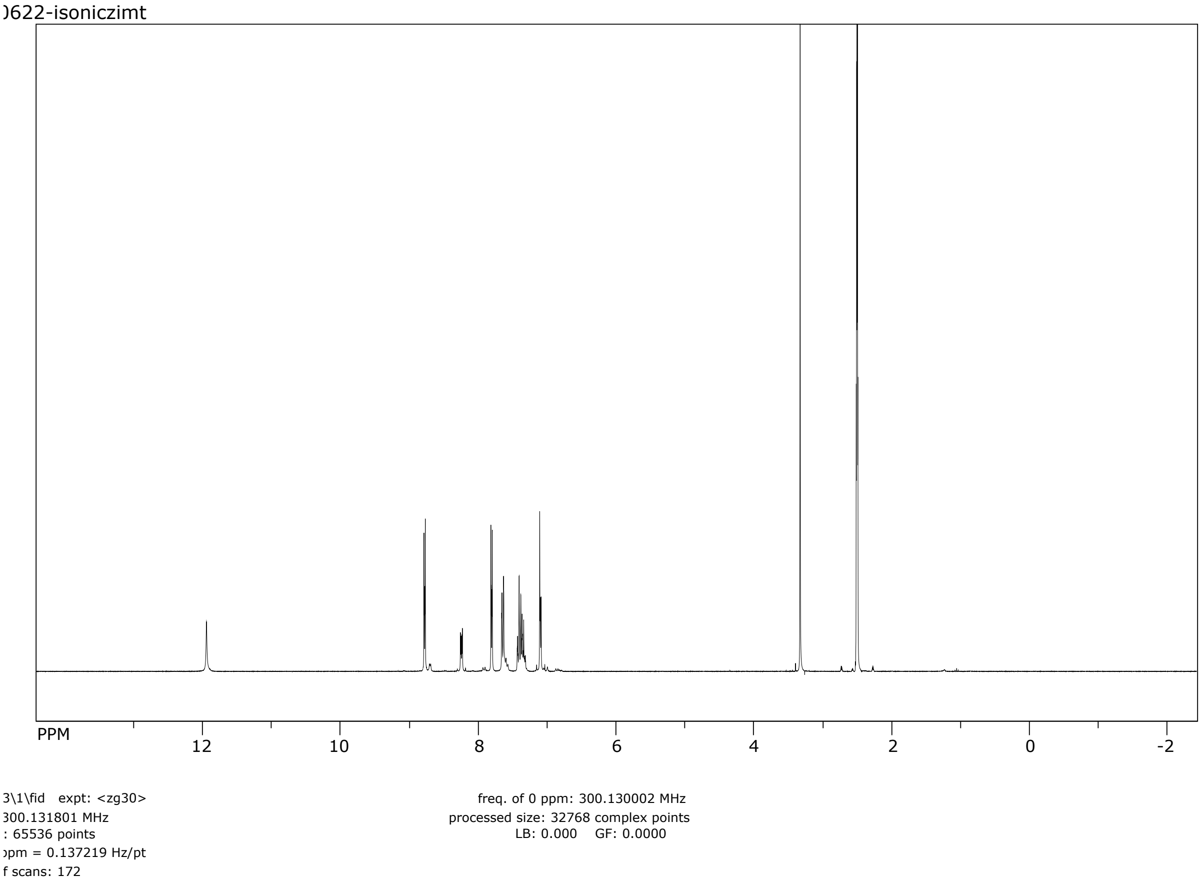

Figure S8. ^{1}H NMR spectrum of 2l.

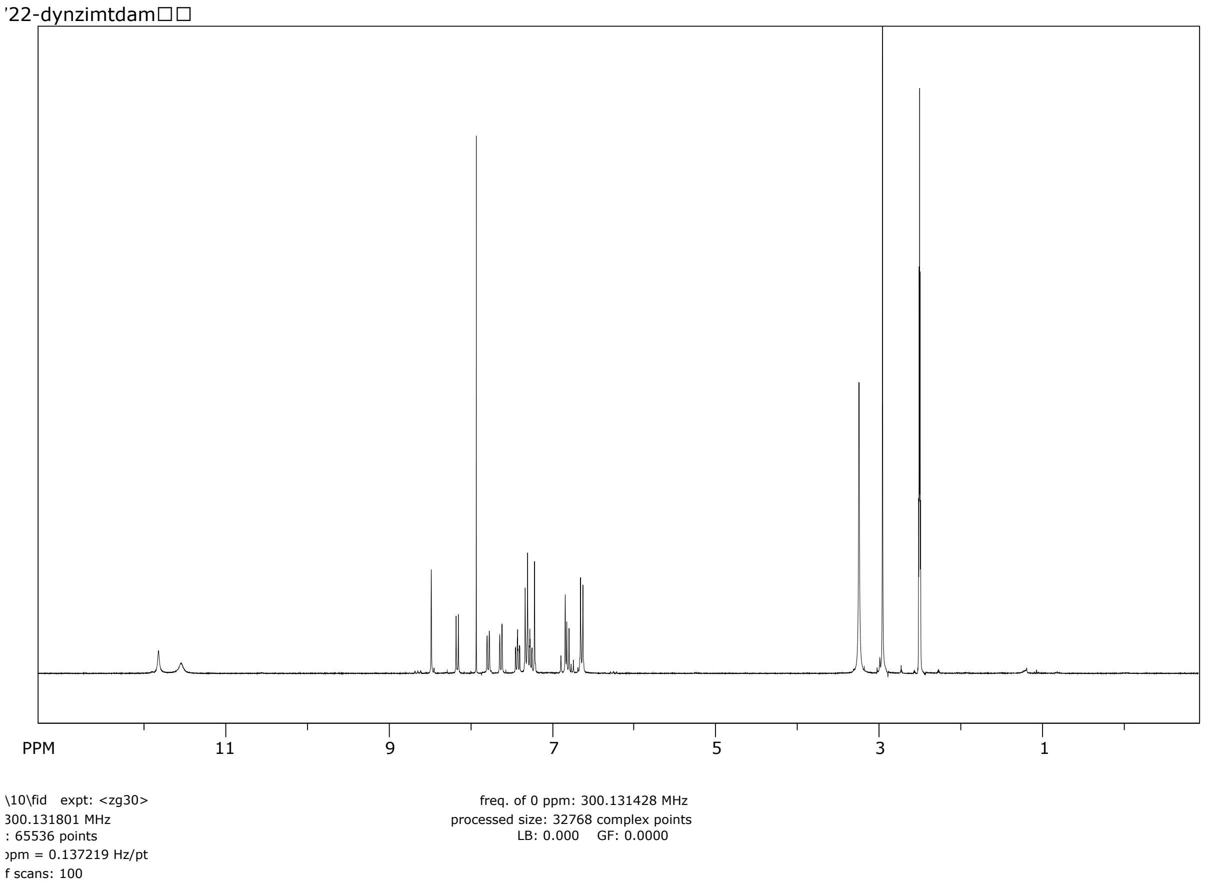

Figure S9. ^{1}H NMR spectrum of 2b.

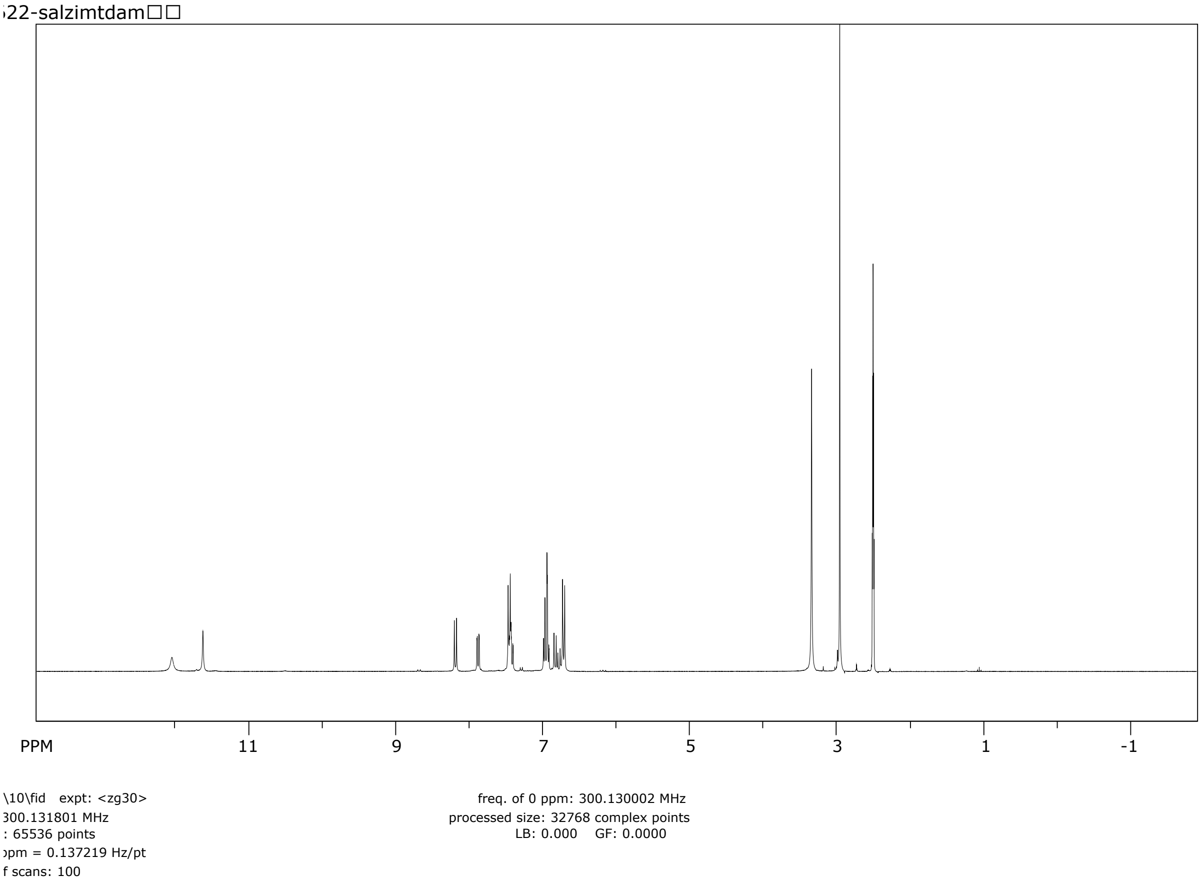

Figure S10. ^{1}H NMR spectrum of 2d.

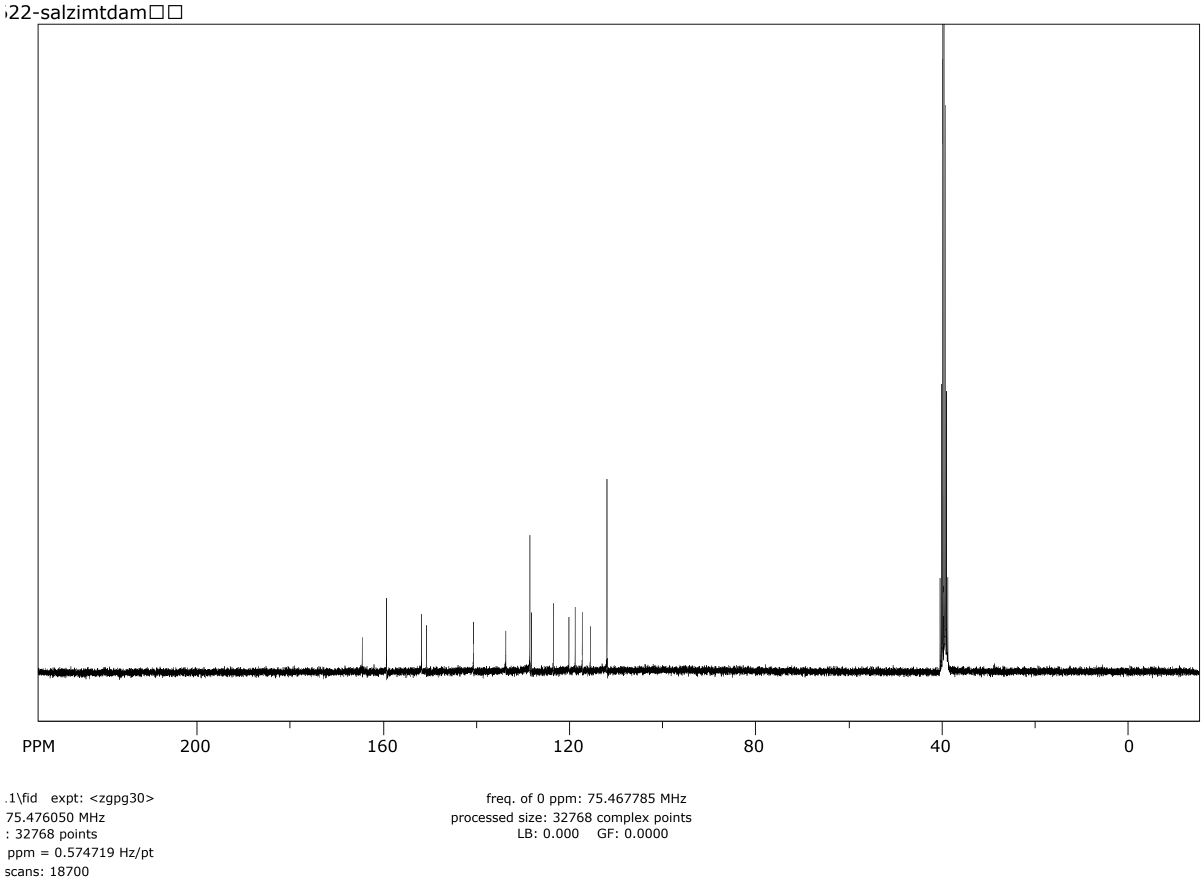

Figure S11. ^{13}C NMR spectrum of 2d.

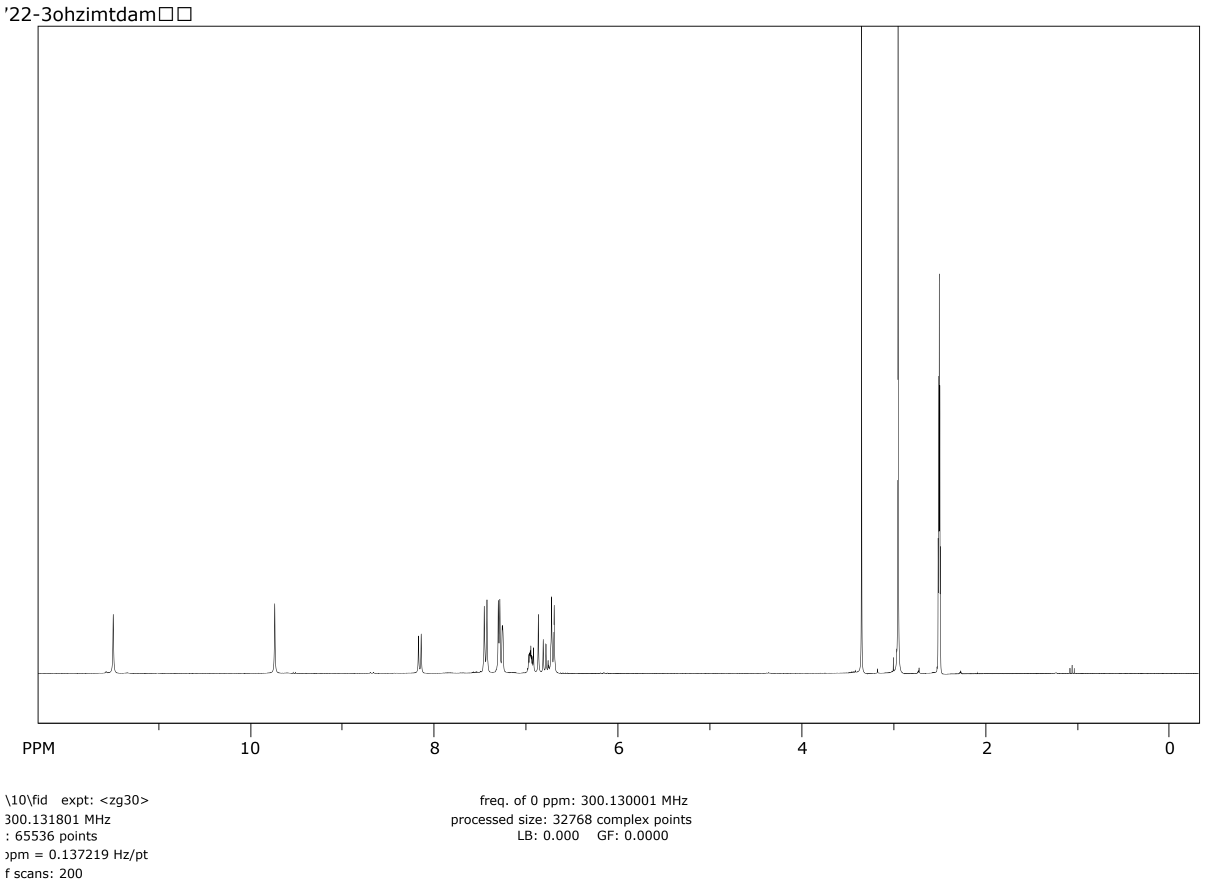

Figure S12. ^{1}H NMR spectrum of 2f.

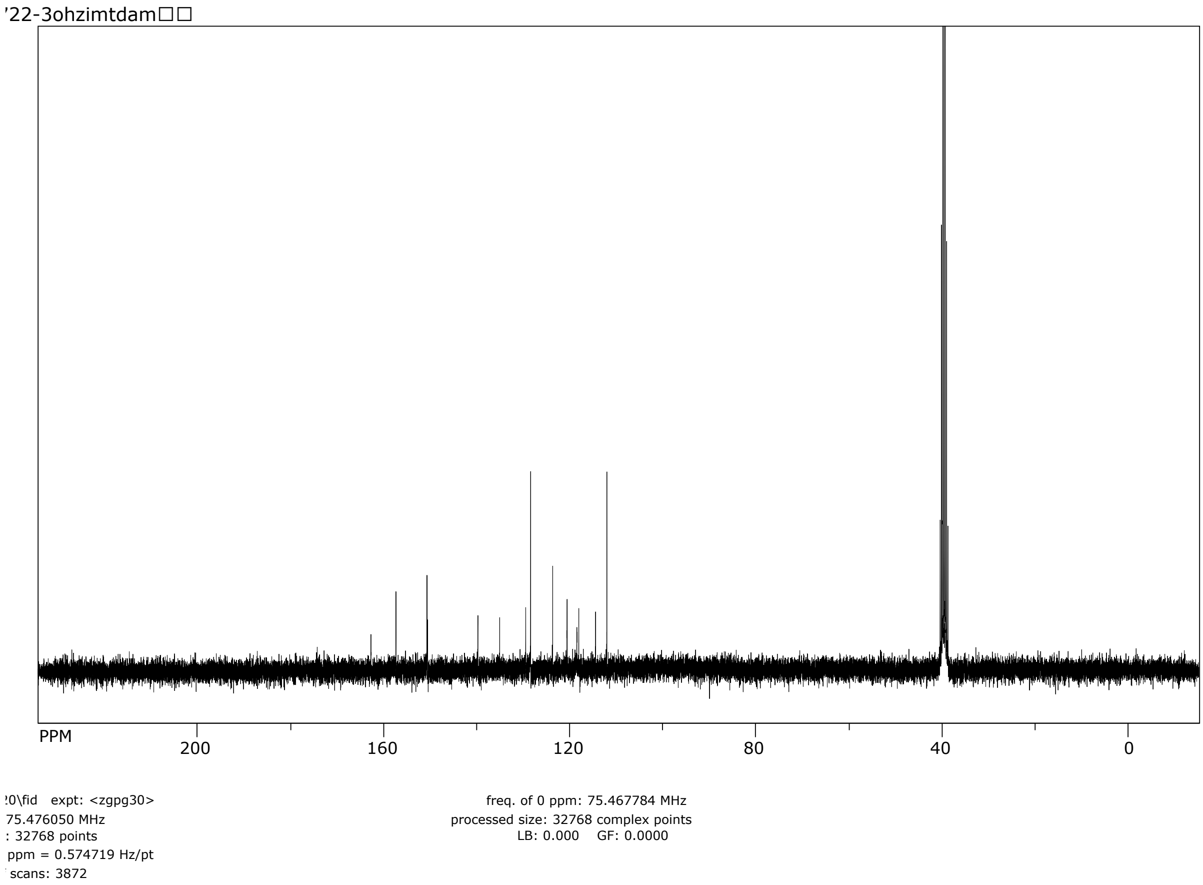

Figure S13. ^{13}C NMR spectrum of 2f.

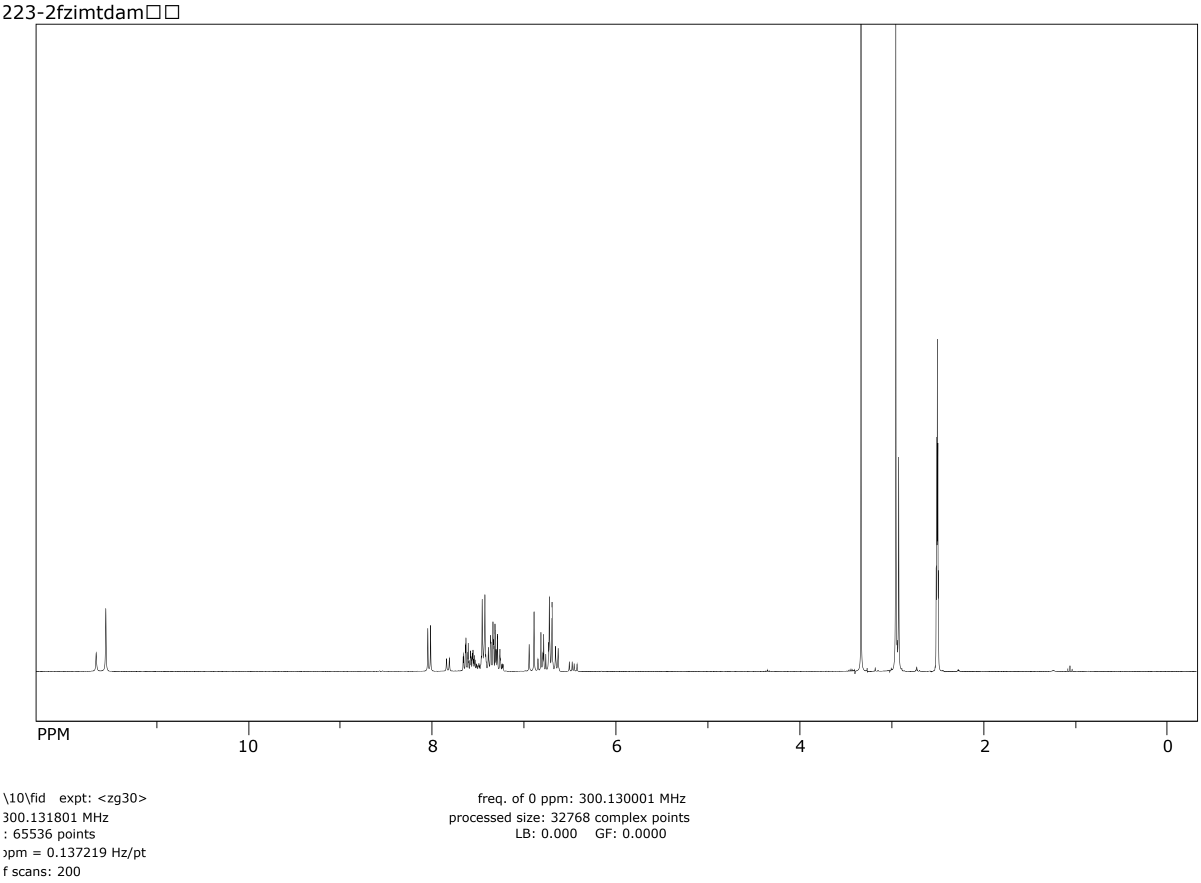

Figure S14. ^{1}H NMR spectrum of 2h.

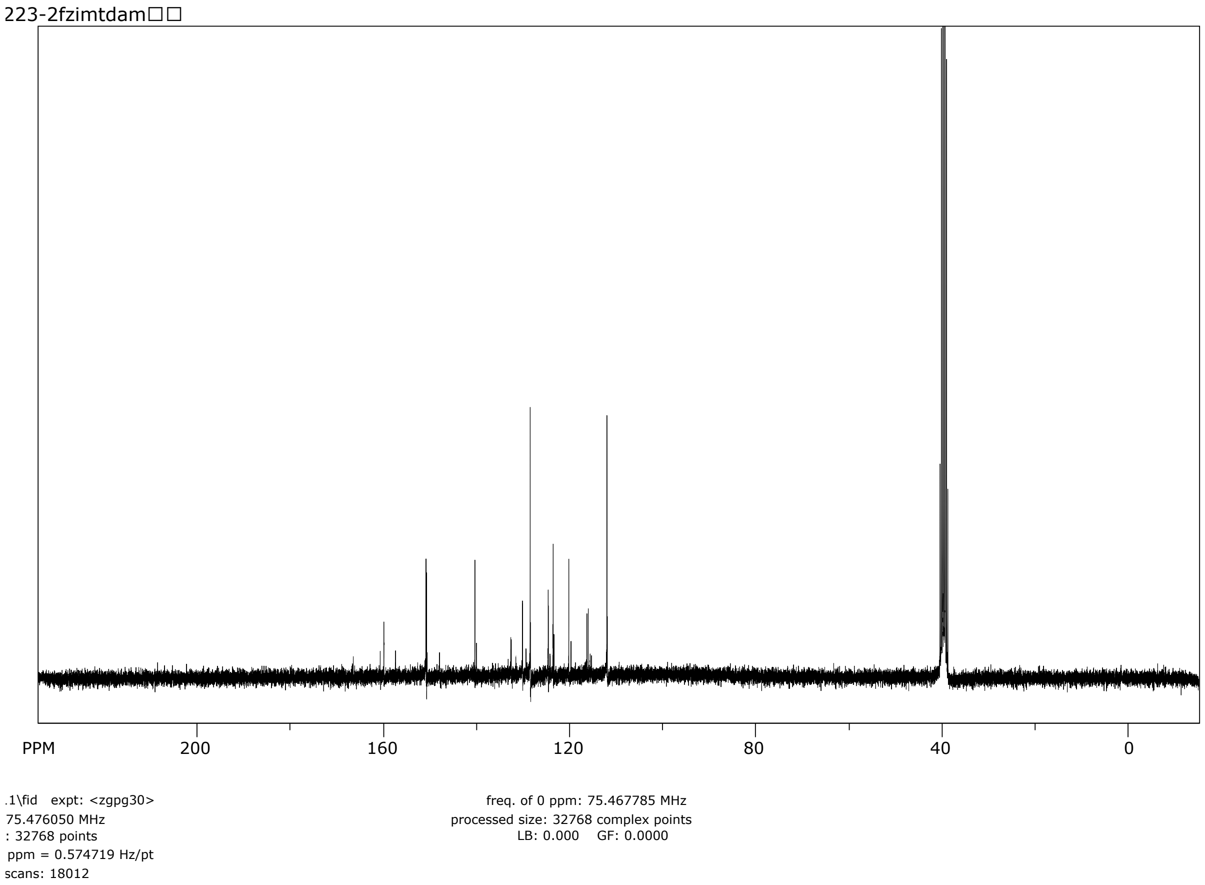

Figure S15. ^{13}C NMR spectrum of 2h.

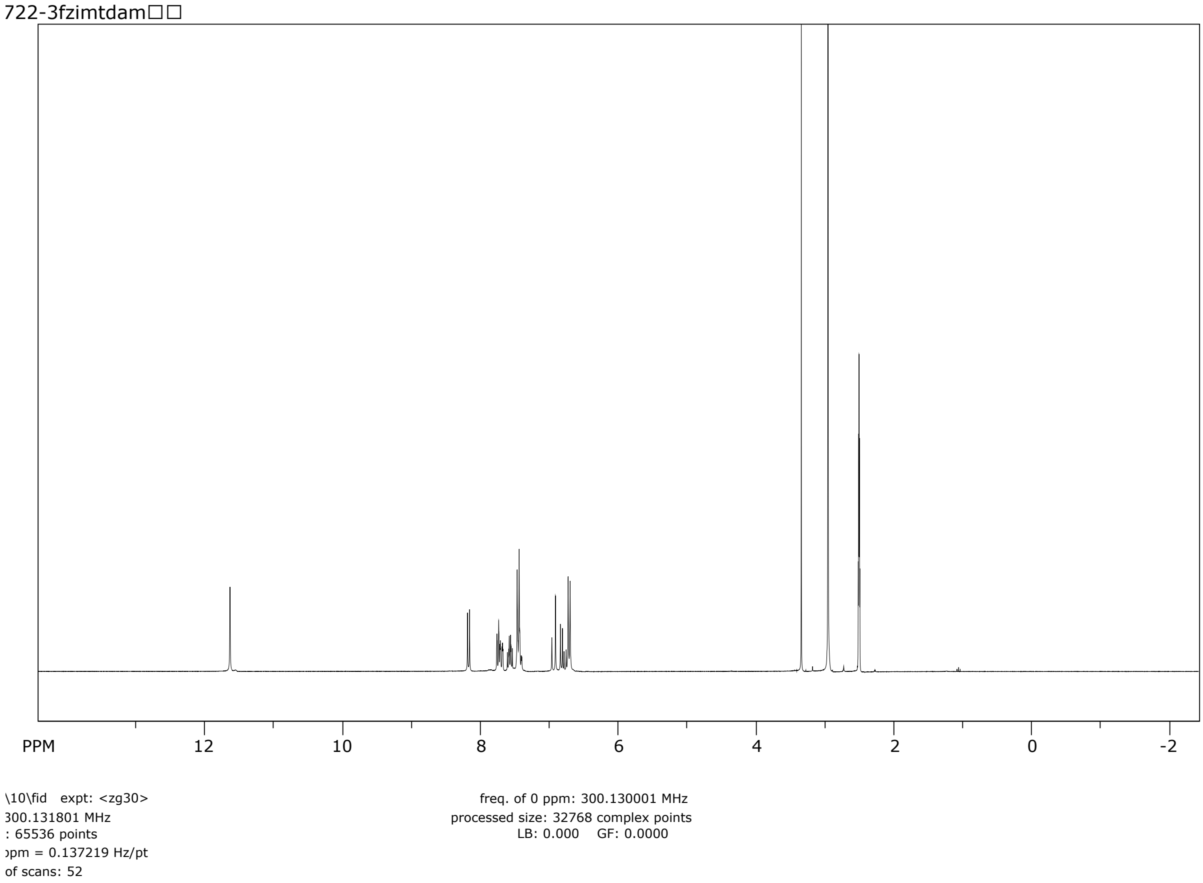

Figure S16. ^{1}**H NMR spectrum of 2j.**

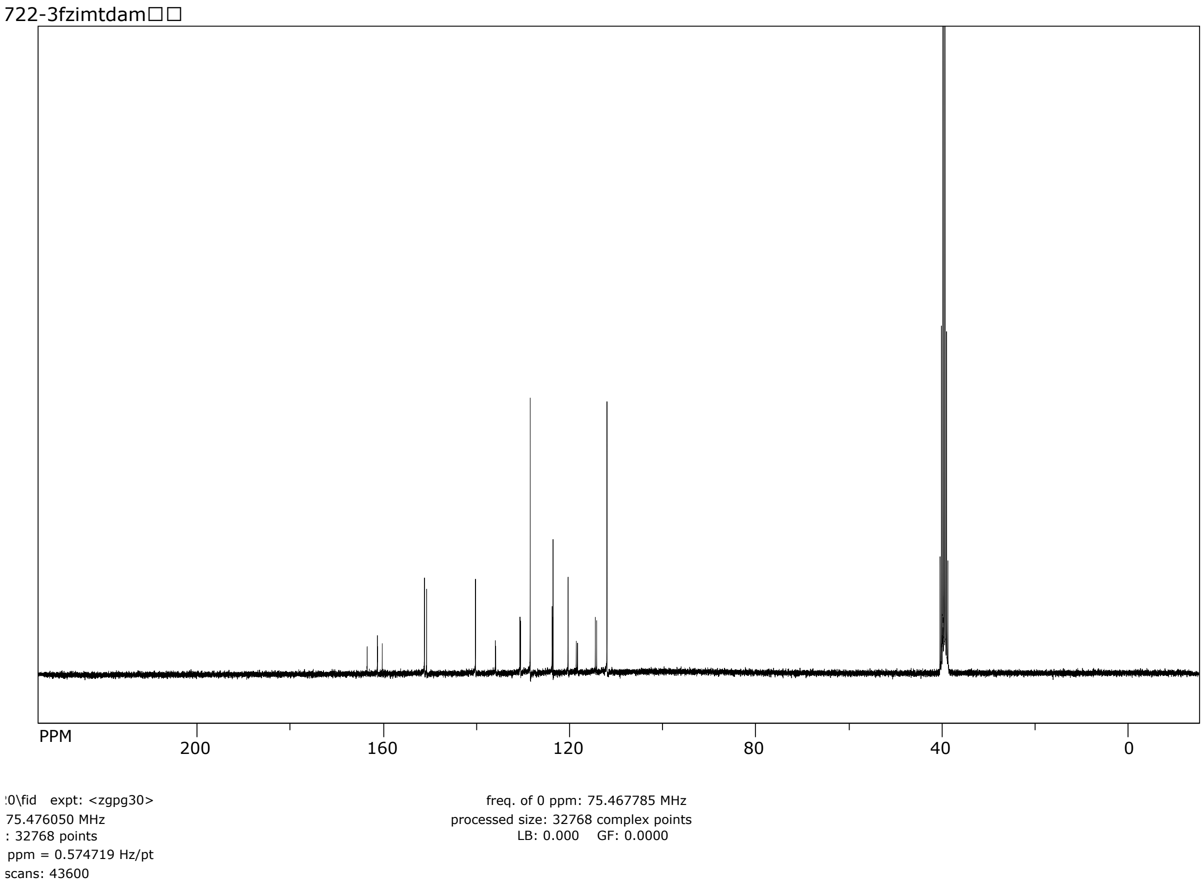

Figure S17. ^{13}C NMR spectrum of 2j.

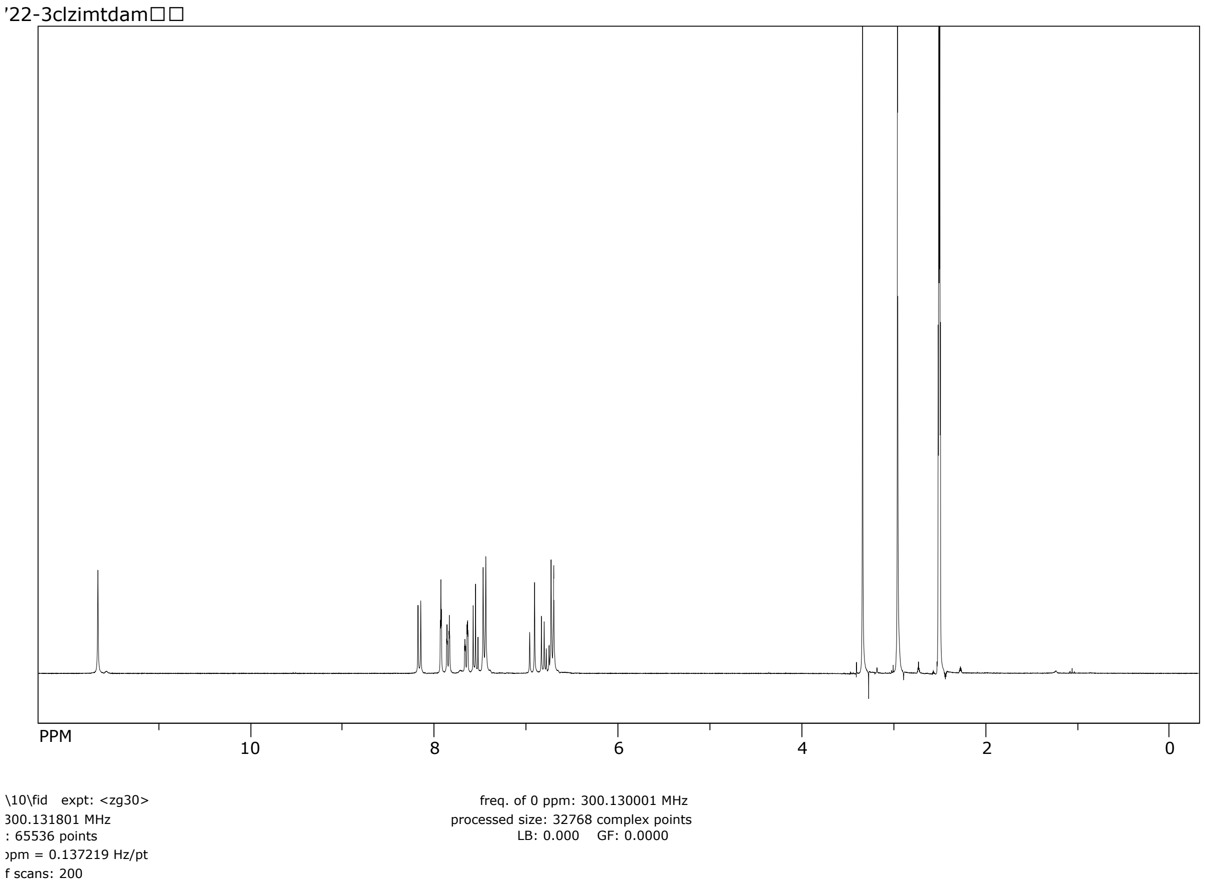

Figure S18. ^{1}H NMR spectrum of 2k.

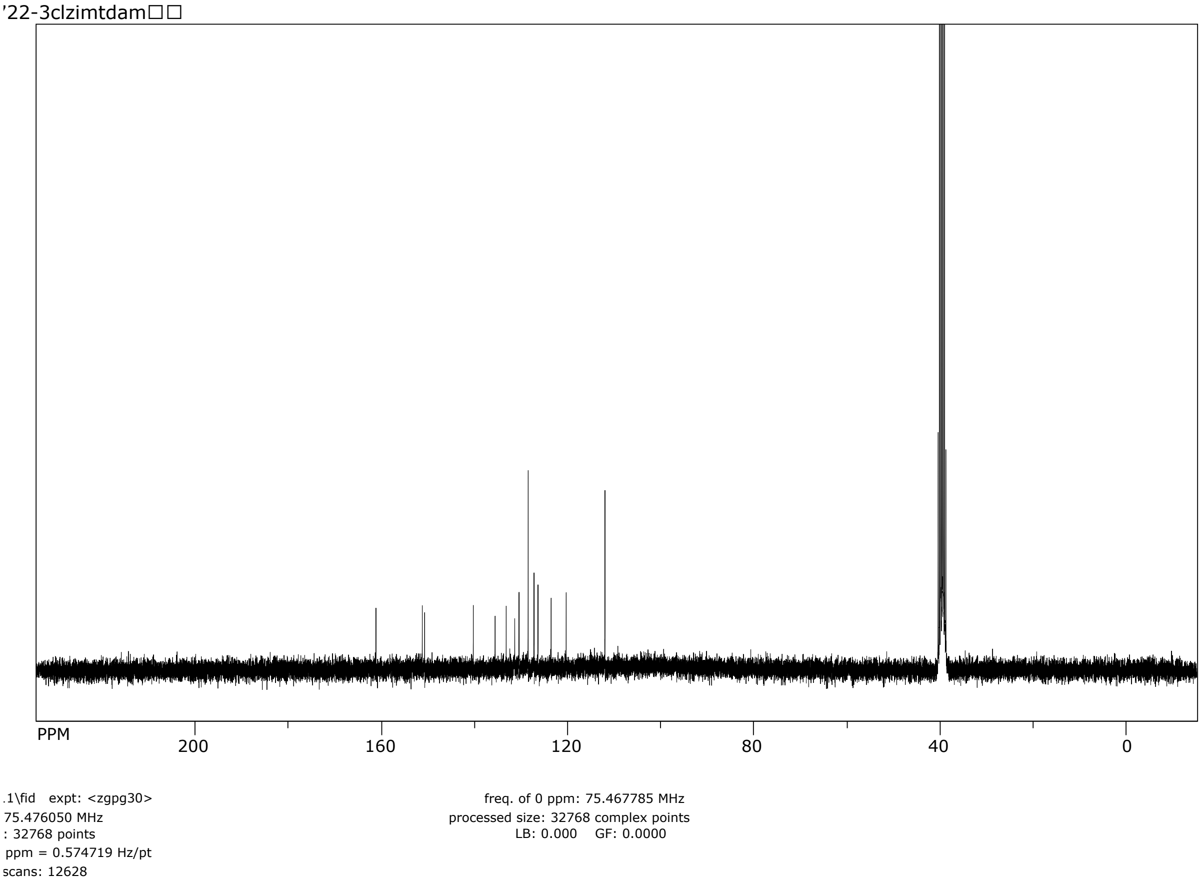

Figure S19. ^{13}C NMR spectrum of 2k.

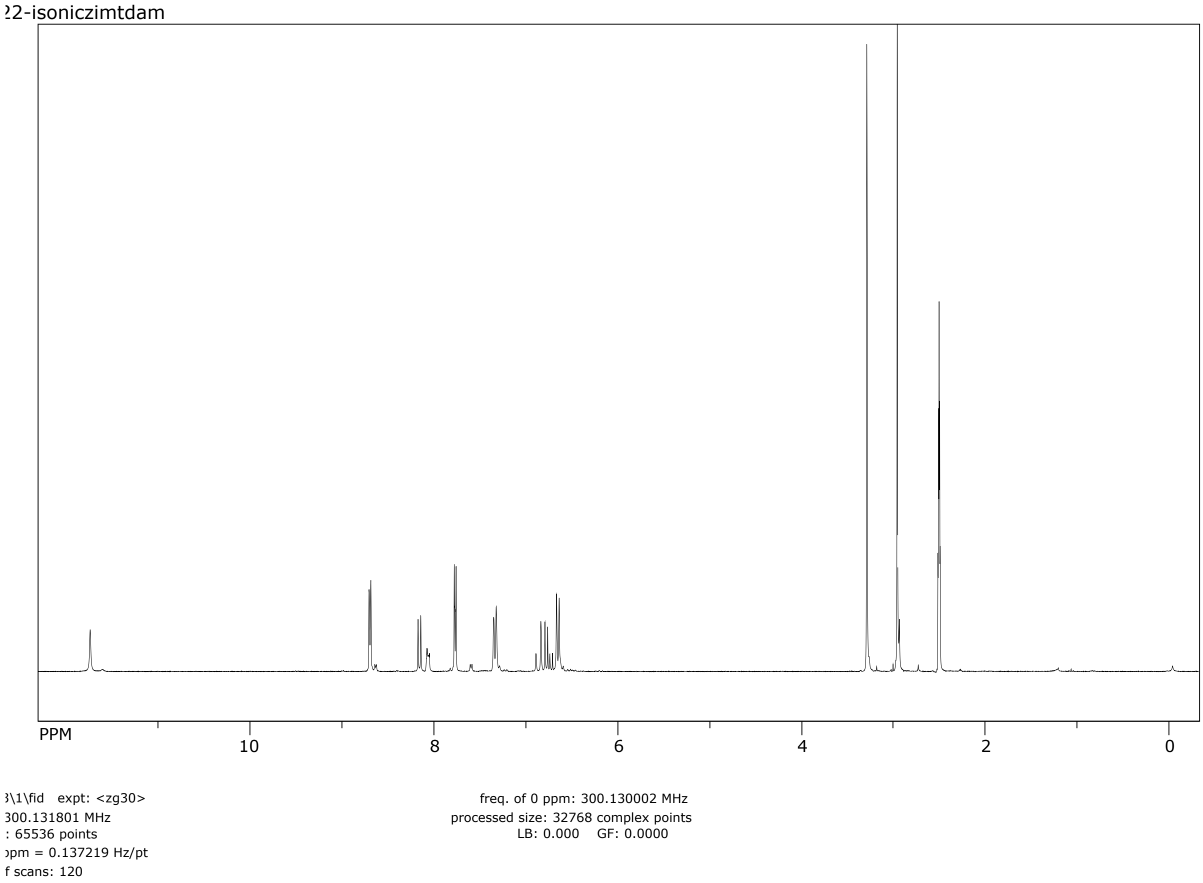

Figure S20. ^{1}H NMR spectrum of 2m.

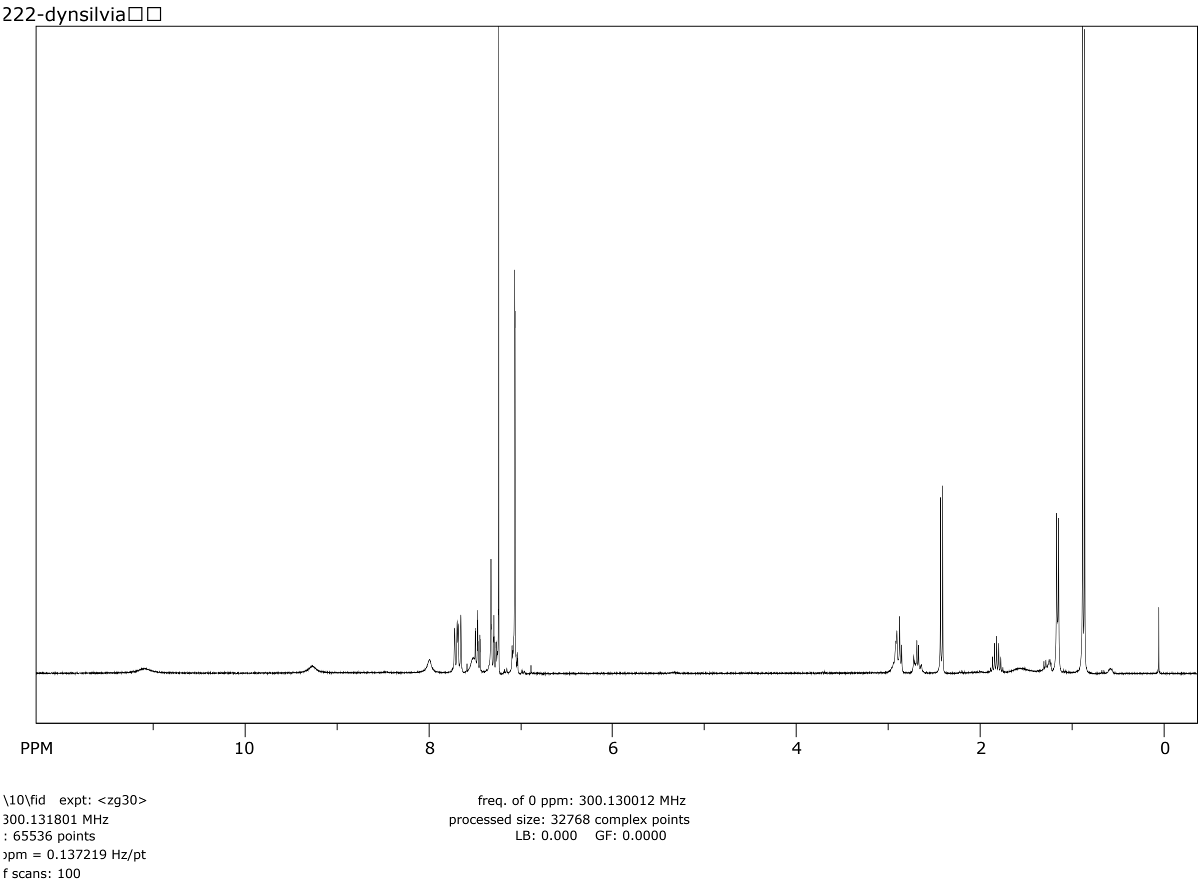

Figure S21. ^{1}H NMR spectrum of 3a.

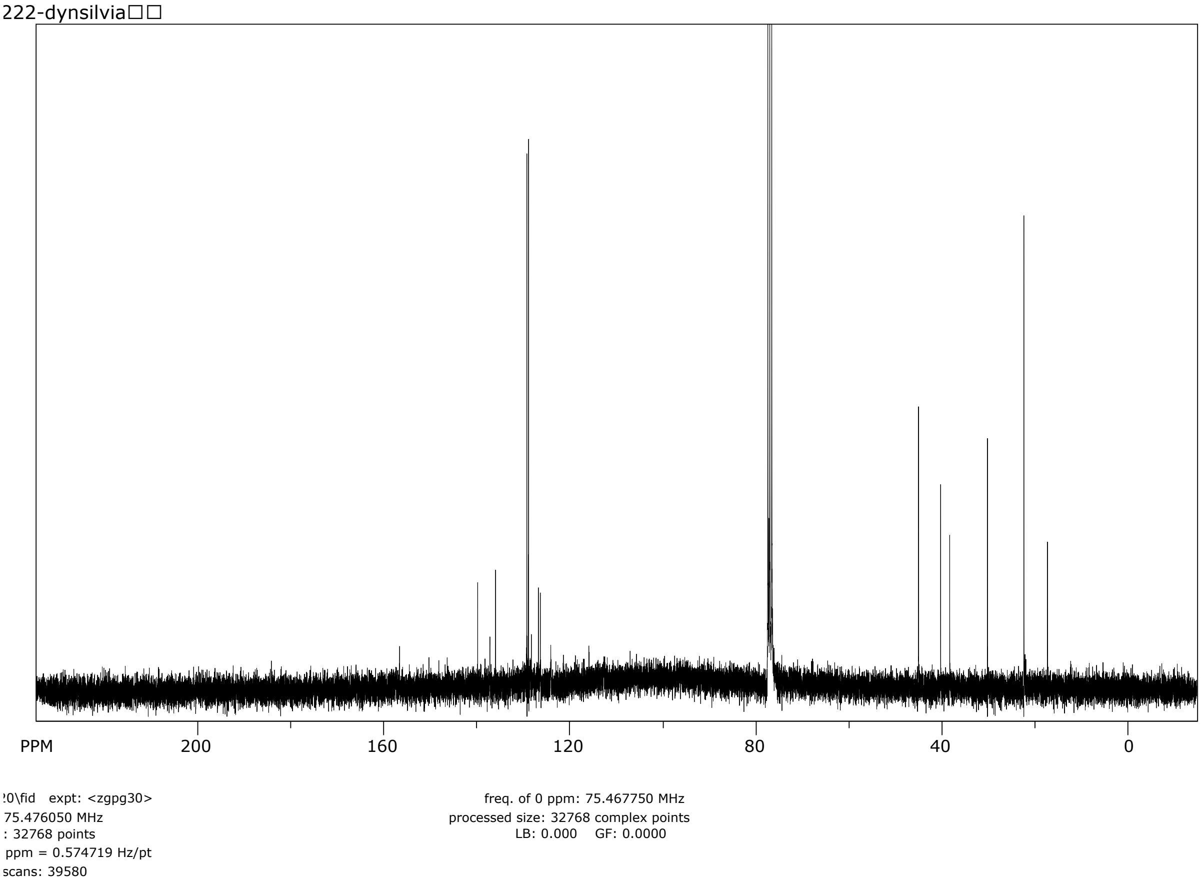

Figure S22. ^{13}C NMR spectrum of 3a.

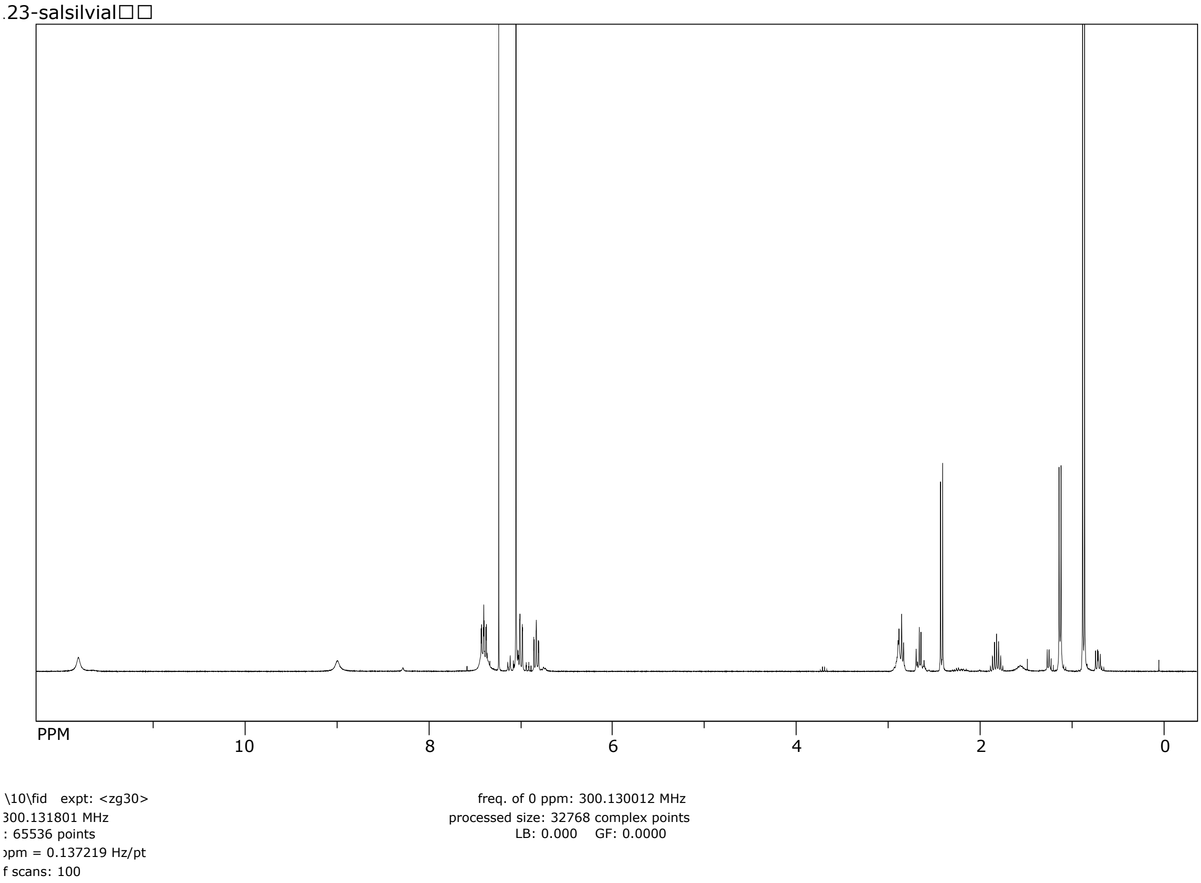

Figure S23. ¹H NMR spectrum of 3b.

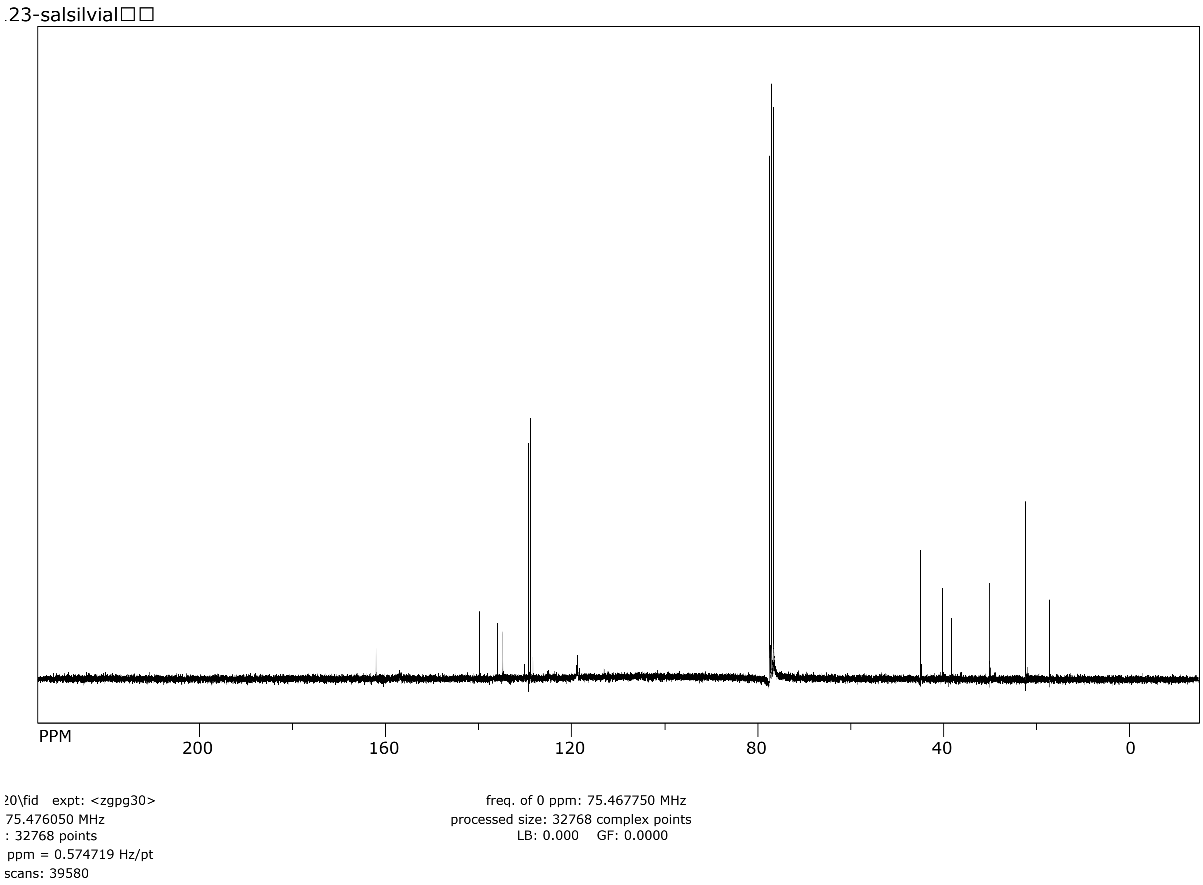

Figure S24. ^{13}C NMR spectrum of 3b.

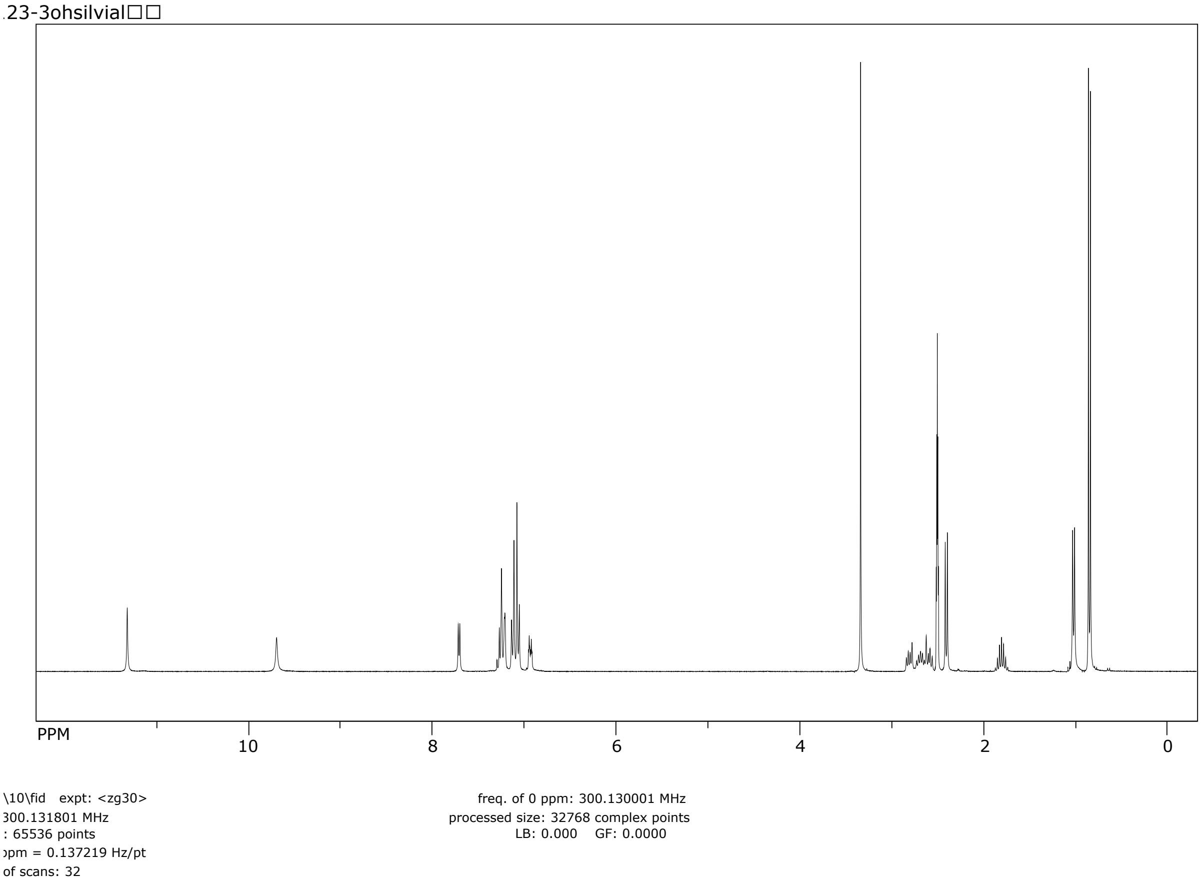

Figure S25. ^{1}H NMR spectrum of 3c.

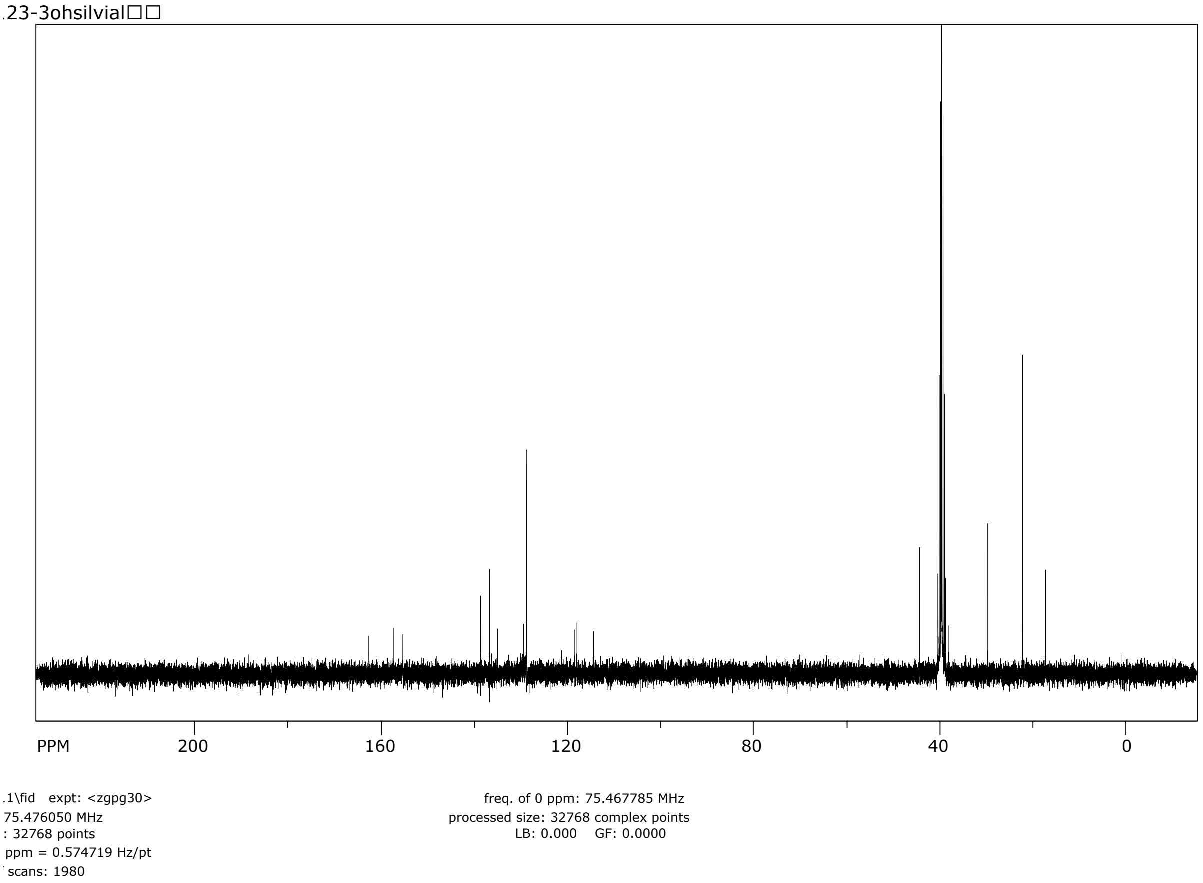

Figure S26. ^{13}C NMR spectrum of 3c.

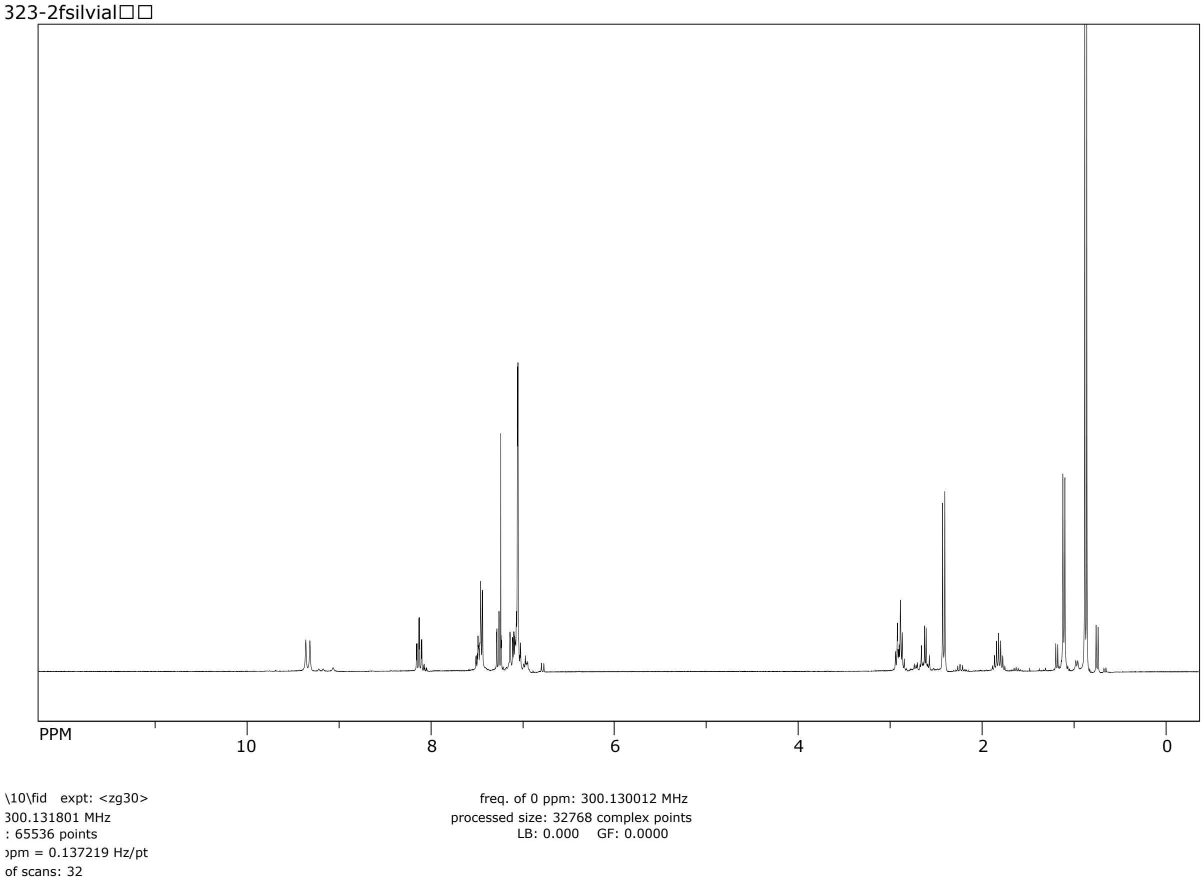

Figure S27. ^{1}H NMR spectrum of 3d.

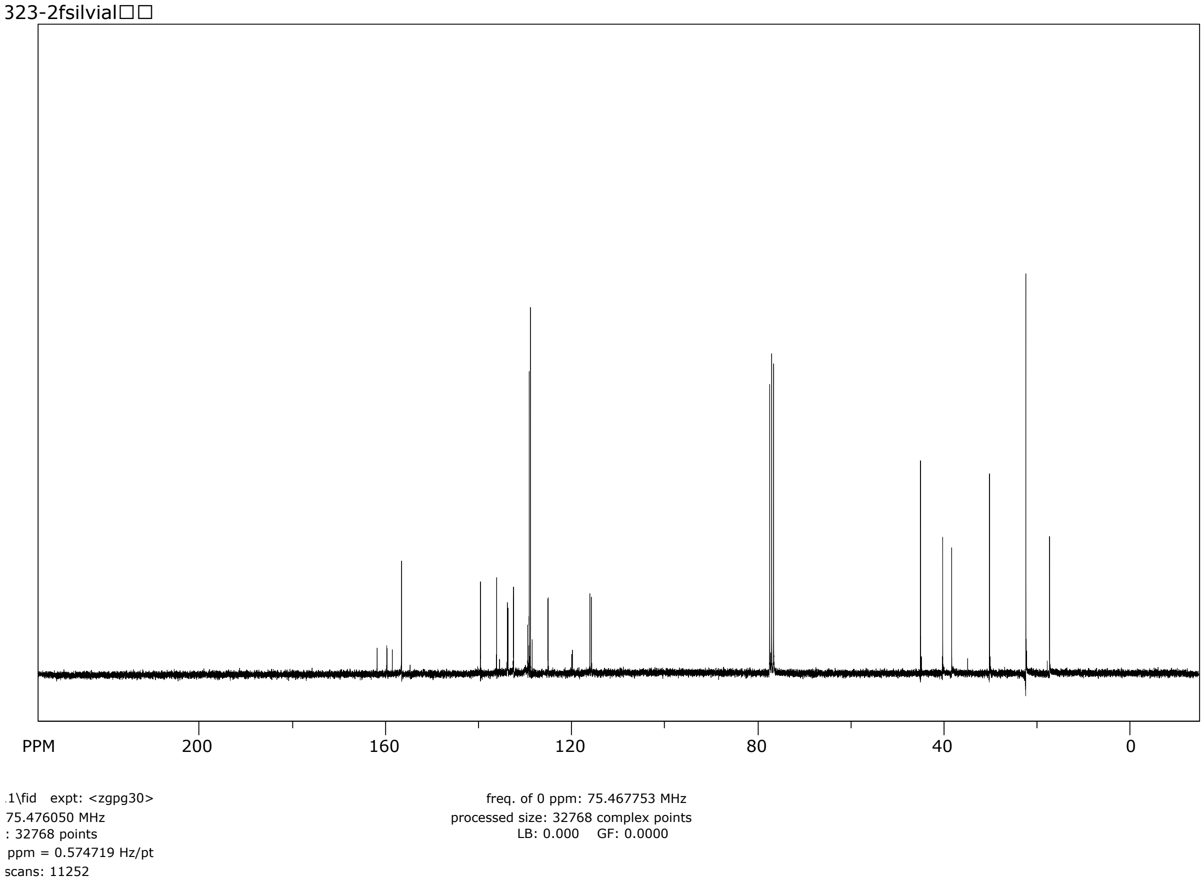

Figure S28. ^{13}C NMR spectrum of 3d.

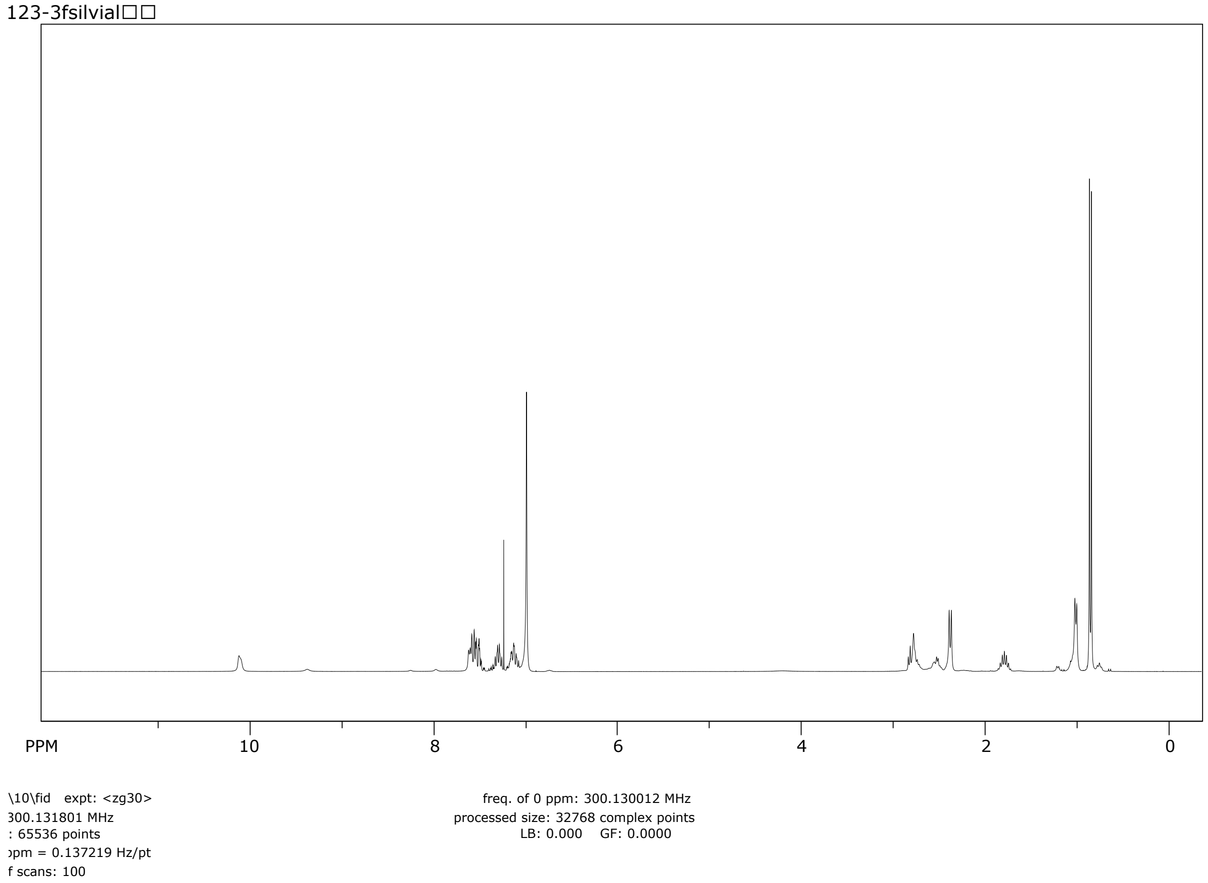

Figure S29. ^{1}H NMR spectrum of 3e.

123-3fsilvial□□

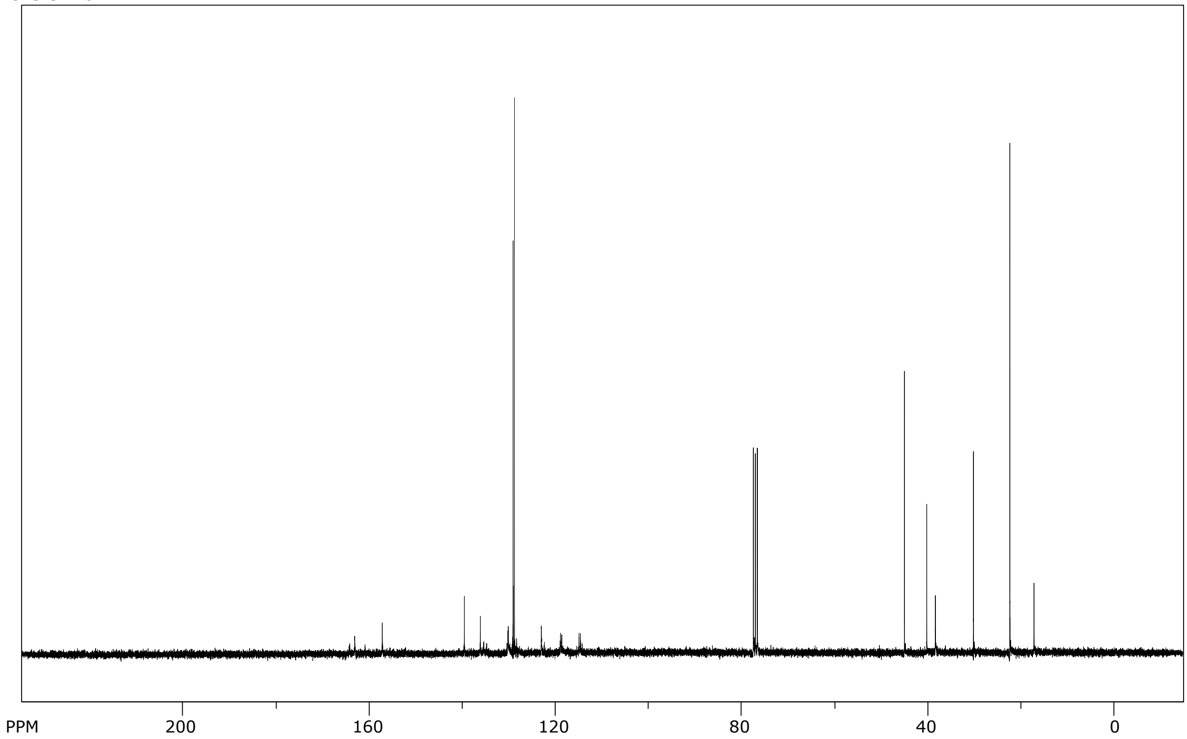

.1\fid expt: <zgpg30>
75.476050 MHz
: 32768 points
ppm = 0.574719 Hz/pt
scans: 4200

freq. of 0 ppm: 75.467754 MHz
processed size: 32768 complex points
LB: 0.000 GF: 0.0000

Figure S30. ^{13}C NMR spectrum of 3e.

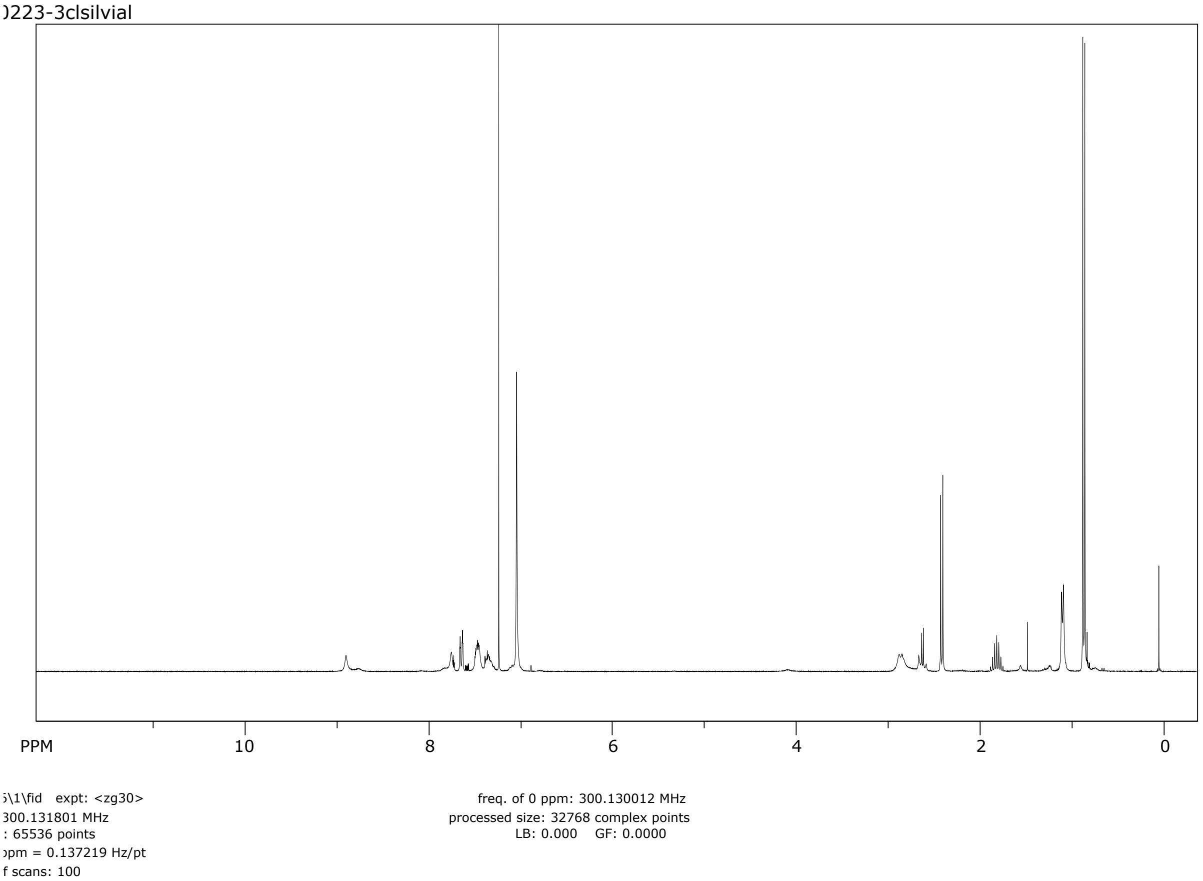

Figure S31. ^{1}H NMR spectrum of 3f.

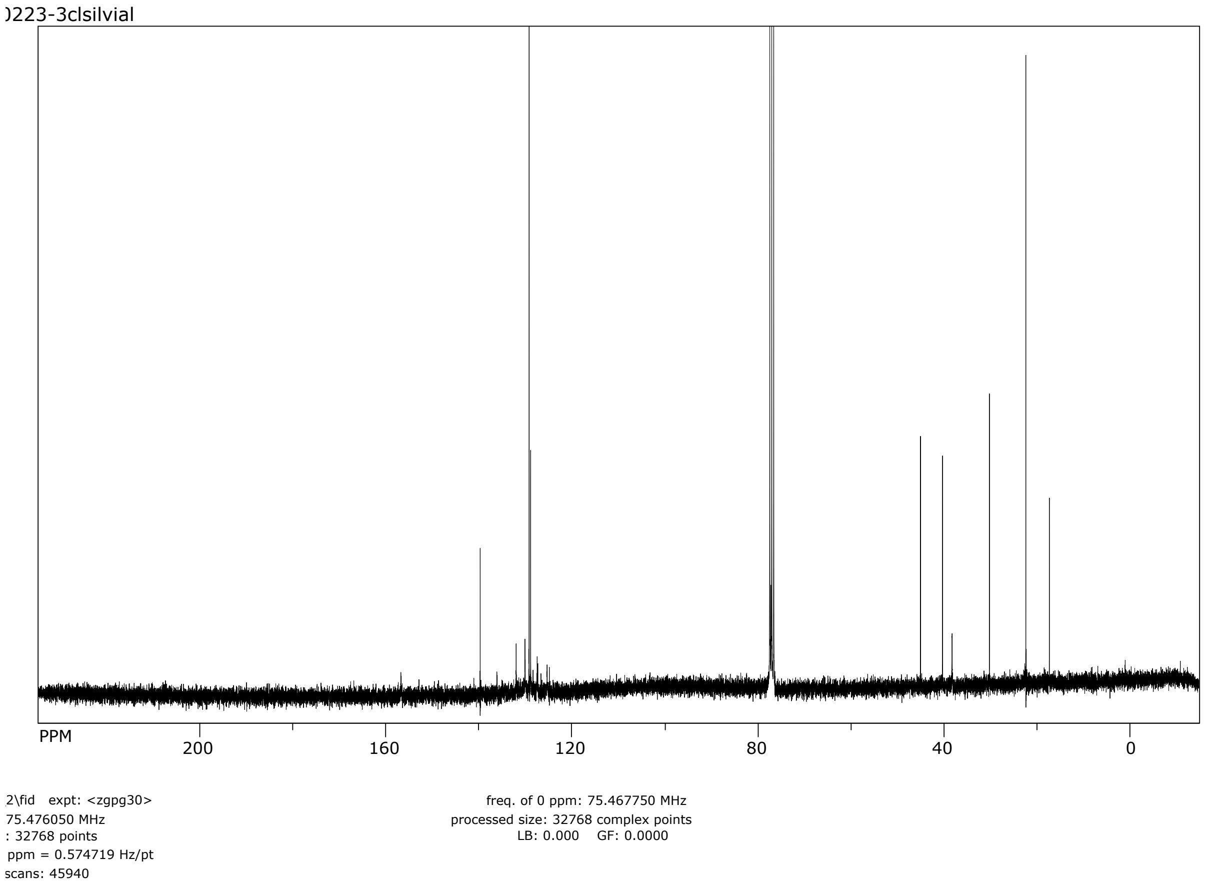

Figure S32. ^{13}C NMR spectrum of 3f.

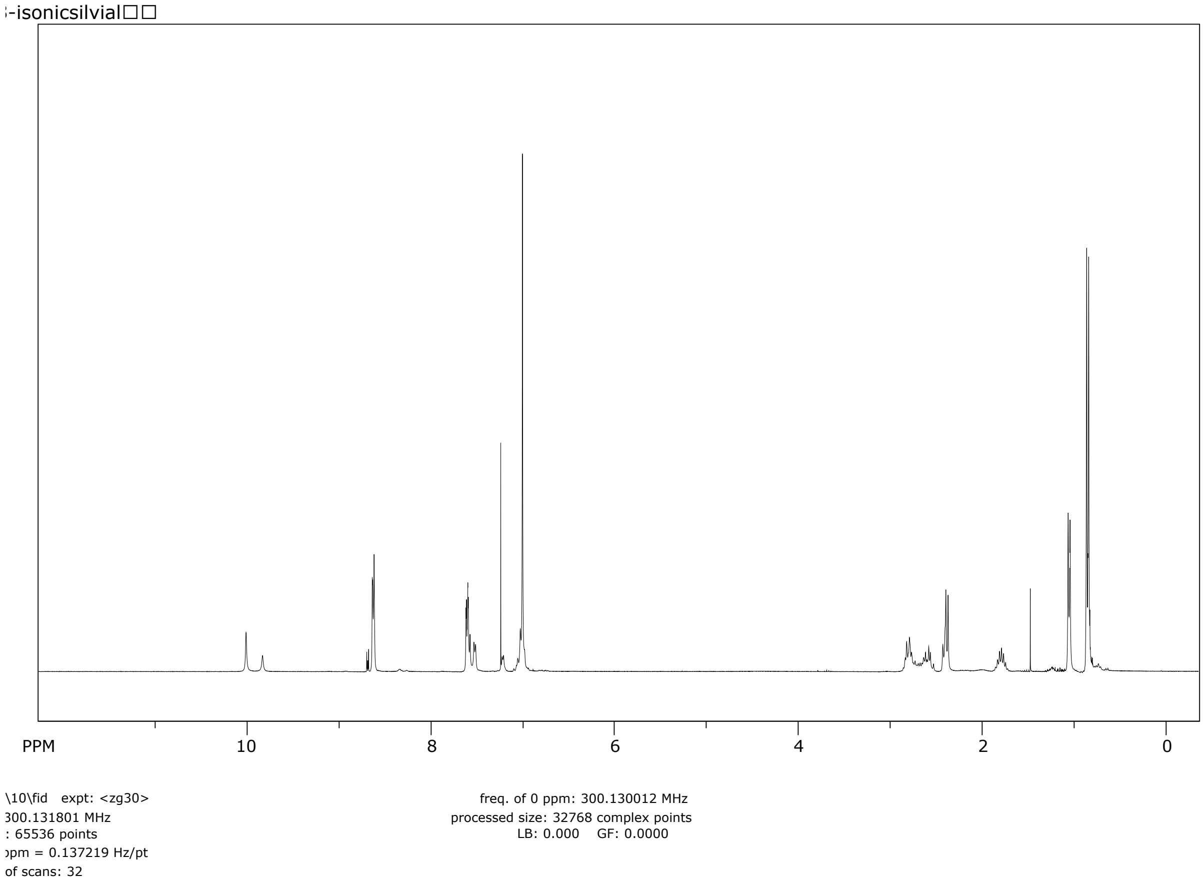

Figure S33. ^{1}H NMR spectrum of 3g.

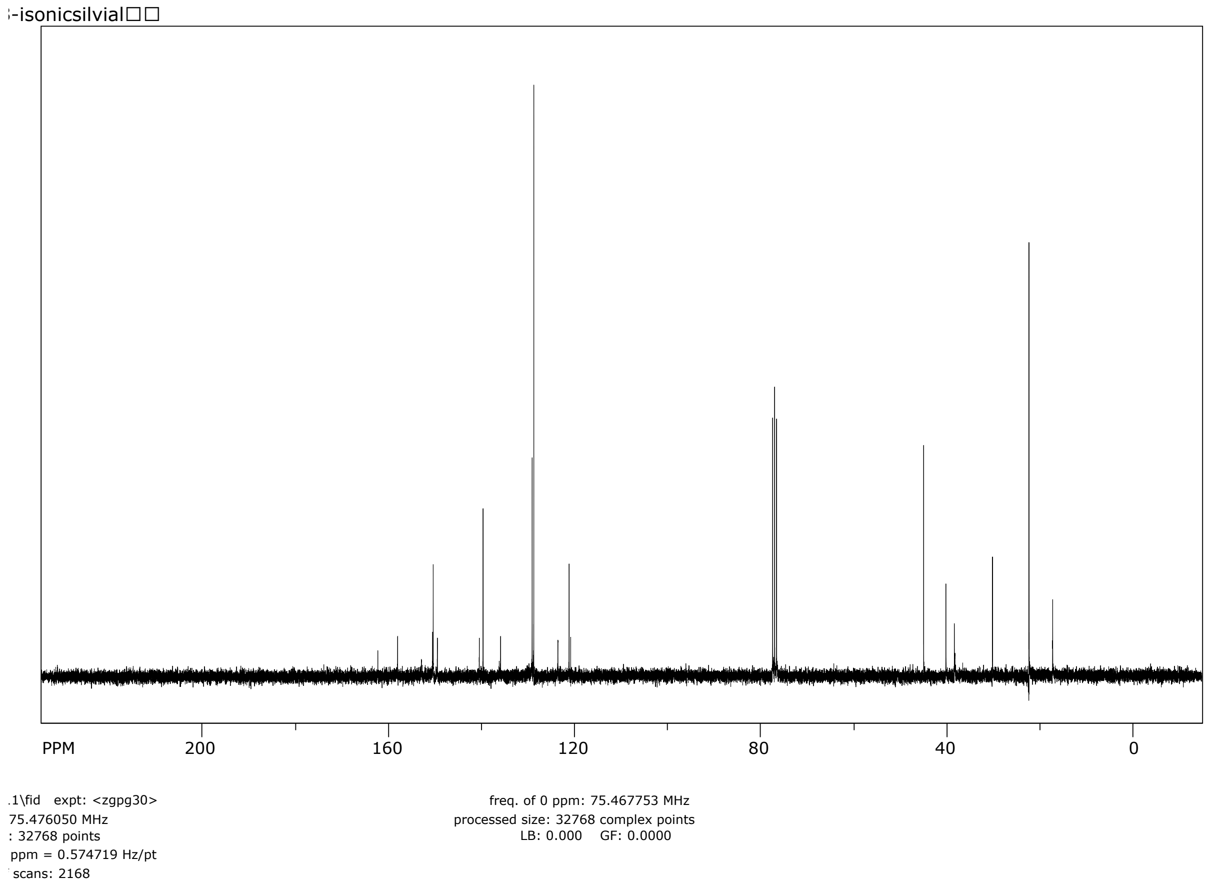

Figure S34. ^{13}C NMR spectrum of 3g.

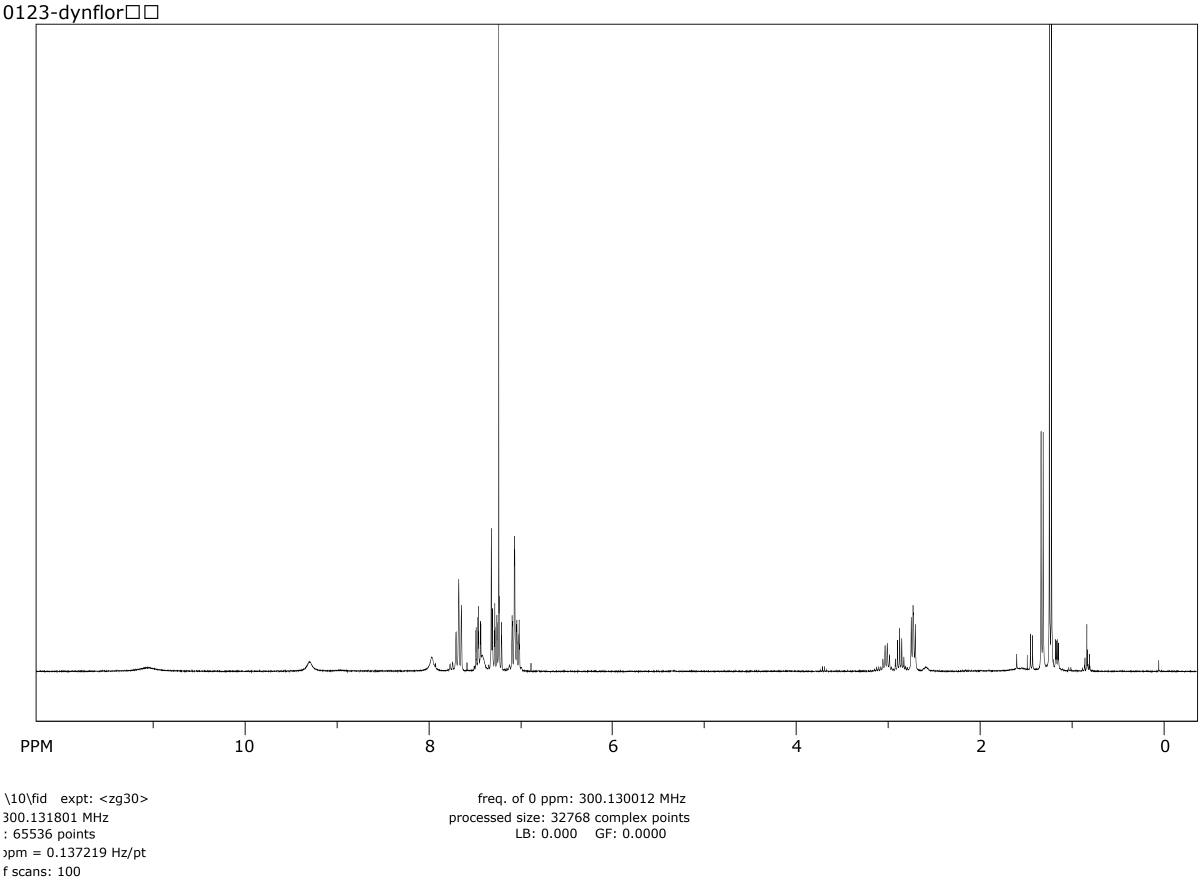

Figure S35. ^{1}H NMR spectrum of 4a.

0123-dynflor□□

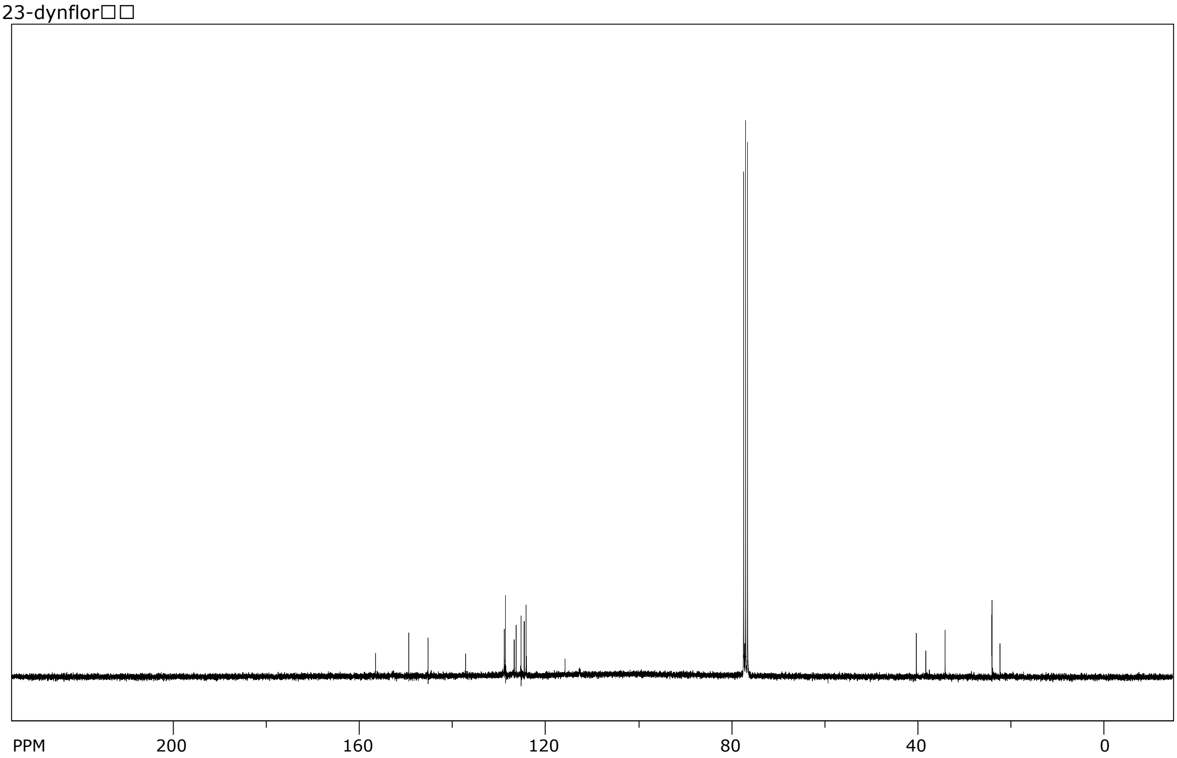

20\fid expt: <zgpg30>
75.476050 MHz
: 32768 points
ppm = 0.574719 Hz/pt
scans: 56484

freq. of 0 ppm: 75.467750 MHz
processed size: 32768 complex points
LB: 0.000 GF: 0.0000

Figure S36. ^{13}C NMR spectrum of 4a.

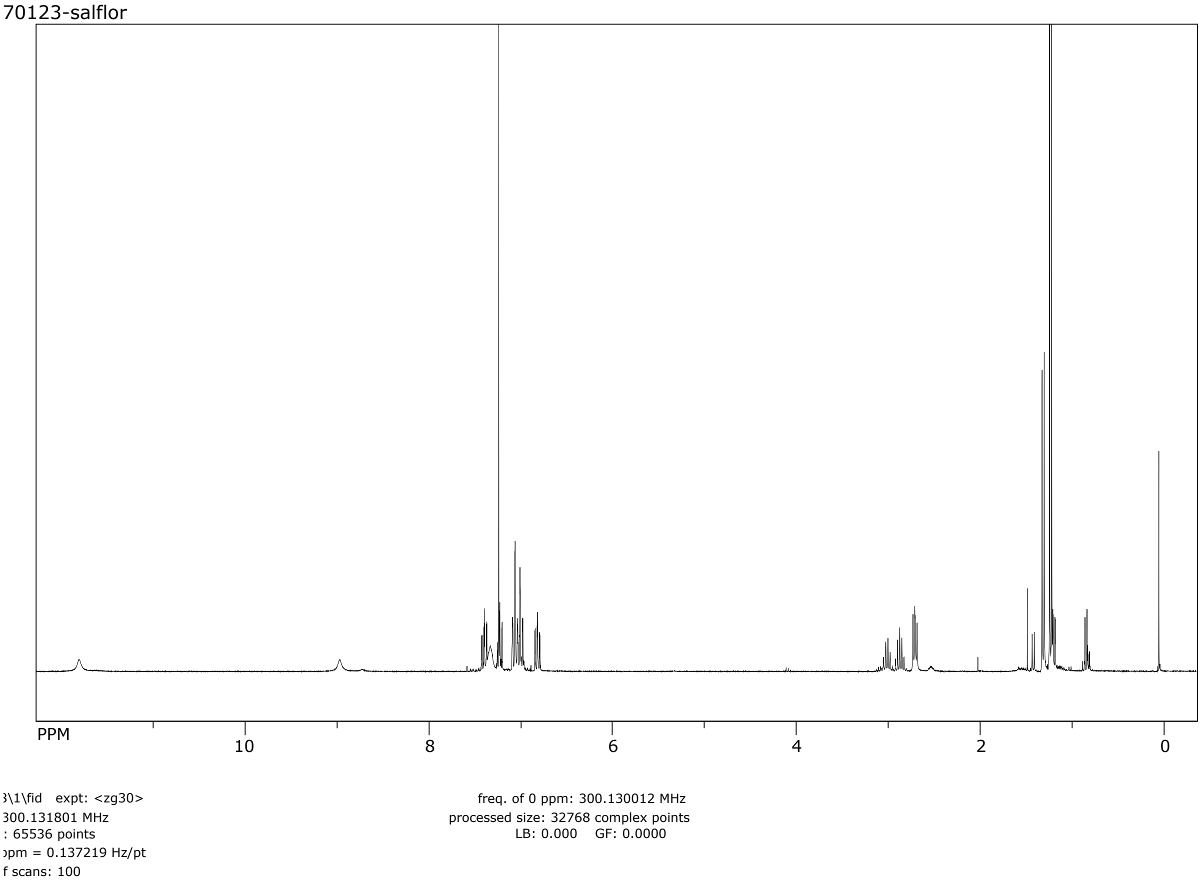

Figure S37. ^{1}H NMR spectrum of 4b.

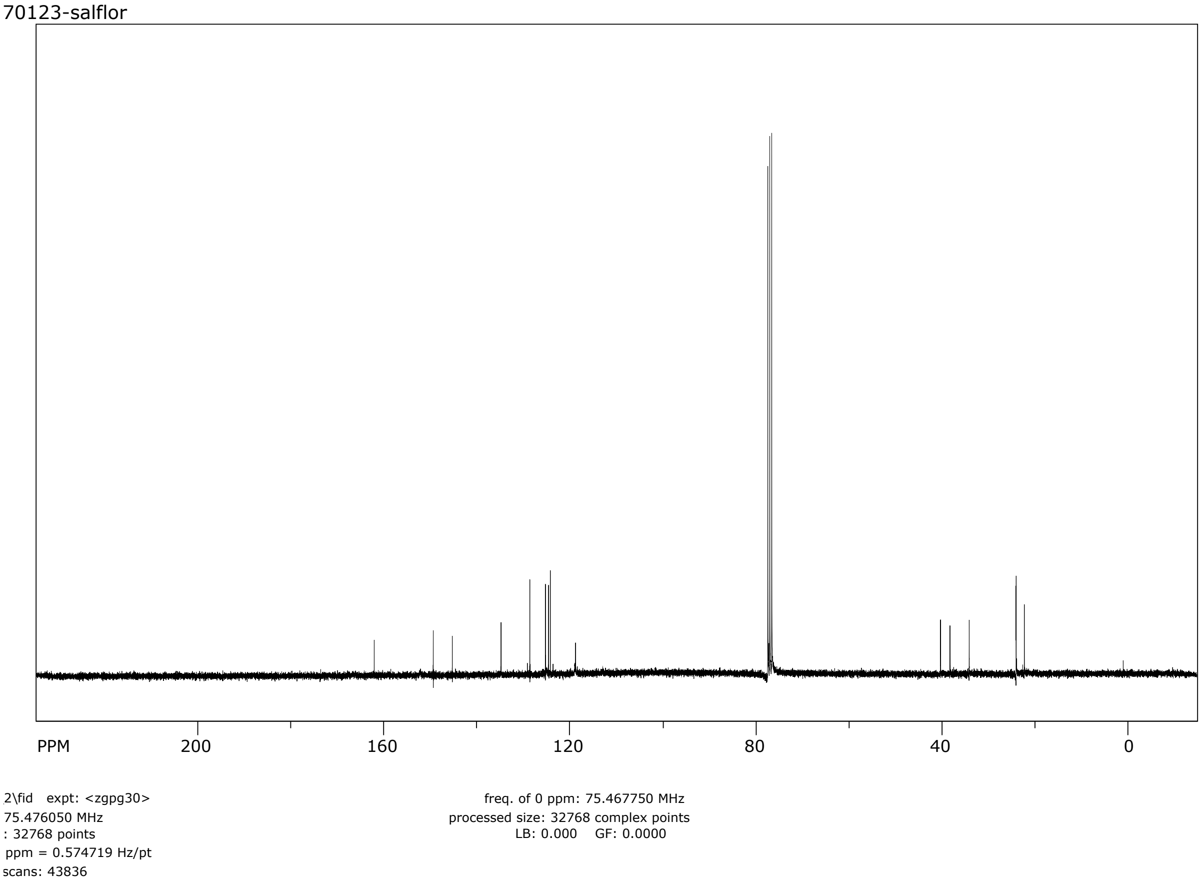

Figure S38. ^{13}C NMR spectrum of 4b.

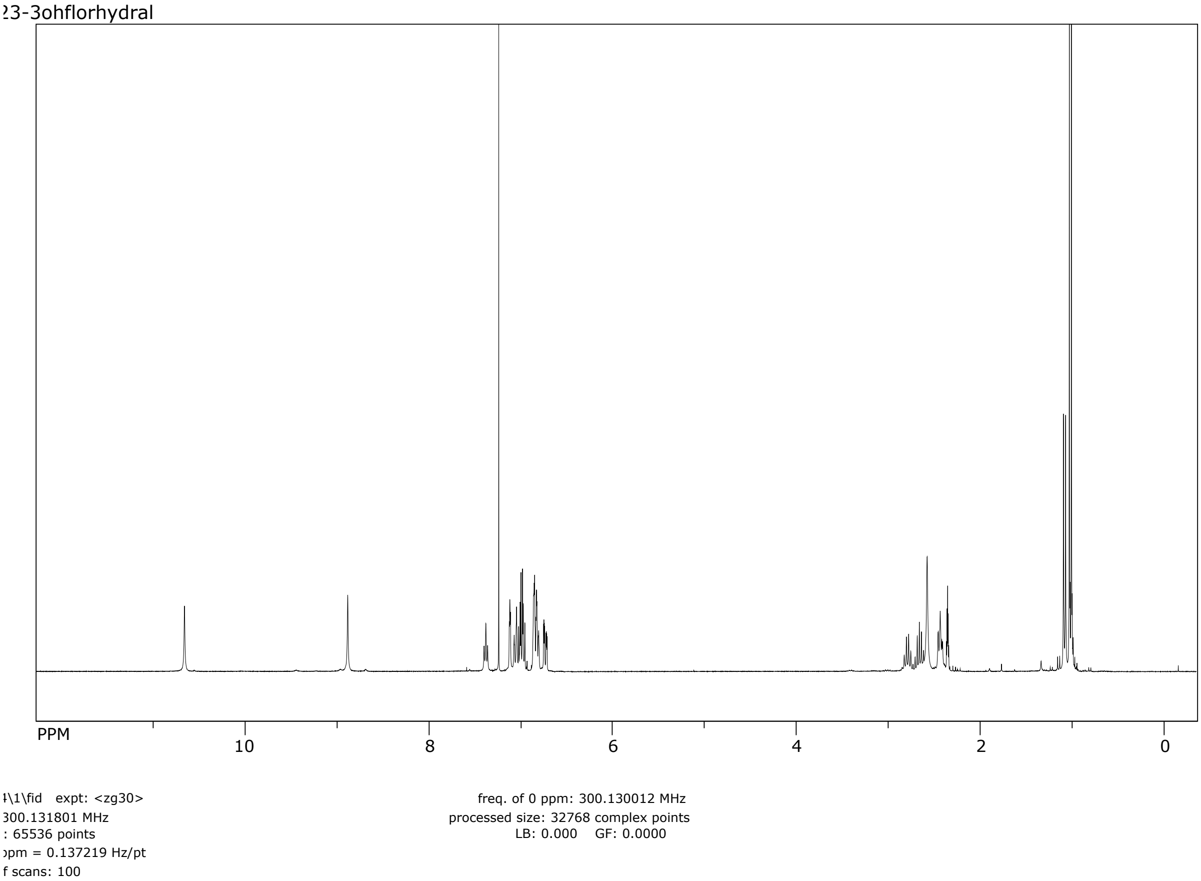

Figure S39. ^1H NMR spectrum of 4c.

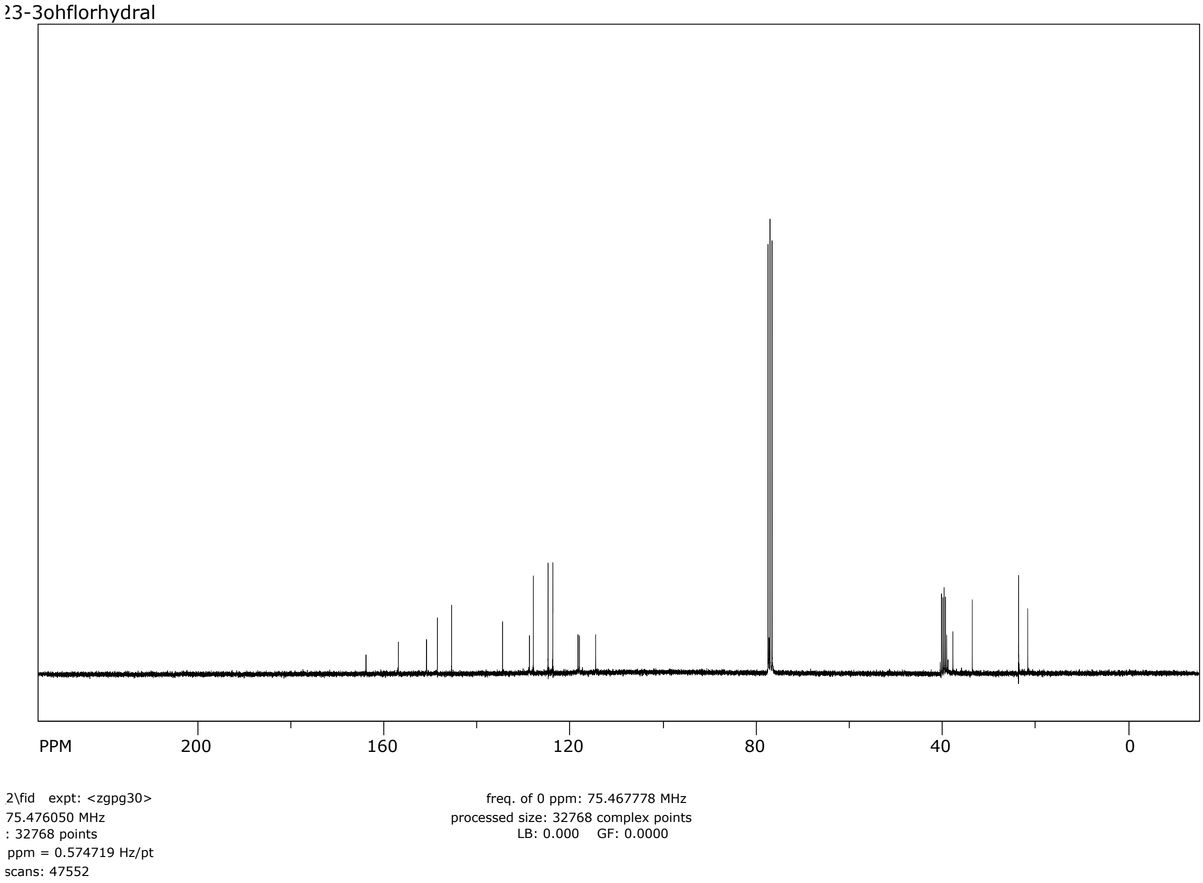

Figure S40. ^{13}C NMR spectrum of 4c.

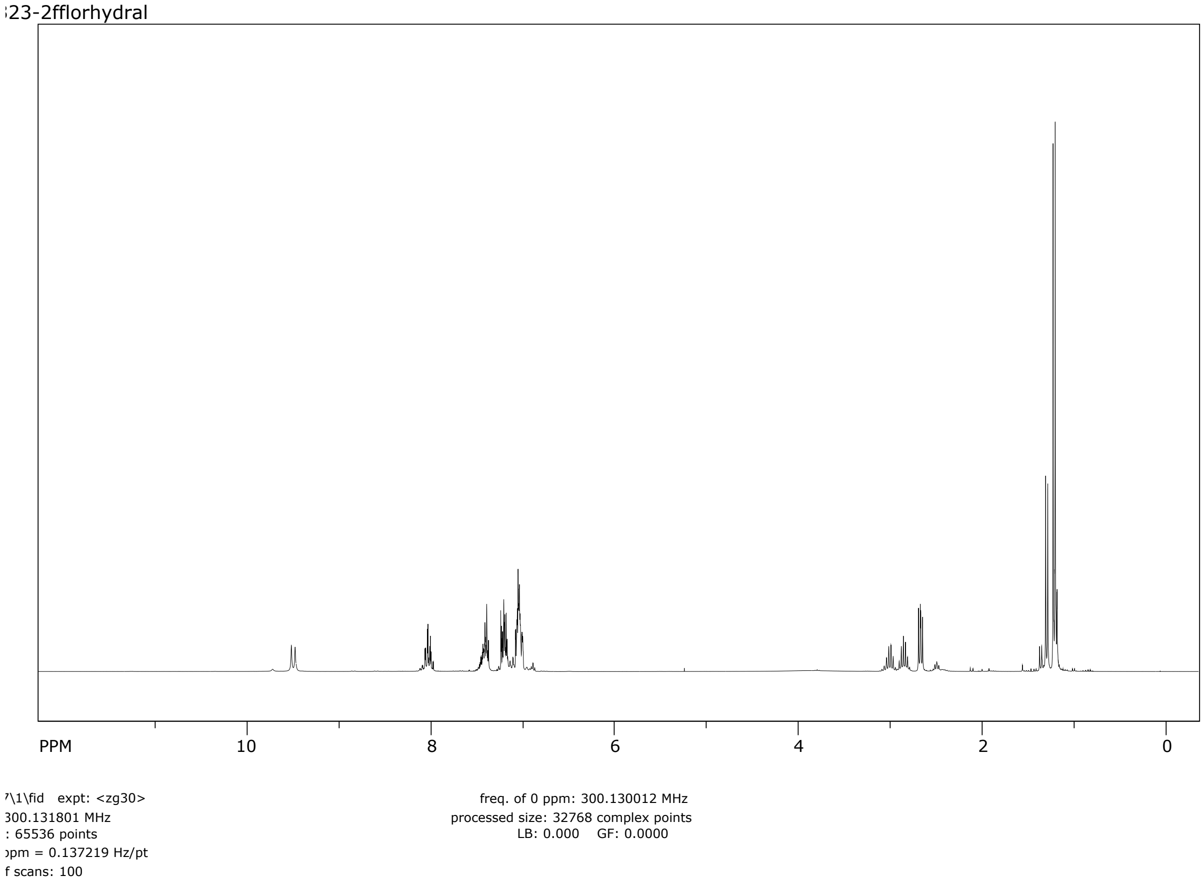

Figure S41. ^{1}H NMR spectrum of 4d.

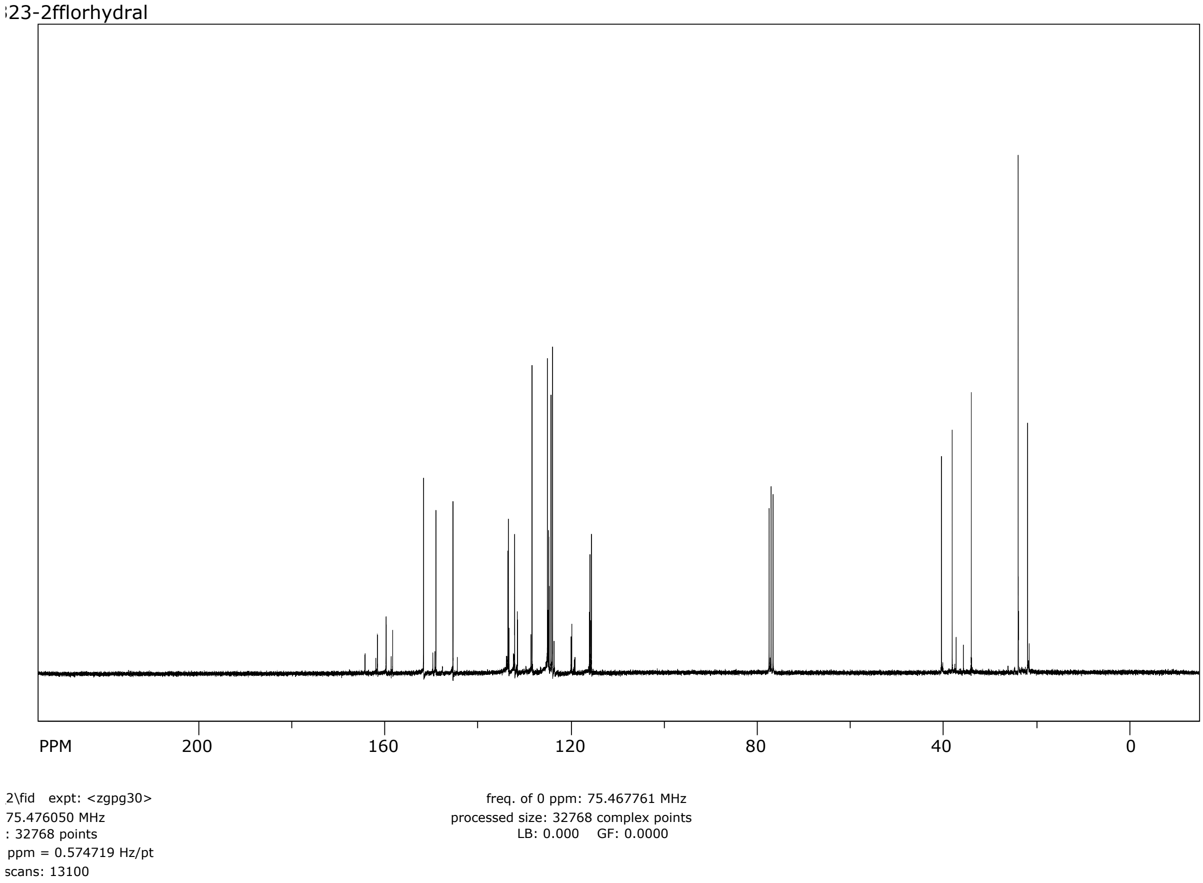

Figure S42. ^{13}C NMR spectrum of 4d.

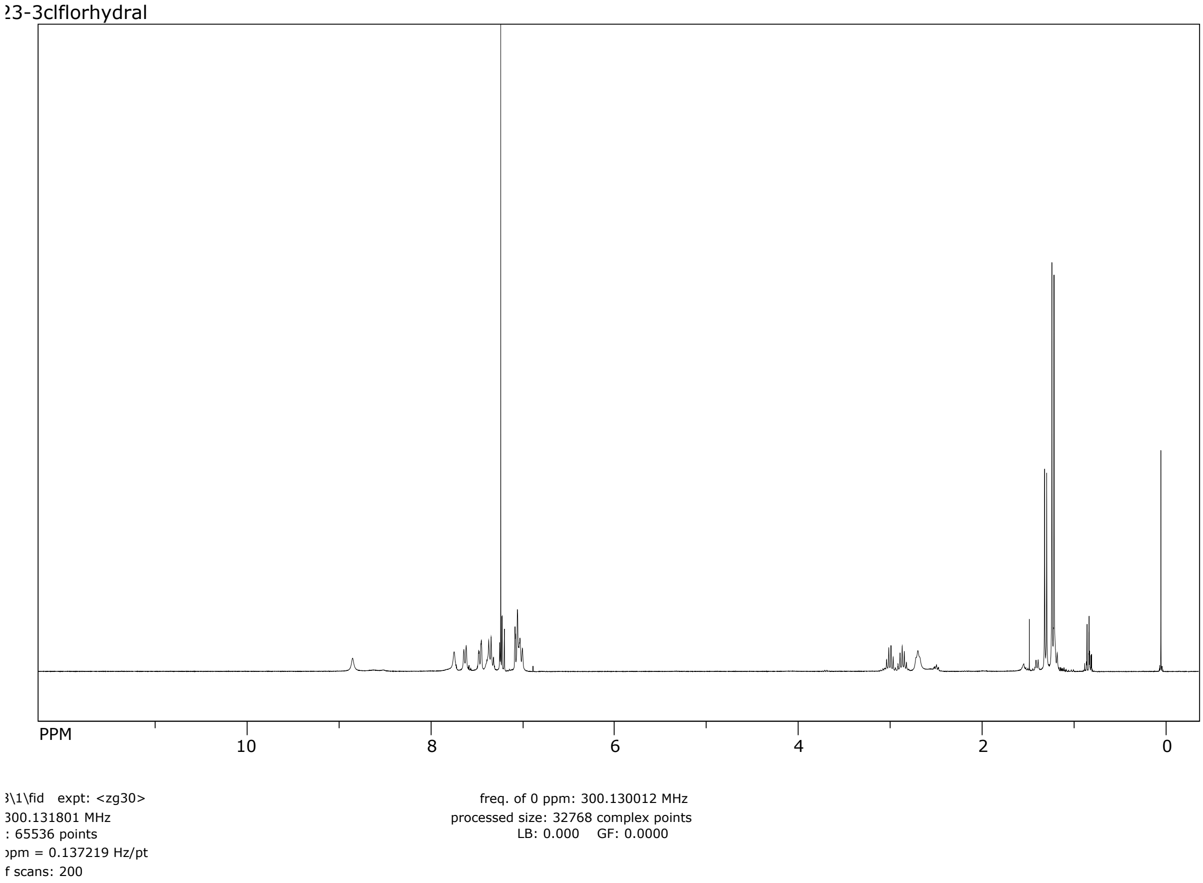

Figure S43. ^{1}H NMR spectrum of 4f.

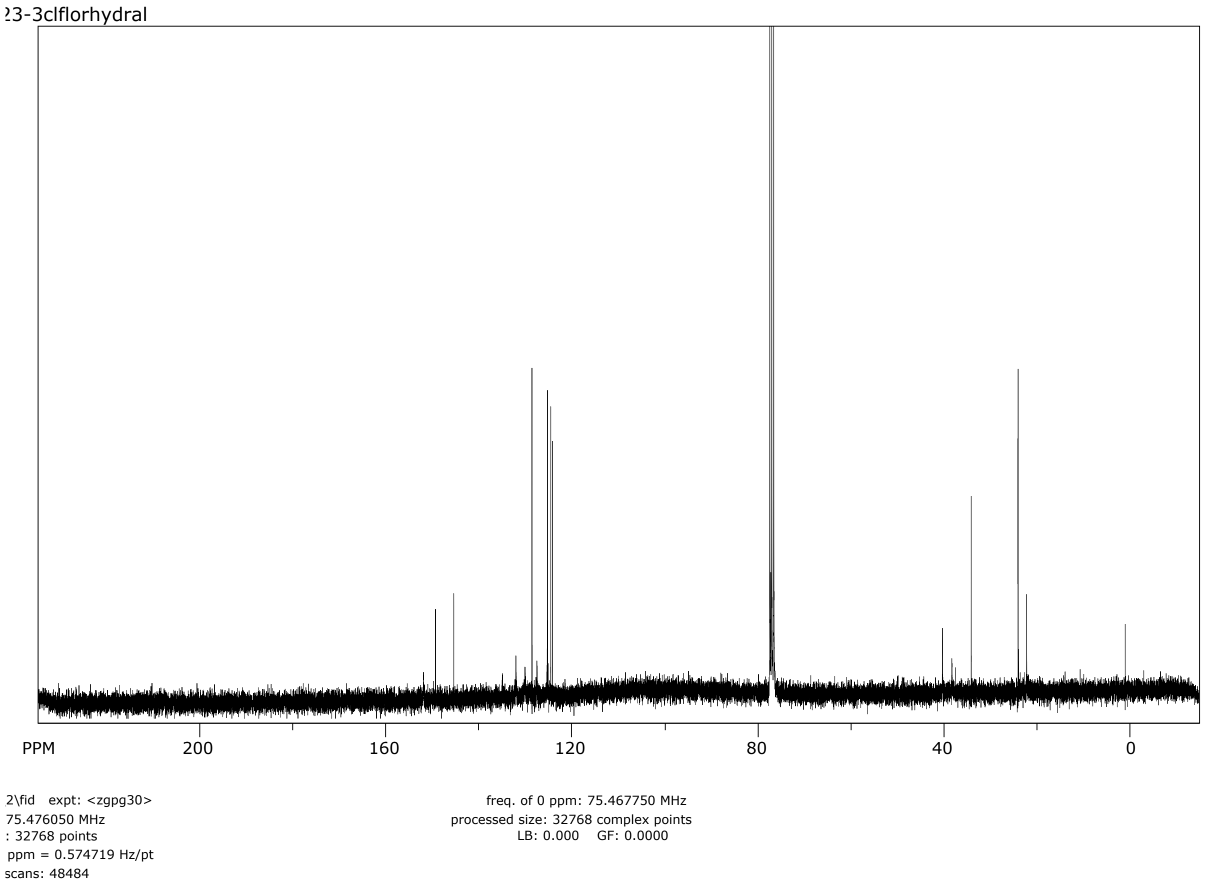

Figure S44. ^{13}C **NMR spectrum of 4f.**

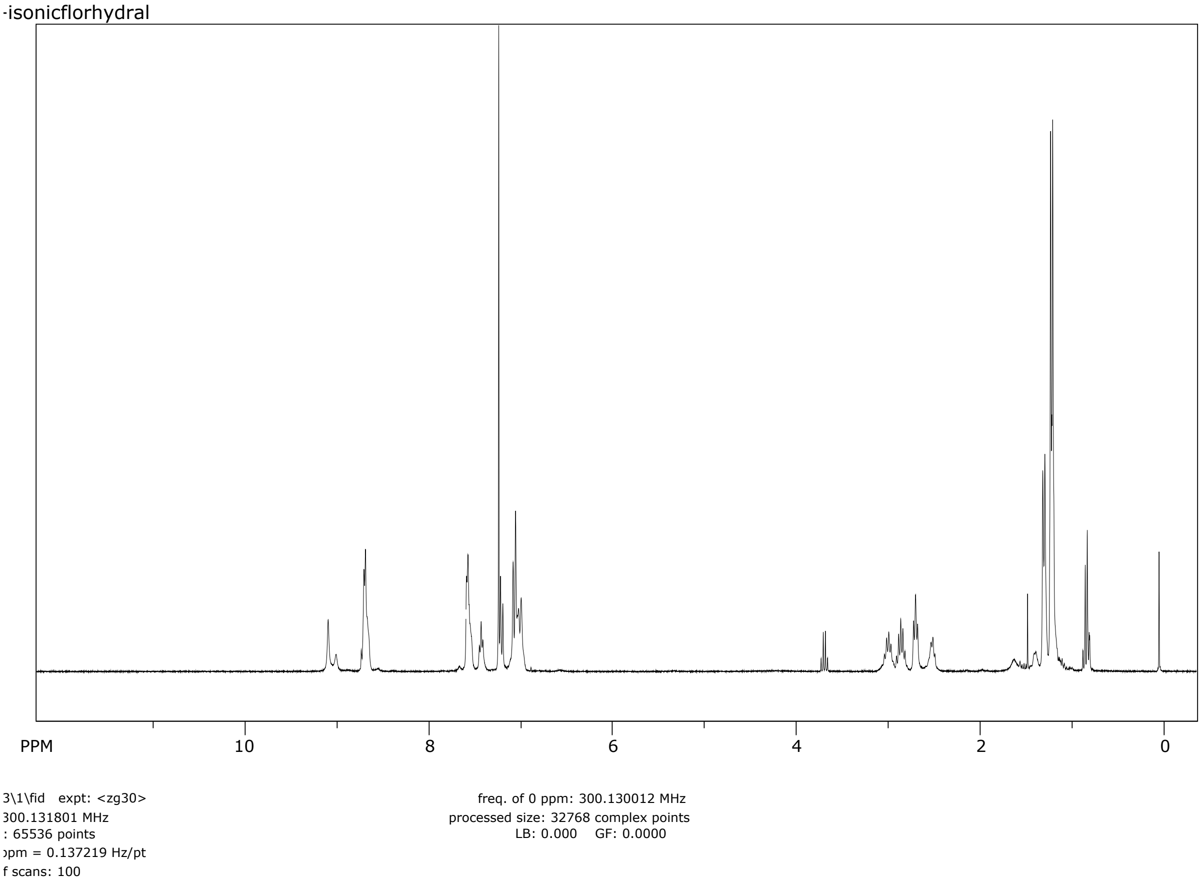

Figure S45. ^{1}H NMR spectrum of 4g.

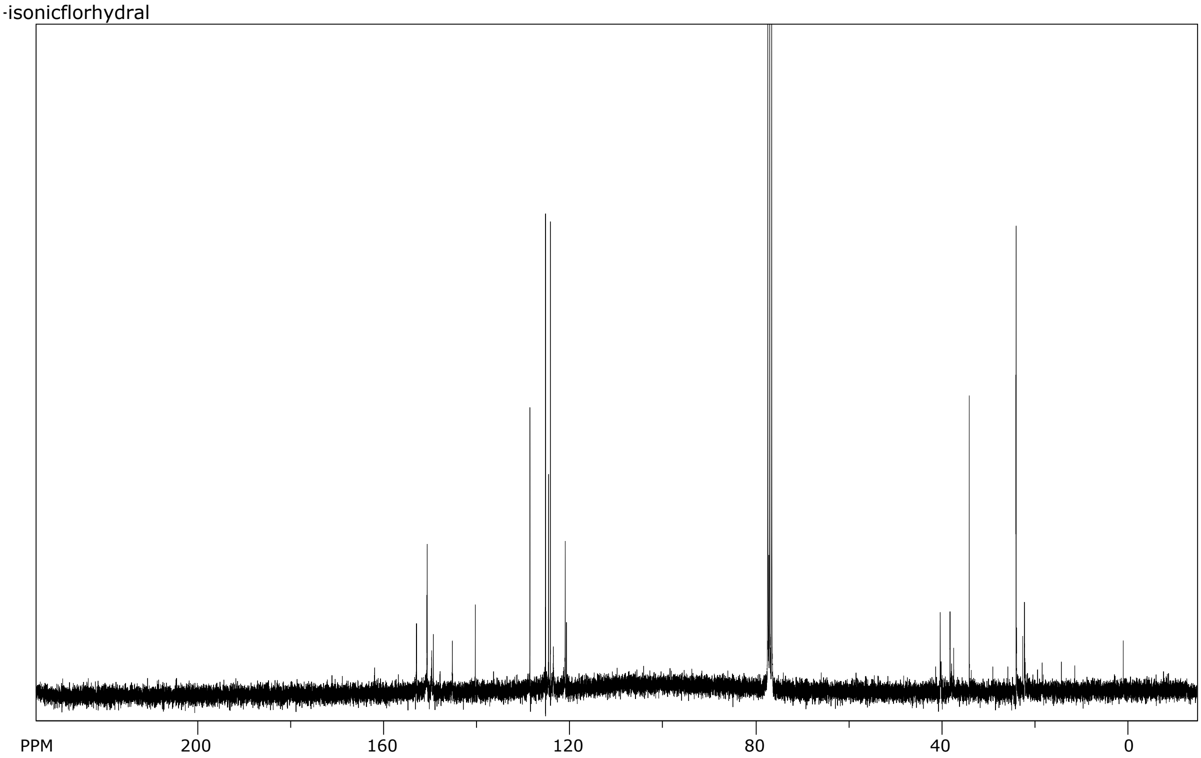

,2\fid expt: <zgpg30>
75.476050 MHz
: 32768 points
ppm = 0.574719 Hz/pt
scans: 67376

freq. of 0 ppm: 75.467751 MHz
processed size: 32768 complex points
LB: 0.000 GF: 0.0000

Figure S46. ^{13}C NMR spectrum of 4g.

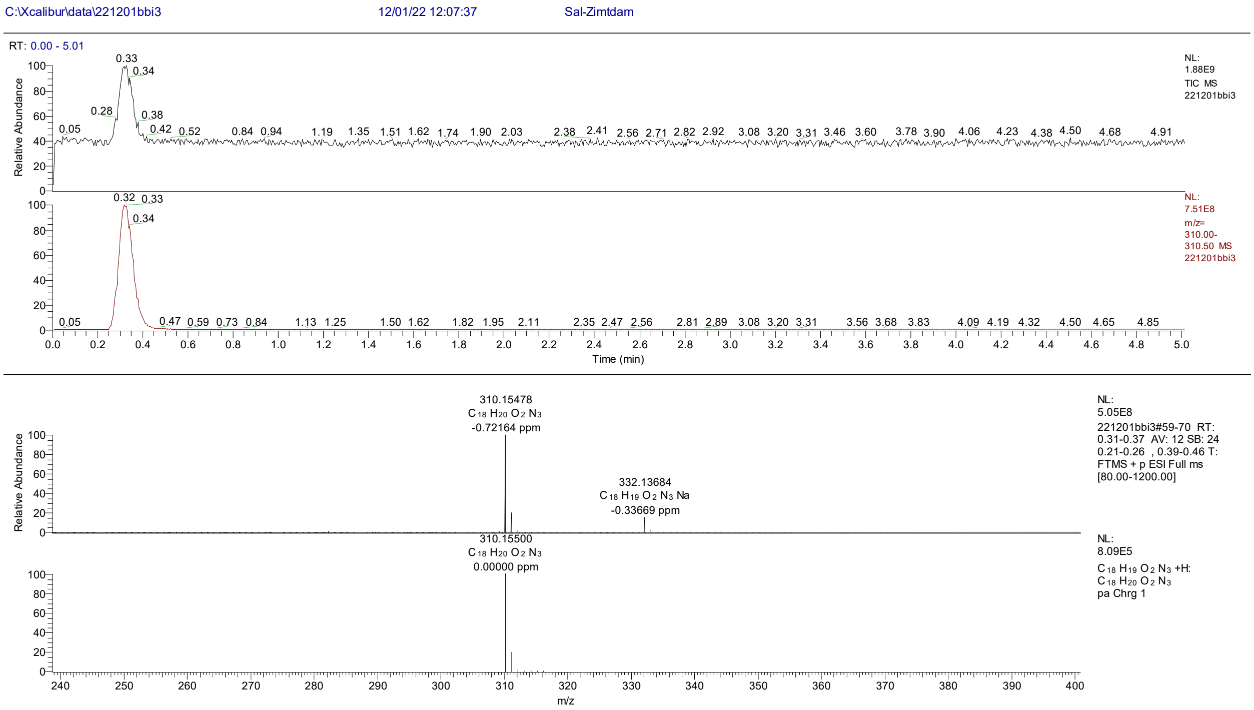

Figure S47. HRMS spectrum of 2d.

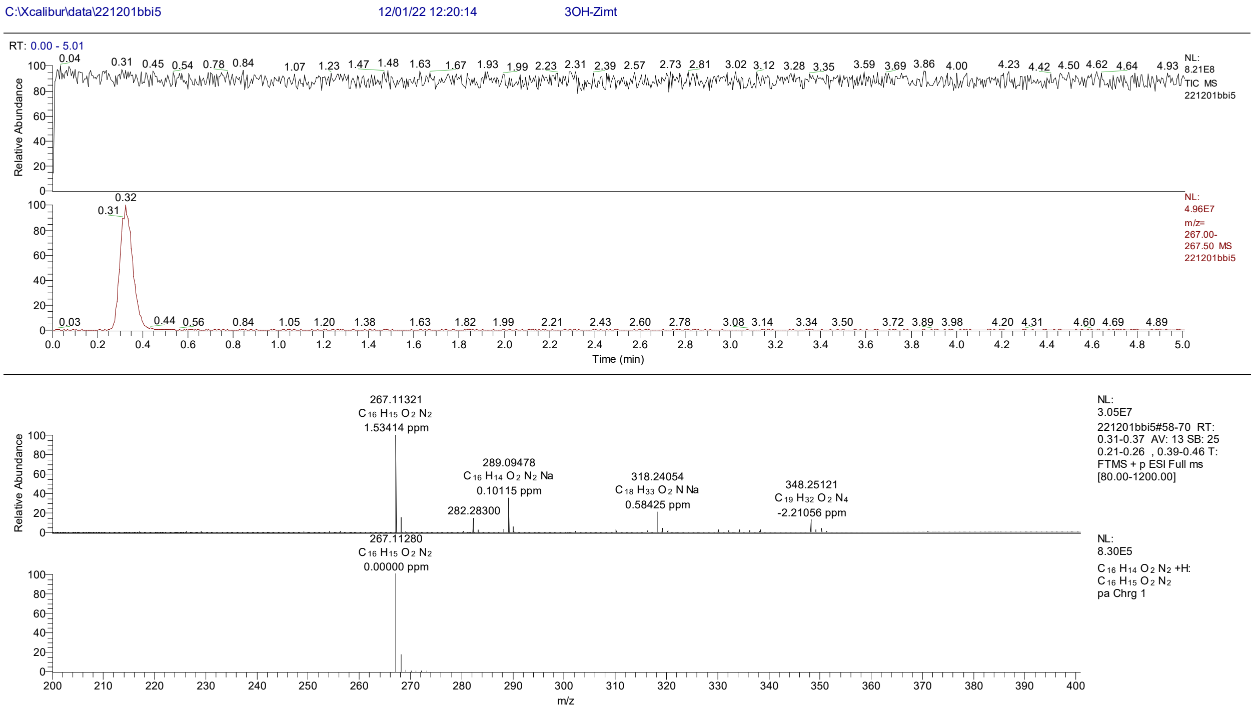

Figure S48. HRMS spectrum of 2e.

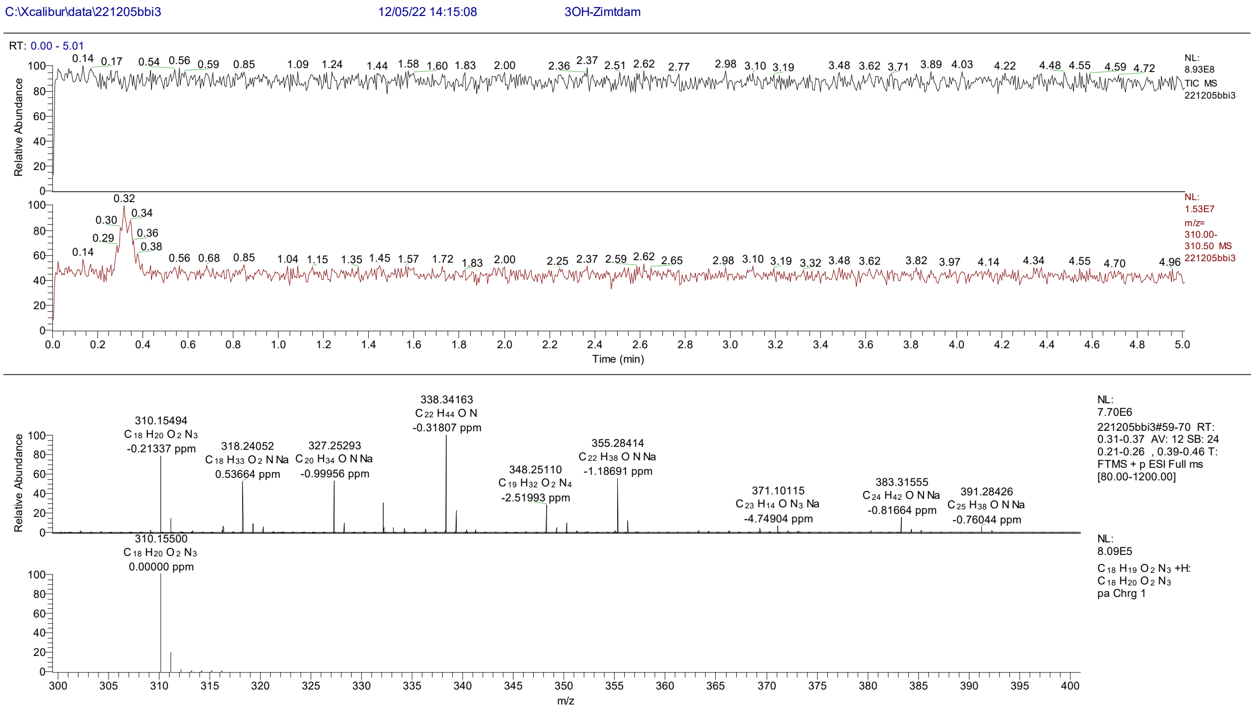

Figure S49. HRMS spectrum of 2f.

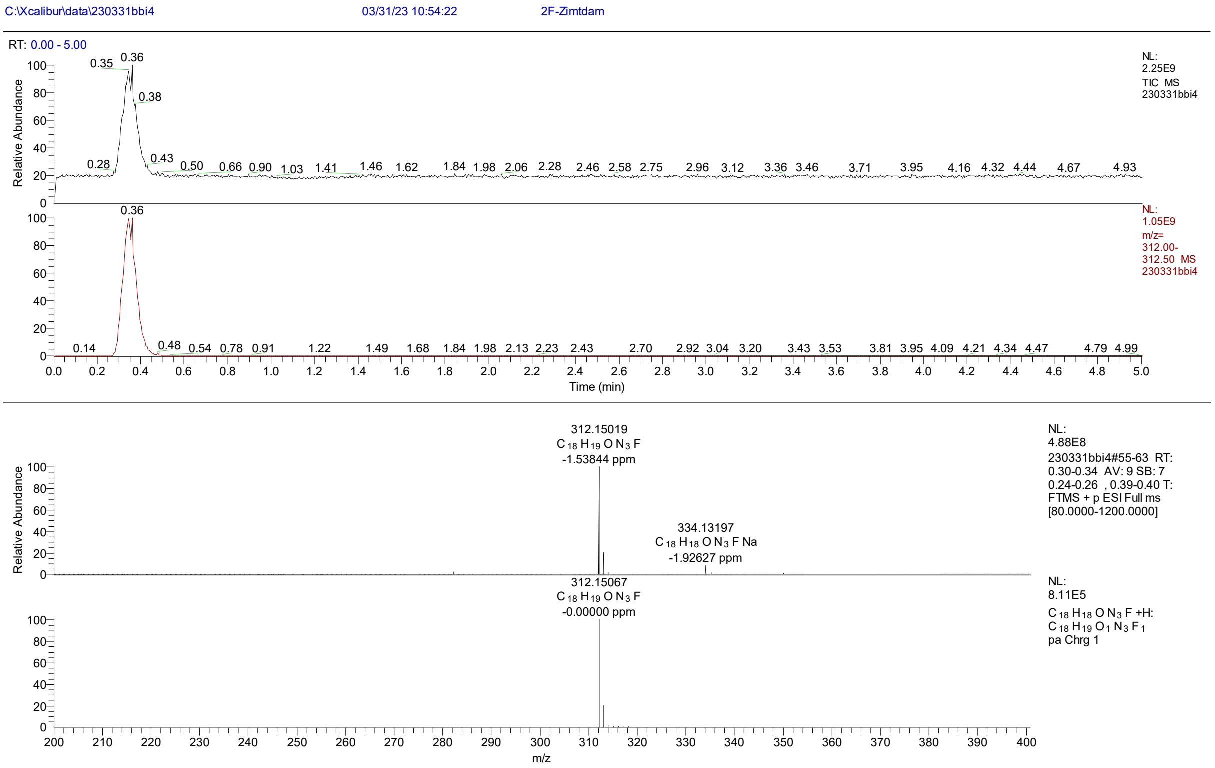

Figure S50. HRMS spectrum of 2h.

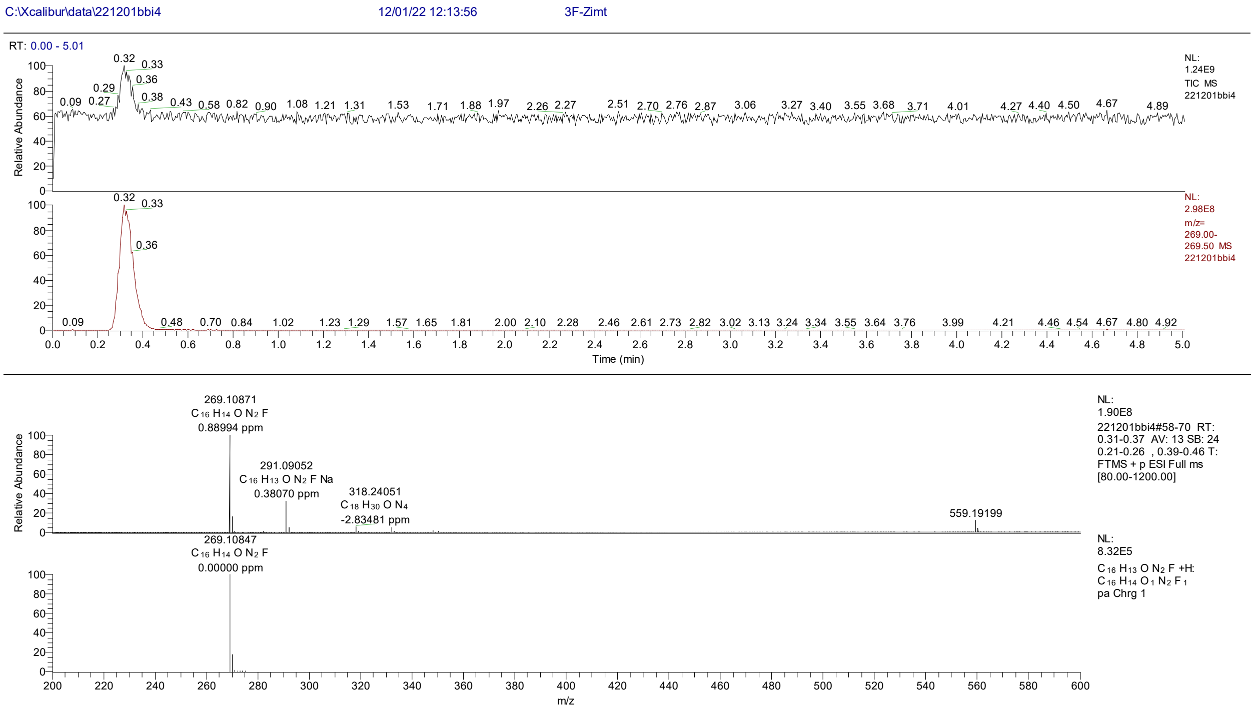

Figure S51. HRMS spectrum of 2i.

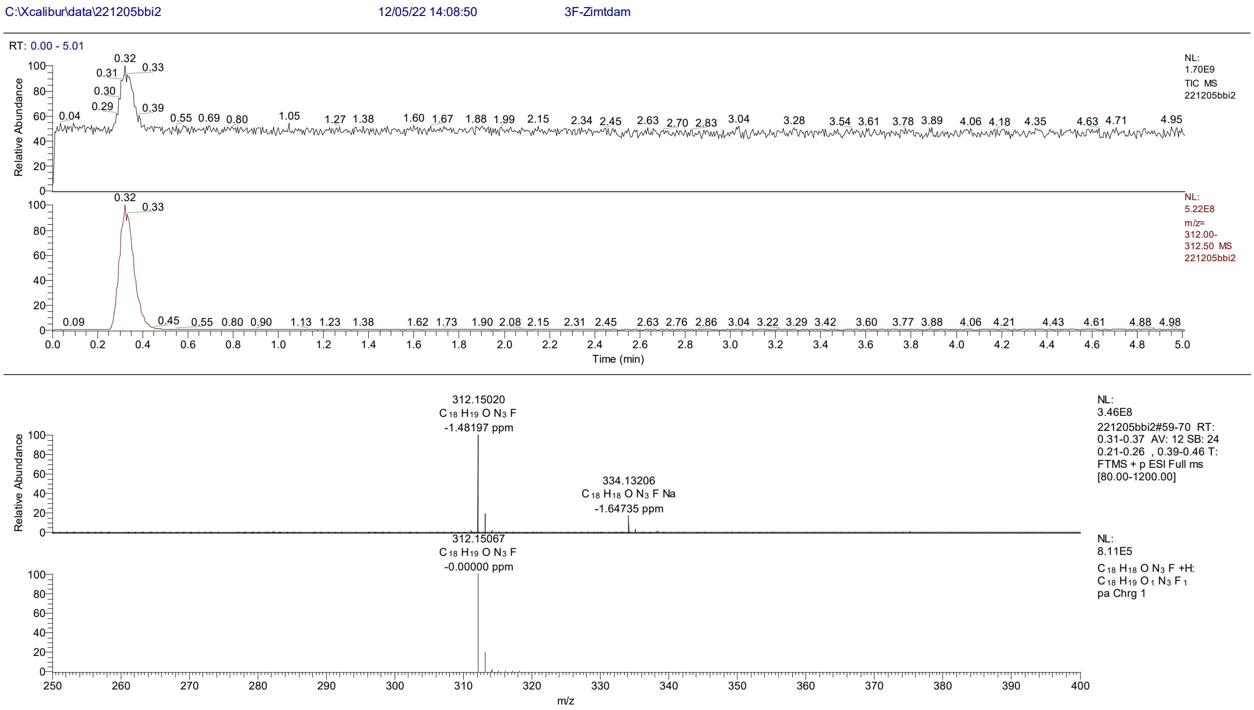

Figure S52. HRMS spectrum of 2j.

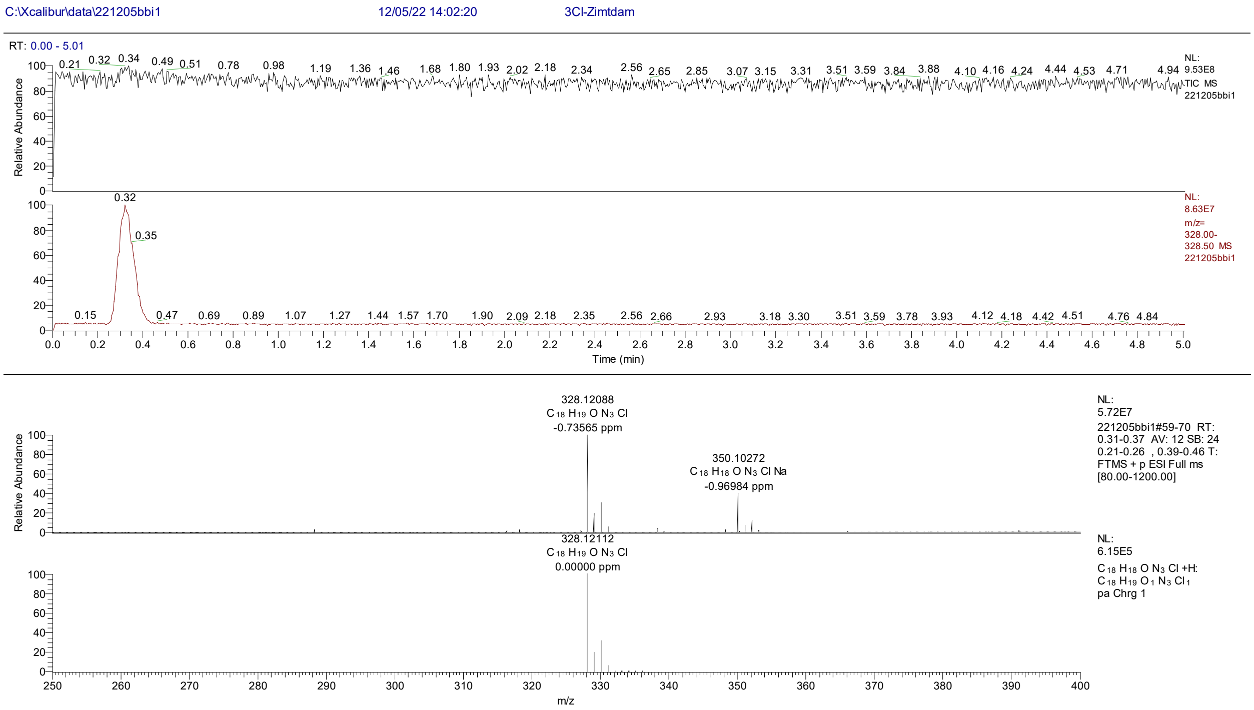

Figure S53. HRMS spectrum of 2k.

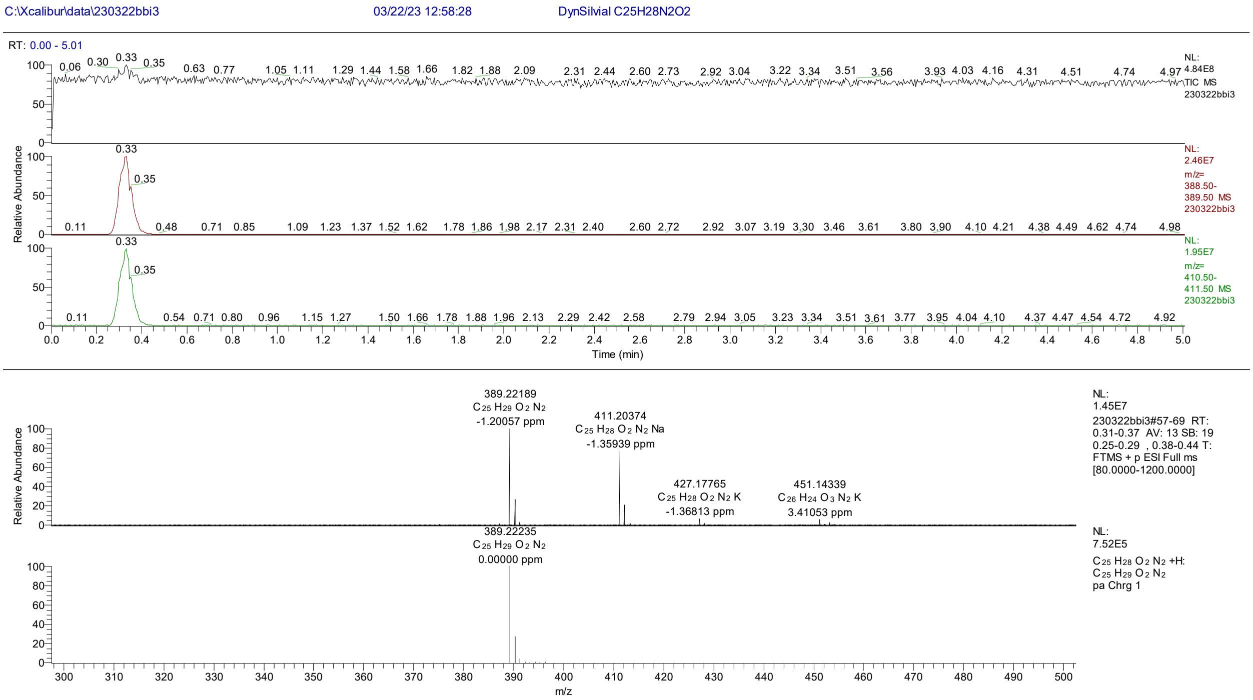

Figure S54. HRMS spectrum of 3a.

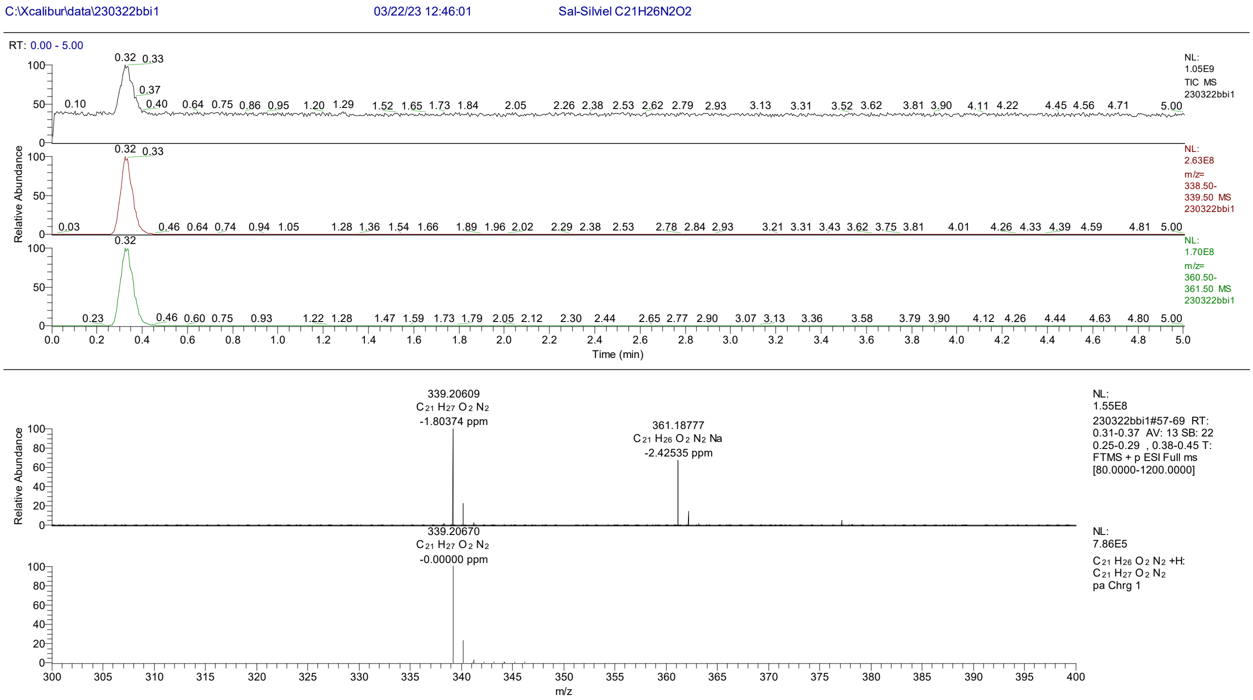

Figure S55. HRMS spectrum of 3b.

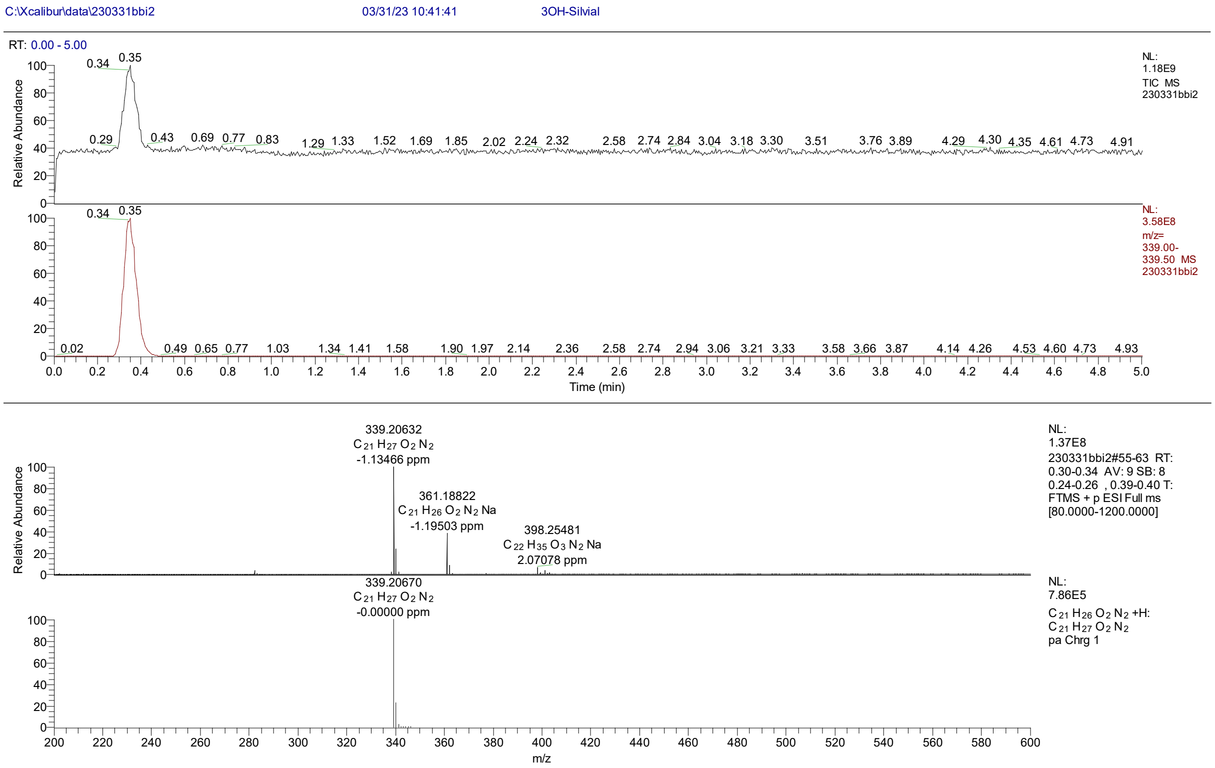

Figure S56. HRMS spectrum of 3c.

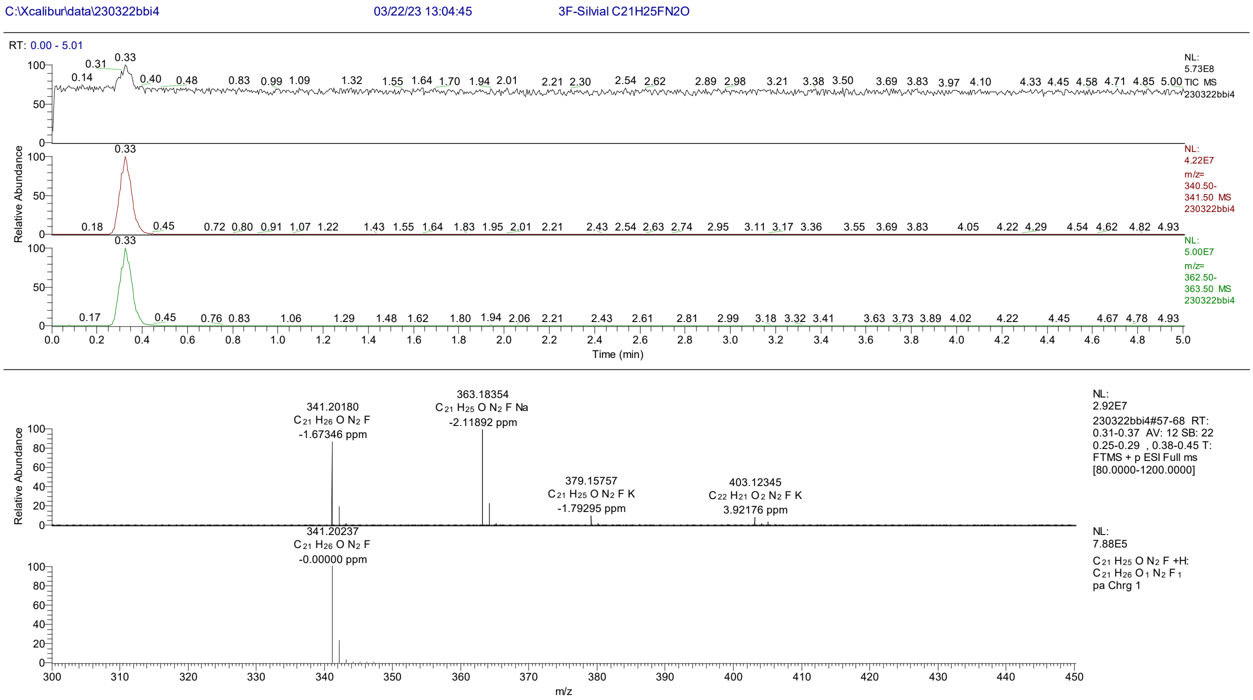

Figure S57. HRMS spectrum of 3d.

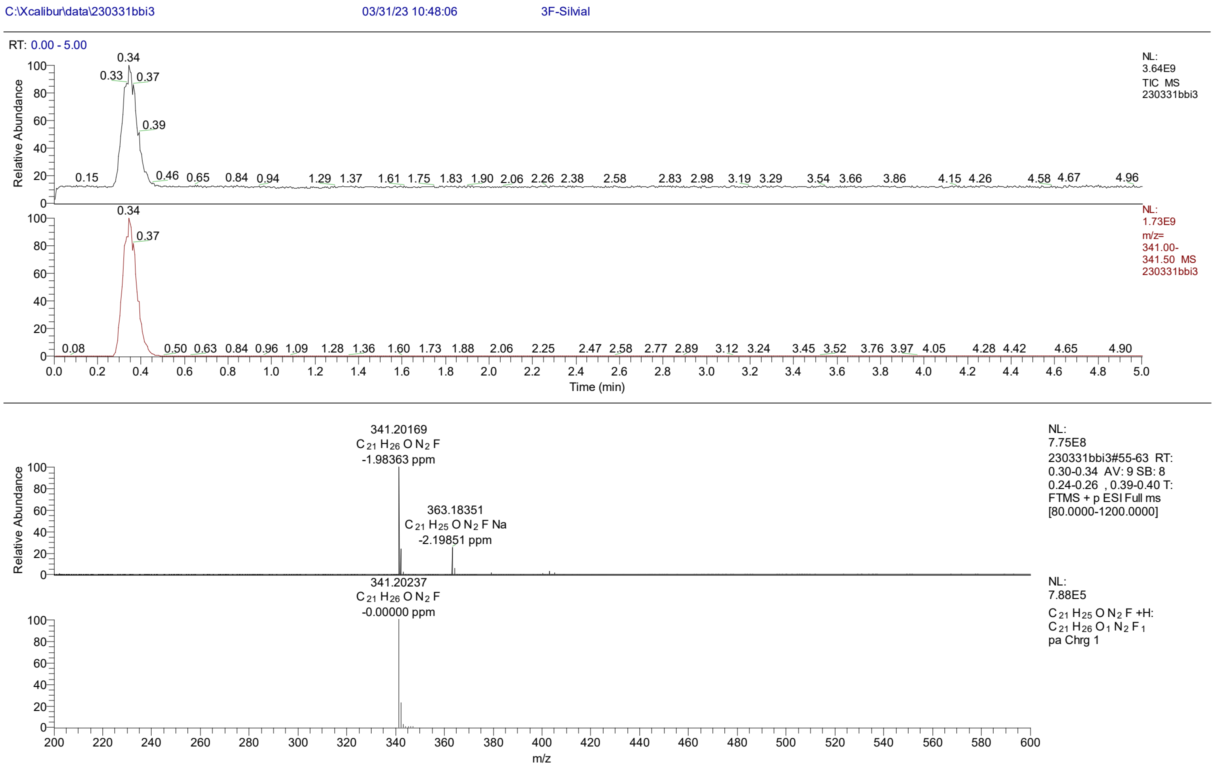

Figure S58. HRMS spectrum of 3e.

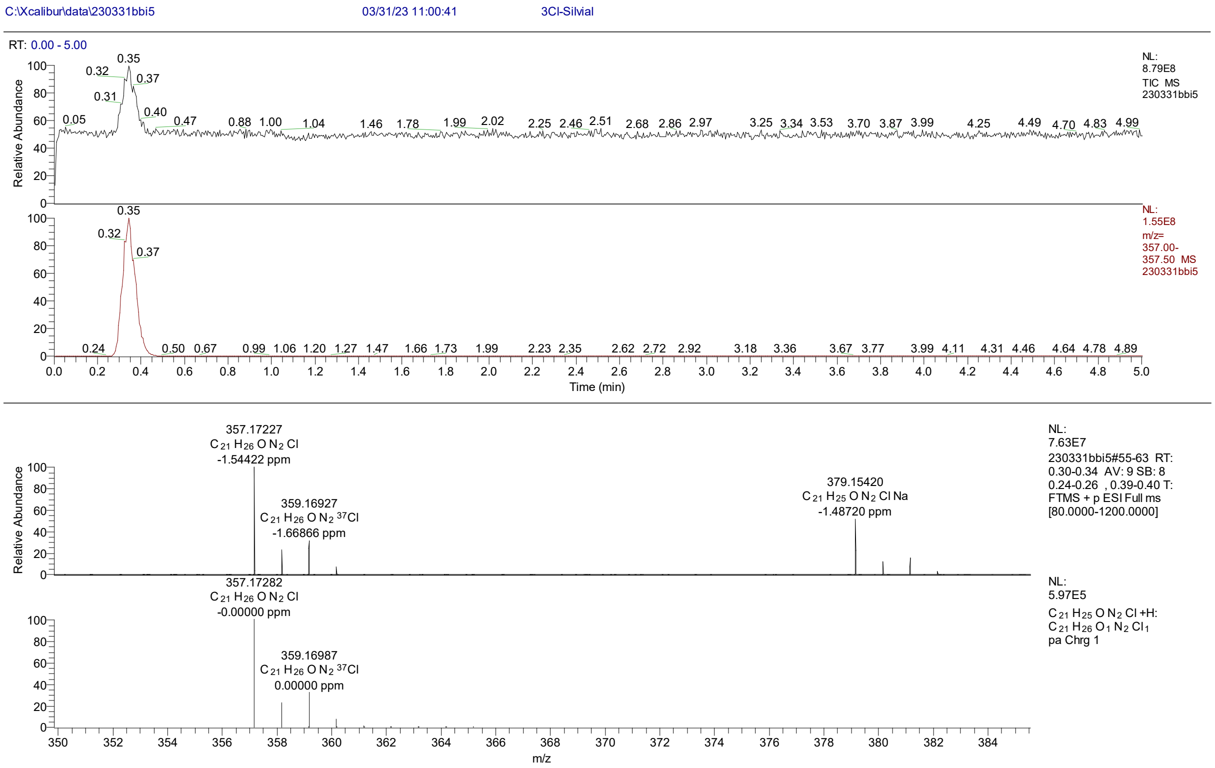

Figure S59. HRMS spectrum of 3f.

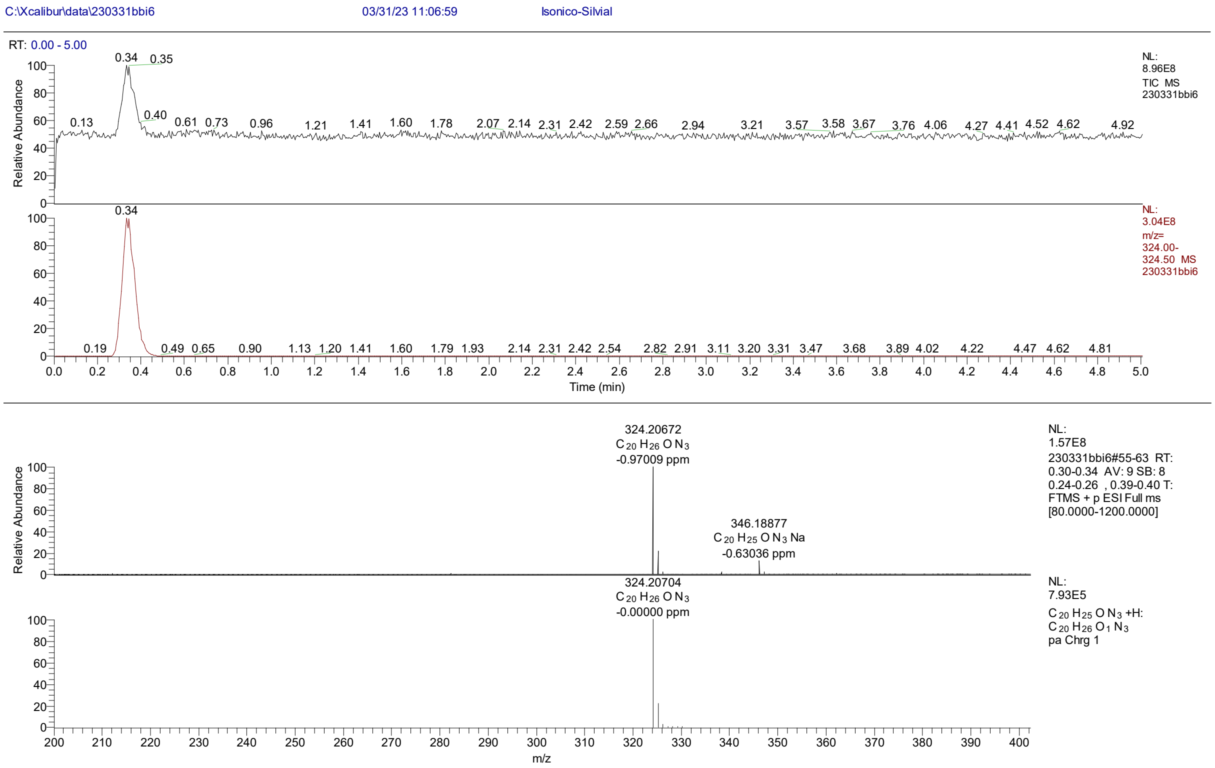

Figure S60. HRMS spectrum of 3g.

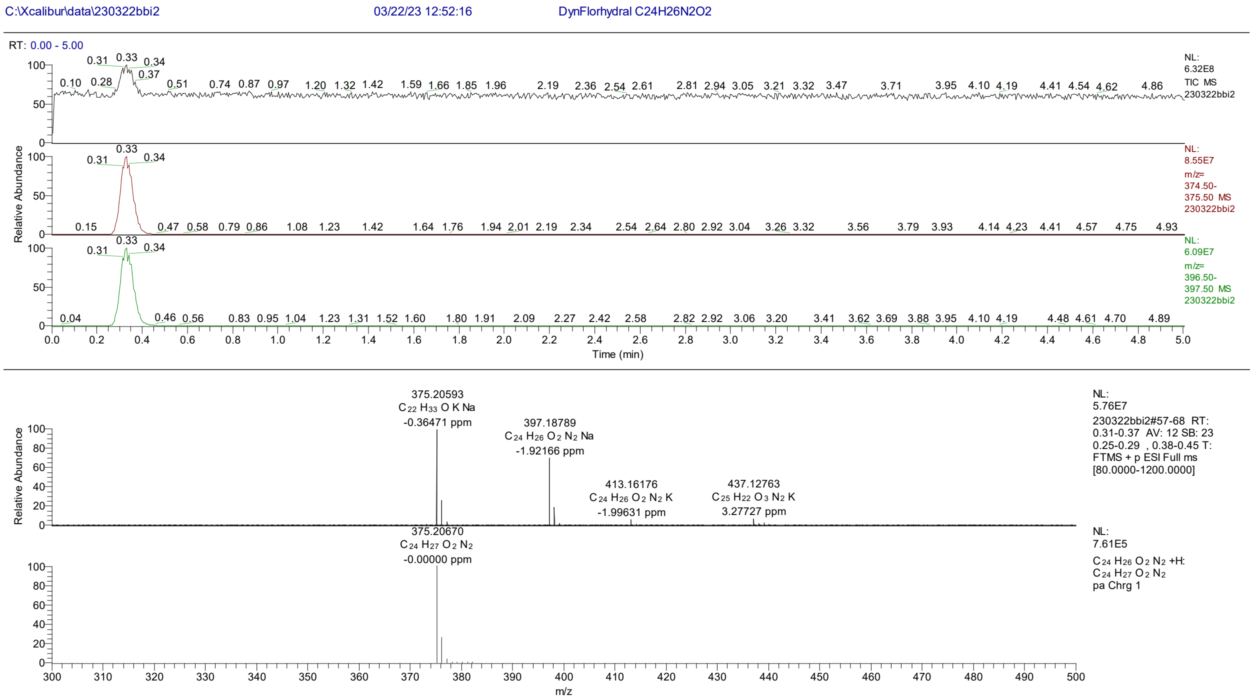

Figure S61. HRMS spectrum of 4a.

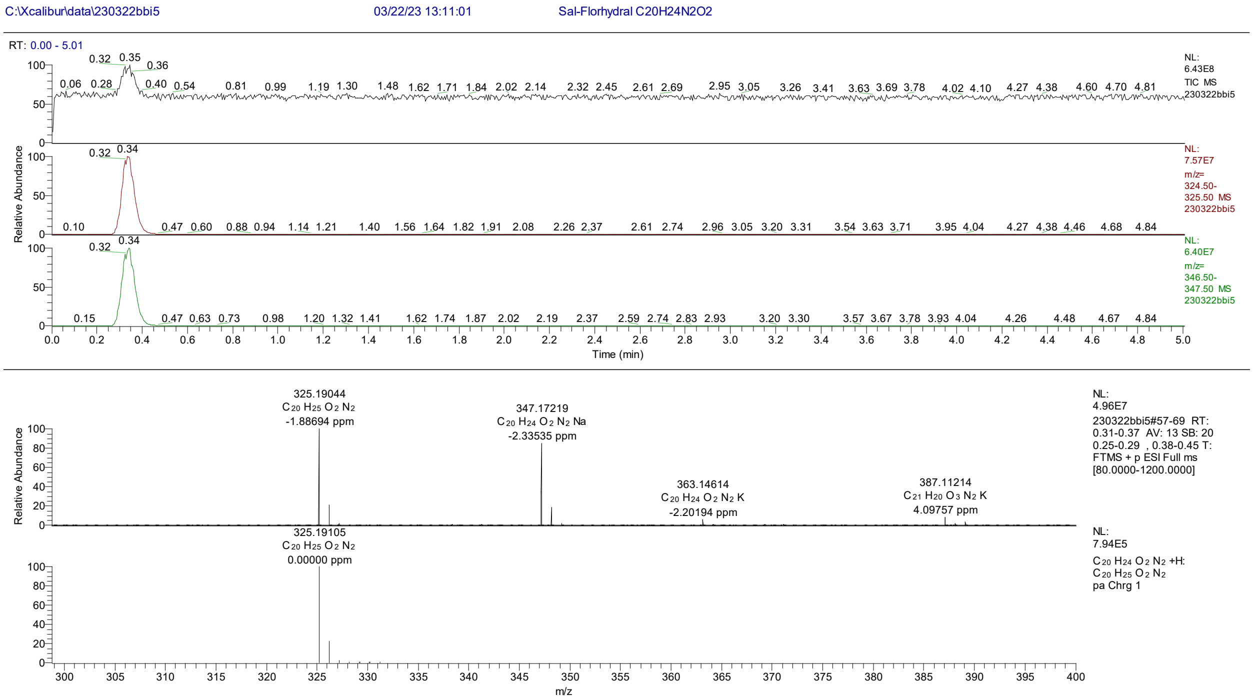

Figure S62. HRMS spectrum of 4b.

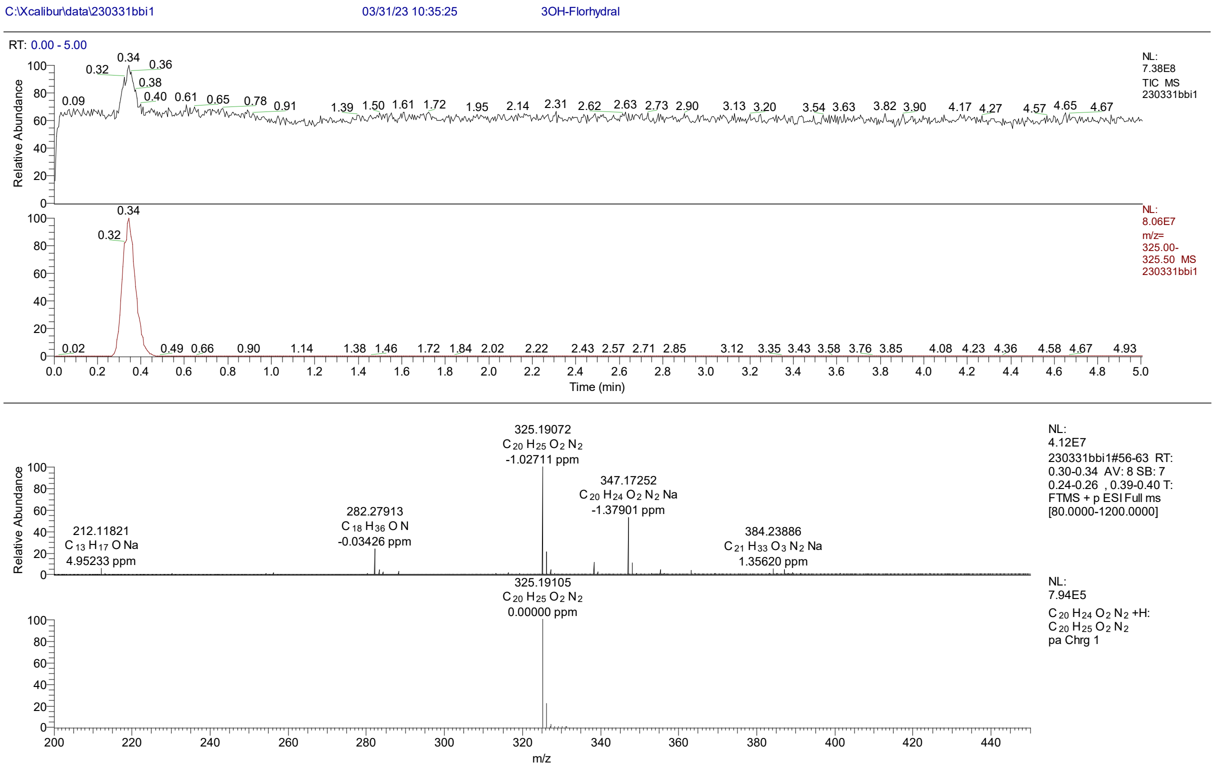

Figure S63. HRMS spectrum of 4c.

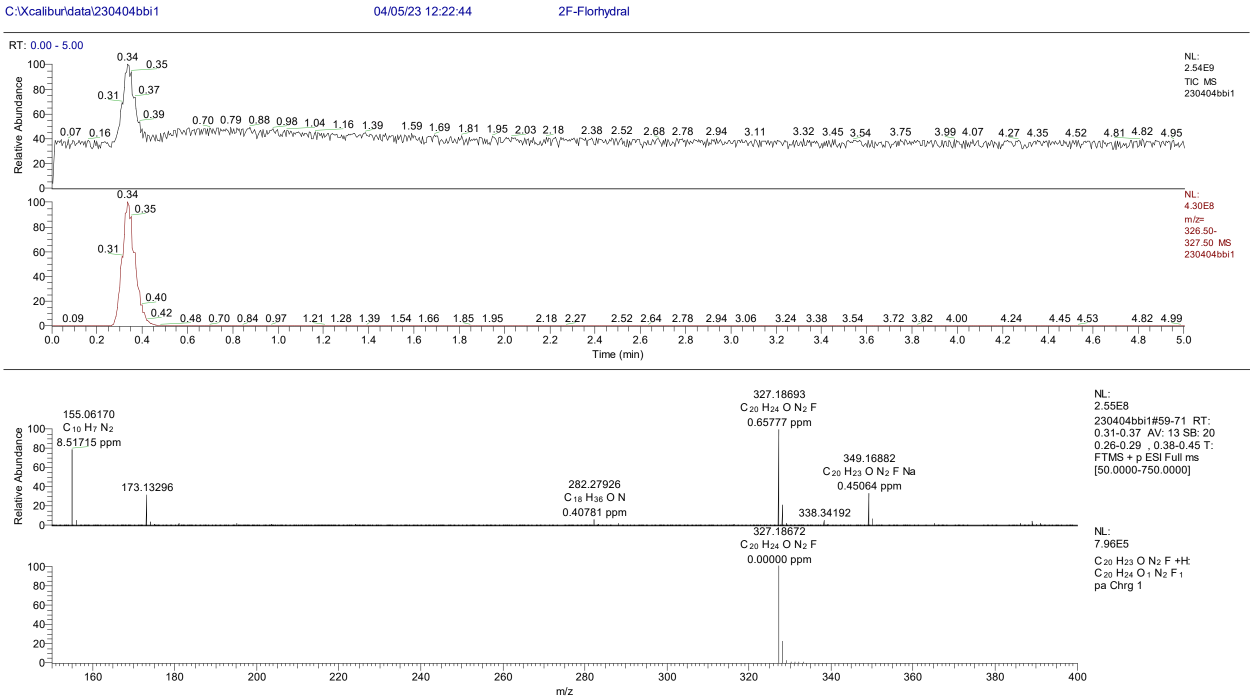

Figure S64. HRMS spectrum of 4d.

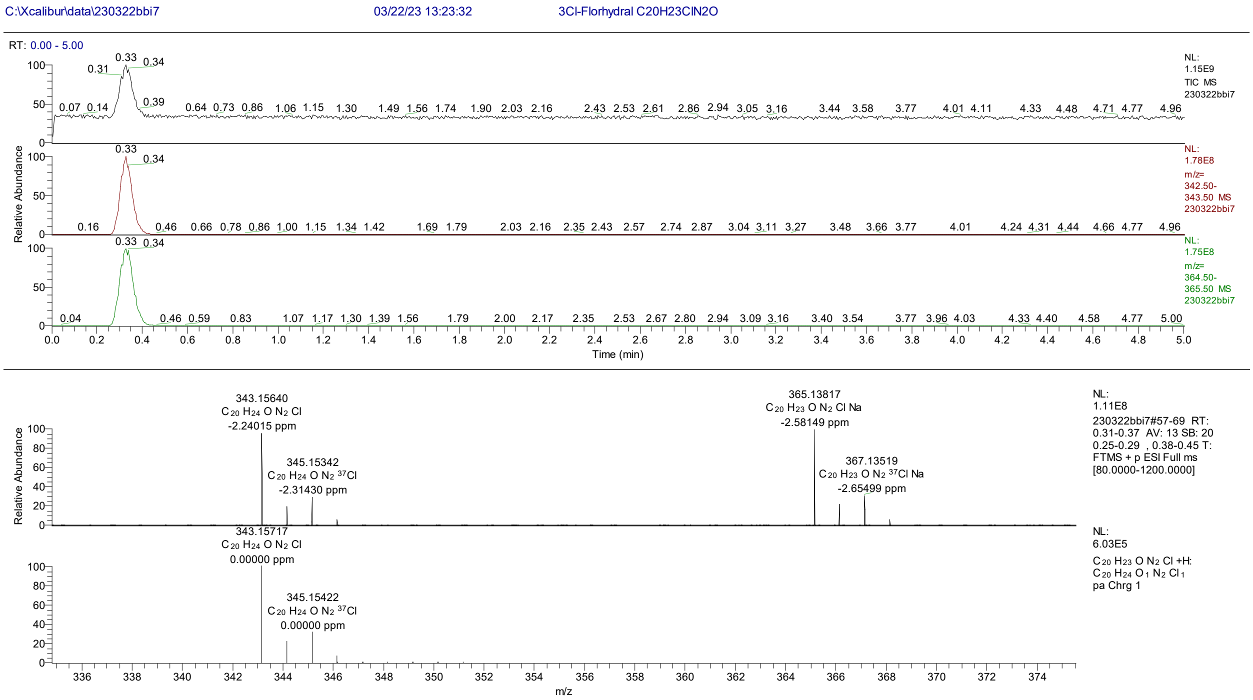

Figure S65. HRMS spectrum of 4f.

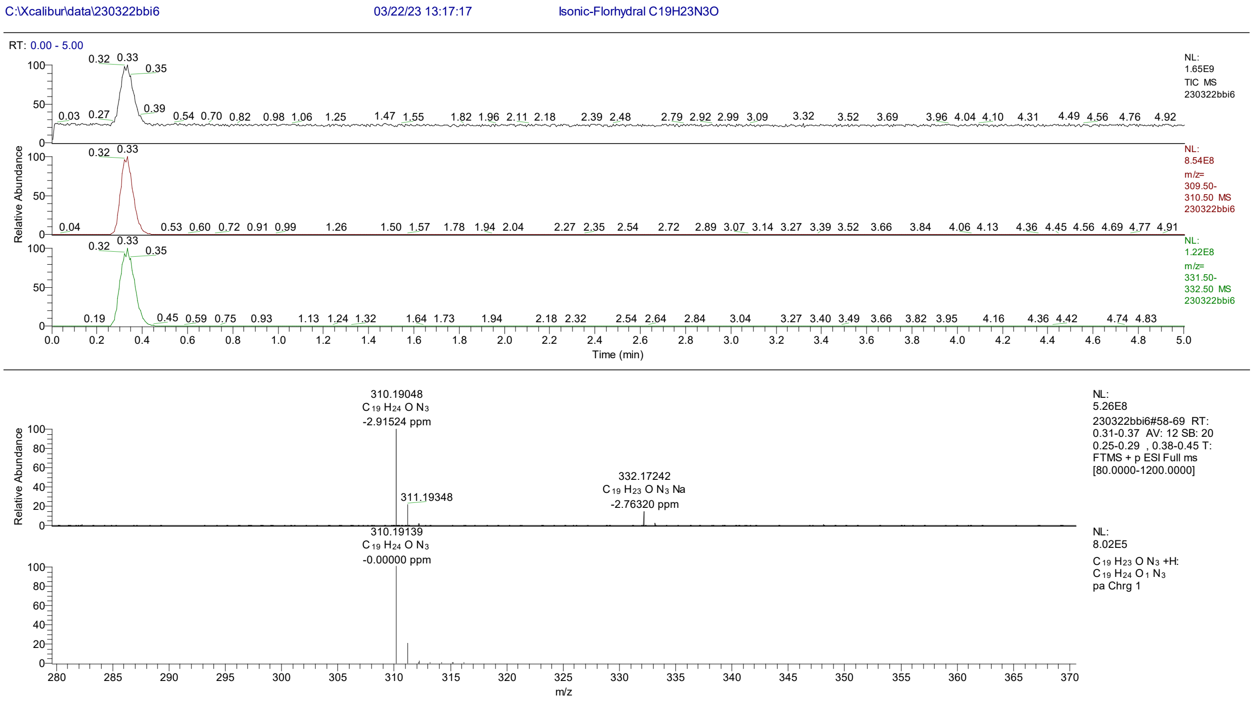

Figure S66. HRMS spectrum of 4g.
